# Advances in research on layered double hydroxides for biomedical applications

**DOI:** 10.1002/smo2.70094

**Published:** 2026-09-03

**Authors:** Xinhui Yang, Zhiping Zhang, Zhao Wang, Hao Zhang, Yen Leng Pak, Yingying Jing, Yurong Guo, Fang Li, Xiao Wang, Quanxiang Han, Lei Yu, Xuejiao Li, Xing Gao, Jibin Song

**Affiliations:** ^1^ Heilongjiang Provincial Key Laboratory of CO_2_ Resource Utilization and Energy Catalytic Materials School of Material Science and Chemical Engineering Harbin University of Science and Technology Harbin China; ^2^ School of Biological and Chemical Engineering Qilu Institute of Technology Jinan China; ^3^ State Key Laboratory of Chemical Resource Engineering College of Chemistry Beijing University of Chemical Technology Beijing China

**Keywords:** biomedical application, layered double hydroxides, synthetic approaches, theranostic platform

## Abstract

Layered double hydroxides (LDHs), as a typical class of two‐dimensional layered nanomaterials, have attracted extensive attention in biomedical fields due to their unique layered structure, tunable chemical composition, good biocompatibility, ease of surface functionalization, and efficient drug loading and controlled release capabilities. This review systematically summarizes recent advances in the research on LDHs for biomedical applications, focusing on their potential in drug delivery, bioimaging, and other disease treatments. Studies demonstrate that LDHs function as efficient carriers for a range of bioactive molecules, including anticancer drugs, nucleic acids, and proteins. Targeted and controlled release is enabled through mechanisms like pH responsiveness and ion exchange. Their favorable biodegradability and low cytotoxicity provide a safety basis for in vivo applications. Moreover, LDHs exhibit remarkable advantages in photothermal therapy, synergistic therapy, and the construction of multifunctional theranostic platforms. Despite significant progresses, challenges remain regarding long‐term biosafety, scalable fabrication, and clinical translation. In the future, through rational design and multifunctional integration, LDHs are expected to become key components in next‐generation intelligent nanotheranostic platforms.

## Graphical abstract

Layered double hydroxides (LDHs) are pH‐responsive nanomaterials with broad biomedical applications. This review summarizes LDH synthesis methods and compares their advantages. It highlights LDHs' progress in cancer therapy, including chemo‐photothermal, chemo‐immunotherapy, and photodynamic‐immunotherapy combinations and their potential in bone repair. LDHs also enable multimodal biological imaging and modulate the immune microenvironment. Furthermore, they promote osteogenic differentiation, supporting bone tissue engineering.

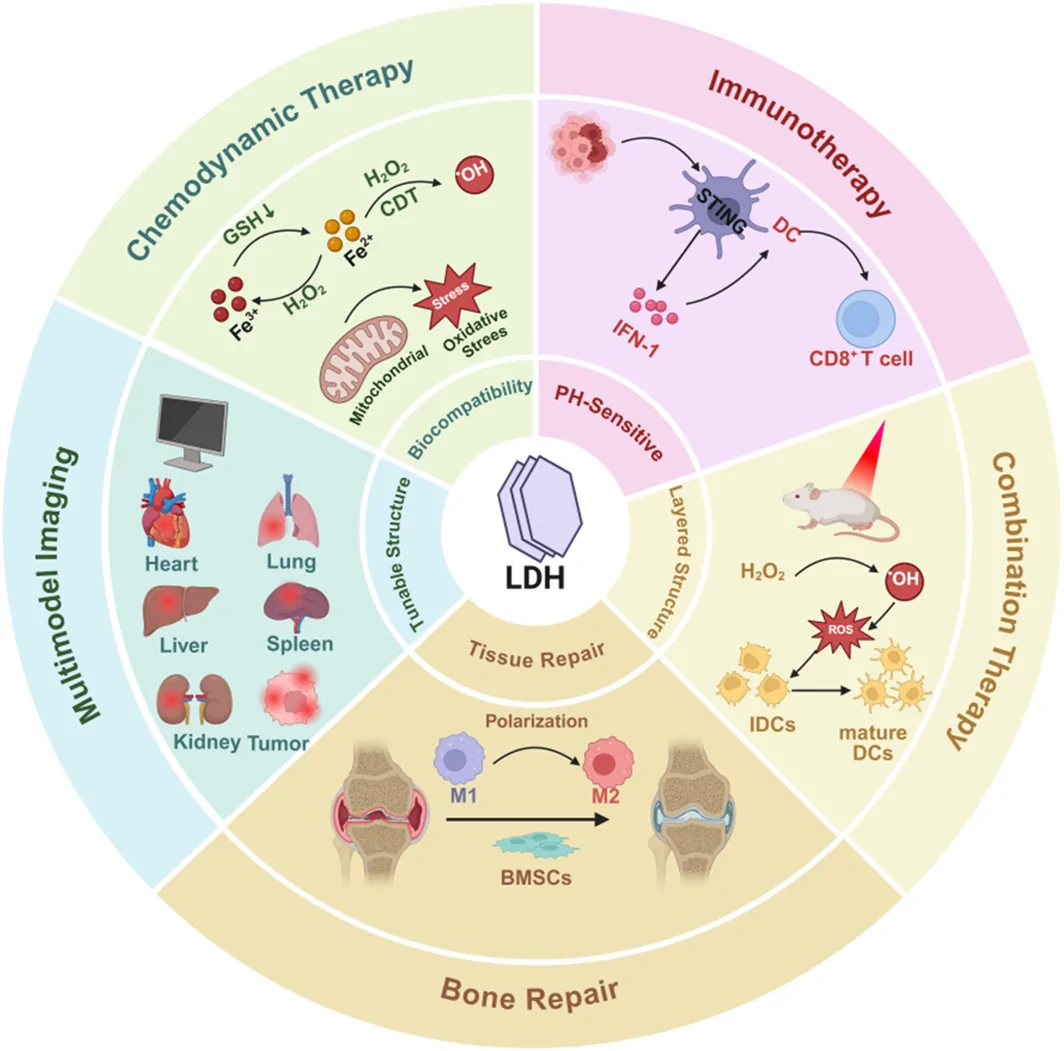

## 1 INTRODUCTION

Layered double hydroxides (LDHs), also known as layered double metal hydroxides, are rapidly emerging as highly attractive multifunctional platforms in the biomedical field due to their unique hydrotalcite‐like structure, highly tunable physicochemical properties, and excellent biocompatibility, demonstrating significant potential for translation from basic research to clinical applications.[^1^, ^2^, ^3^] Their structural origin can be traced back to the natural mineral hydrotalcite (Mg_6_Al_2_(OH)_16_CO_3_·4H_2_O), featuring a positively charged brucite‐like layered structure formed by edge‐sharing octahedral coordination of divalent metal cations (e.g., Mg^2+^, Zn^2+^, Co^2+^, Ni^2+^, Fe^2+^) and trivalent metal cations (e.g., Al^3+^, Fe^3+^, Cr^3+^, Ga^3+^).[^4^, ^5^, ^6^] The general chemical formula [$M_{1‐x}^{2+}M_{x}^{3+}$(OH)_2_]^x+^[A^n−^]_x/n_·mH_2_O, where the value of *x* (the molar ratio of M^3+^/(M^3+^+M^3+^), typically 0.2 ≤ *x* ≤ 0.33) precisely regulates the layer charge density.^[7]^ Mean, the interlayer gallery accommodates exchangeable anions and water molecules to maintain electrical neutrality. This unique “sandwich‐like” supramolecular assembly structure endows LDHs with a series of crucial physicochemical properties in biomedical applications.[^8^, ^9^]

LDHs possess excellent dynamic anion exchange capability where interlayer anions are bound to the positively charged layers through relatively weak electrostatic attraction and hydrogen bonding. They can be efficiently displaced by various bioactive anions, such as chemotherapeutic drug molecules (methotrexate, 5‐fluorouracil), nucleic acids (siRNA, DNA), polypeptides, and even contrast agent anions.[^10^, ^11^, ^12^] Moreover, the excellent biocompatibility and low cytotoxicity of LDHs serve as a key guarantee for their clinical translation potential. The main constituent elements of LDHs are naturally occurring metal ions in the human body, and their degradation products typically exhibit low biological toxicity and can be metabolized and eliminated by the body.[^13^, ^14^] A multitude of in vitro cell experiments (such as MTT assays) and in vivo model studies have verified that, within a rationally designed dosage range, LDHs demonstrate favorable hemocompatibility, low immunogenicity, and acceptable systemic toxicity, significantly outperforming many inorganic or organic polymer nanocarriers.[^15^, ^16^, ^17^]

The research progress of LDHs in the biomedical field has expanded from fundamental material synthesis to diversified application scenarios in the diagnosis and treatment of complex diseases.[^18^, ^19^] In terms of targeted drug delivery, LDHs, as smart carriers, are widely used for the efficient loading and controlled release of chemotherapeutic drugs (e.g., doxorubicin, cisplatin derivatives), gene therapy molecules (siRNA for gene silencing, plasmid DNA for gene expression), and biomacromolecules (proteins, polypeptides).[^20^, ^21^] Through surface modification with targeting molecules (such as folate receptor targeting), their active accumulation at tumor sites can be significantly enhanced, and precision therapy can be achieved via pH‐responsive or NIR light‐triggered release. Beyond their role as delivery vehicles, LDHs represent an ideal synergistic therapeutic platform capable of achieving spatiotemporally coordinated sensitization, thereby significantly overcoming the limitations of monotherapy and tumor resistance.[^22^, ^23^, ^24^, ^25^, ^26^, ^27^] Notably, LDH‐mediated local therapy can effectively induce immunogenic cell death (ICD) in tumor cells, releasing damage‐associated molecular patterns (DAMPs) such as calreticulin (CRT), high‐mobility group box 1 protein (HMGB1), and ATP. This process activates dendritic cells (DCs) and promotes tumor antigen presentation, thereby initiating T cell‐mediated anti‐tumor immune responses.[^28^, ^29^] The combination of drug‐loaded LDHs with immune checkpoint blockers (e.g., anti‐PD‐1/PD‐L1 antibodies) can significantly reverse the tumor immunosuppressive microenvironment (TME) and enhance the infiltration and function of cytotoxic T lymphocytes (CTLs). This combination not only inhibits orthotopic tumor growth but also effectively suppresses distant metastases and induces long‐term immune memory effects, demonstrating robust systemic anti‐tumor potential.[^30^, ^31^]

In addition, LDHs, serving as bioimaging contrast agents or their nano‐carriers, have significantly improved diagnostic accuracy, sensitivity, and versatility in the molecular imaging diagnosis.^[32]^ By intercalating magnetic ions (e.g., Gd^3+^, Mn^2+^) into the interlayers or loading superparamagnetic iron oxide nanoparticles,[^33^, ^34^] LDHs can serve as high‐performance *T*_1_/*T*_2_‐weighted magnetic resonance imaging (MRI) contrast agents; doping with lanthanide elements (e.g., Eu^3+^, Tb^3+^) or loading near‐infrared fluorescent dyes (e.g., Cy7, IR825) enables their use in high‐sensitivity fluorescence imaging (FL) or second near‐infrared window (NIR‐II) imaging; while loading with radionuclides (e.g., ^99m^Tc, ^64^Cu) makes them suitable for single‐photon emission computed tomography (SPECT) or positron emission tomography (PET).[^35^, ^36^, ^37^, ^38^, ^39^, ^40^, ^41^] This multimodal imaging capability enables high‐precision visualization of the location, size, boundaries, and microenvironmental characteristics of tumors.

**FIGURE 1:**
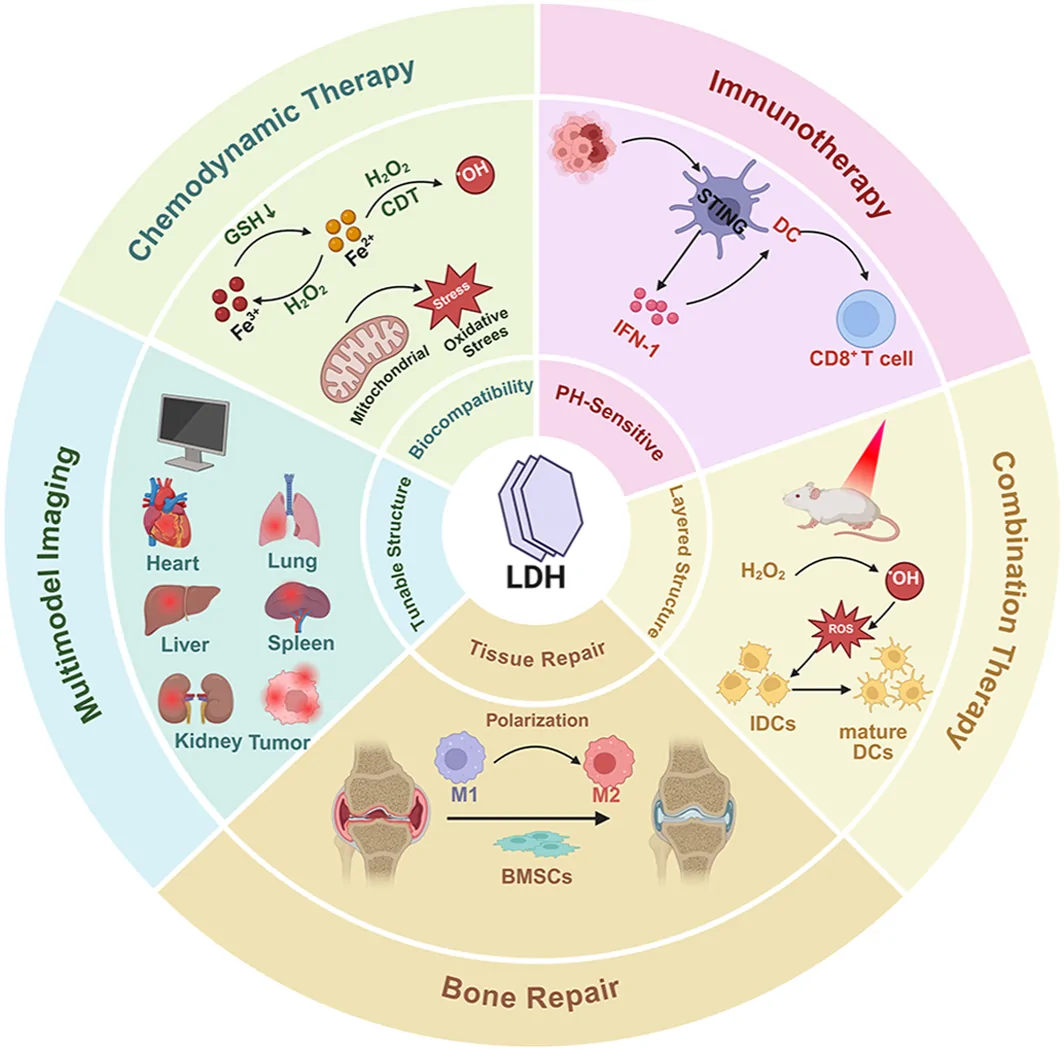
Schematic demonstration of the properties and advantages of LDH‐based nanomaterials for biomedical applications.

This review aims to provide a comprehensive and in‐depth summary of the research progress on LDHs in the field of biomedicine. (as shown in Figure 1). First, the synthesis methods of LDHs are categorized and summarized, and their unique advantages as biomedical materials are analyzed. Second, various synthesis strategies and surface functionalization approaches for LDHs are systematically elaborated, offering guidance for the construction of high‐performance biomedical platforms. Subsequently, the latest advancements in the applications of LDHs are highlighted with a focus on drug delivery, tumor therapy, bone repair, and bioimaging, demonstrating their potential as versatile nanoplatforms. Finally, based on the current state of research, the key challenges and future directions for this material are discussed, aiming to provide a theoretical basis and innovative insights to promote the practical application of LDHs in precision biomedicine.

## 2 SYNTHESIS OF LDHS

### 2.1 Bottom‐up

#### 2.1.1 Co‐precipitation

The discovery of LDHs can be traced back to 1842,^[42]^ when Hochstetter initially identified natural hydrotalcite minerals in schist. Subsequently, in 1942, the co‐precipitation method was first reported for the synthesis of MgAl‐LDHs.^[43]^ Due to its operational simplicity, cost‐effectiveness, and tunable composition, this method has become the most representative approach for the preparation of LDHs. In a typical preparation procedure, divalent and trivalent metal salts are formulated into a mixed‐salt solution in accordance with a specific molar ratio. This solution is then slowly dripped into an alkaline solution containing the target anions. The reaction proceeds under strict temperature control (60–80°C), with the pH maintained between 6 and 11 by dropwise addition of NaOH solution.[^44^, ^45^] This method operates under mild conditions with simple equipment, facilitating both laboratory and industrial production. More importantly, it allows for precise design of the layer composition and interlayer structure by controlling the types and ratios of metal ions and the choice of intercalated anions.^[46]^

**FIGURE 2:**
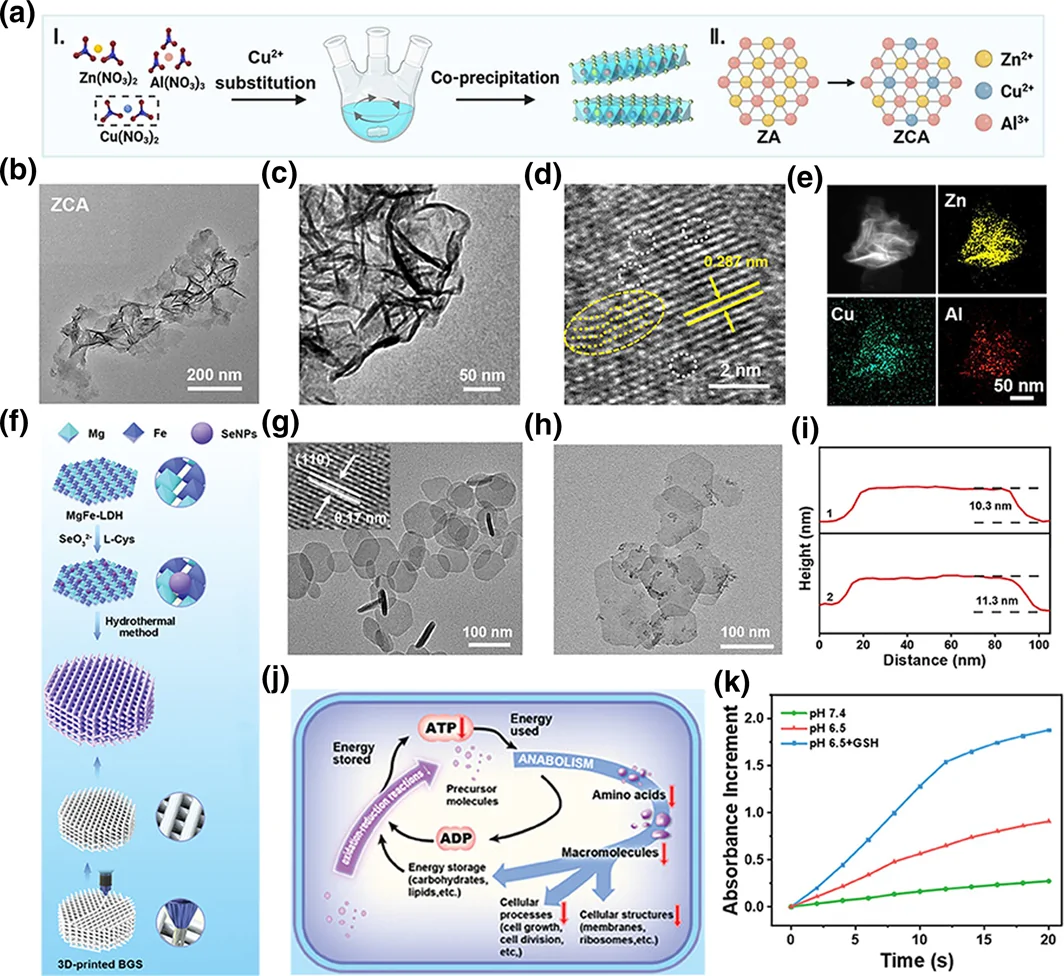
Schematic diagram of the regulation of Layered double hydroxides (LDHs) structure and properties by co‐precipitation method. (a) Schematic illustration of the synthetic procedure of ZCA NSs. (b), (c) TEM images of the as‐synthesized ZCA NSs at different magnifications. (d) HRTEM image and lattice spacing of ZCA NSs. The subtle distortion regions of ZCA NSs and vacancy defects are marked by dashed and dotted circles, respectively. (e) Elemental mapping (Zn, Cu, and Al) of ZCA NSs. Reproduced with permission.^[49]^ Copyright 2024, American Chemical Society. (f) Synthetic Procedure for A Se Nanoparticle/MgFe‐LDH Composite Nanosheet. TEM image of (g) MgFe‐LDH and (h) LDH/Se nanosheets. (i) Corresponding height profiles of MgFe‐LDH nanosheets. (j) The mechanism diagram of LDH/Se in bone repair. (k) The absorbance of ox‐TMB at 652 nm in the presence of LDH/Se under different conditions: H_2_O_2_ (pH 7.4), H_2_O_2_ (pH 6.5), and H_2_O_2_ + GSH (pH 6.5). Reproduced with permission.^[48]^ Copyright 2024, Wiley‐VCH.

At room temperature, Shi added an aqueous NaOH solution dropwise to a mixed solution of Mg(NO_3_)_2_·6H_2_O and Al(NO_3_)_3_·9H_2_O, adjusted the pH to 9.5 ± 0.2, stirred the mixture under a nitrogen atmosphere for 48 hours, and then centrifuged it to collect the precipitate.^[47]^ Weng et al. dissolved Mg^2+^ and Fe^3+^ nitrates in deionized water.^[48]^ They then slowly added this nitrate solution, along with a NaOH solution, into a NaNO_3_ solution. After stirring at room temperature for 30 minutes, monodisperse hexagonal MgFe‐LDH nanosheets with a diameter of 60–110 nm were obtained (Figure 2f‐k). Similarly, Cheng successfully synthesized ZnCuAl‐LDH and ZnAl‐LDH using this method, and comparative studies revealed that the introduction of Cu^2+^ induced extensive lattice distortion and atomic disorder.^[49]^ These abundant defects provide additional active sites for ROS generation and optimize the electronic structure, thereby endowing ZnCuAl‐LDH with excellent sonodynamic properties under US irradiation. Benefiting from the tunable interlayer spacing of hydrotalcite, the coprecipitation method often incorporates specific organic molecules as intercalating agents during synthesis (Figure 2a–e). This strategy not only facilitates precise regulation of the interlayer distance but also effectively enhances the properties of the material, thereby providing a significant strategy for developing functionalized hydrotalcite materials. Wang added the metal solution and NaOH solution drop‐by‐drop into the stirring ZnPcG_4_‐FA solution under a nitrogen atmosphere, thereby obtaining a novel stimuli‐responsive multifunctional nanoplatform in which folate‐conjugated ZnPcG_4_ molecules (ZnPcG_4_‐FA) are intercalated into LDHs.^[50]^

Although the co‐precipitation method is simple to operate and has low cost, during the synthesis of LDHs, it is crucial to carefully control the pH range to prevent the formation of mixed phases due to the differences in precipitation conditions of various metal ions. Moreover, rapid nucleation often leads to the aggregation of nanosheets, limiting the control ability of this method in the two‐dimensional structure. In the actual synthesis process, the selection of interlayer anions is limited by thermodynamics, and strong acidic anions (such as PO_4_^3−^) will cause layer etching during the intercalation process.[^51^, ^52^, ^53^, ^54^, ^55^] To prevent such events from occurring and to obtain LDHs with the targeted anion intercalation, researchers use inert gases for protection, but this also increases the complexity of the experiment.

#### 2.1.2 Hydrothermal synthesis

Hydrothermal synthesis of LDHs represents a synthesis strategy that regulates crystal growth through a high temperature and pressure aqueous environment. This process involves crucial steps, including coprecipitation of metal salts and alkaline sources within a sealed autoclave, dissolution‐recrystallization, and anion intercalation. In comparison with other methods, the hydrothermal approach offers the advantage of producing products with high crystallinity, well‐defined layered structures, few defects, and controllable morphologies (such as hexagonal platelets or hollow structures).

**FIGURE 3:**
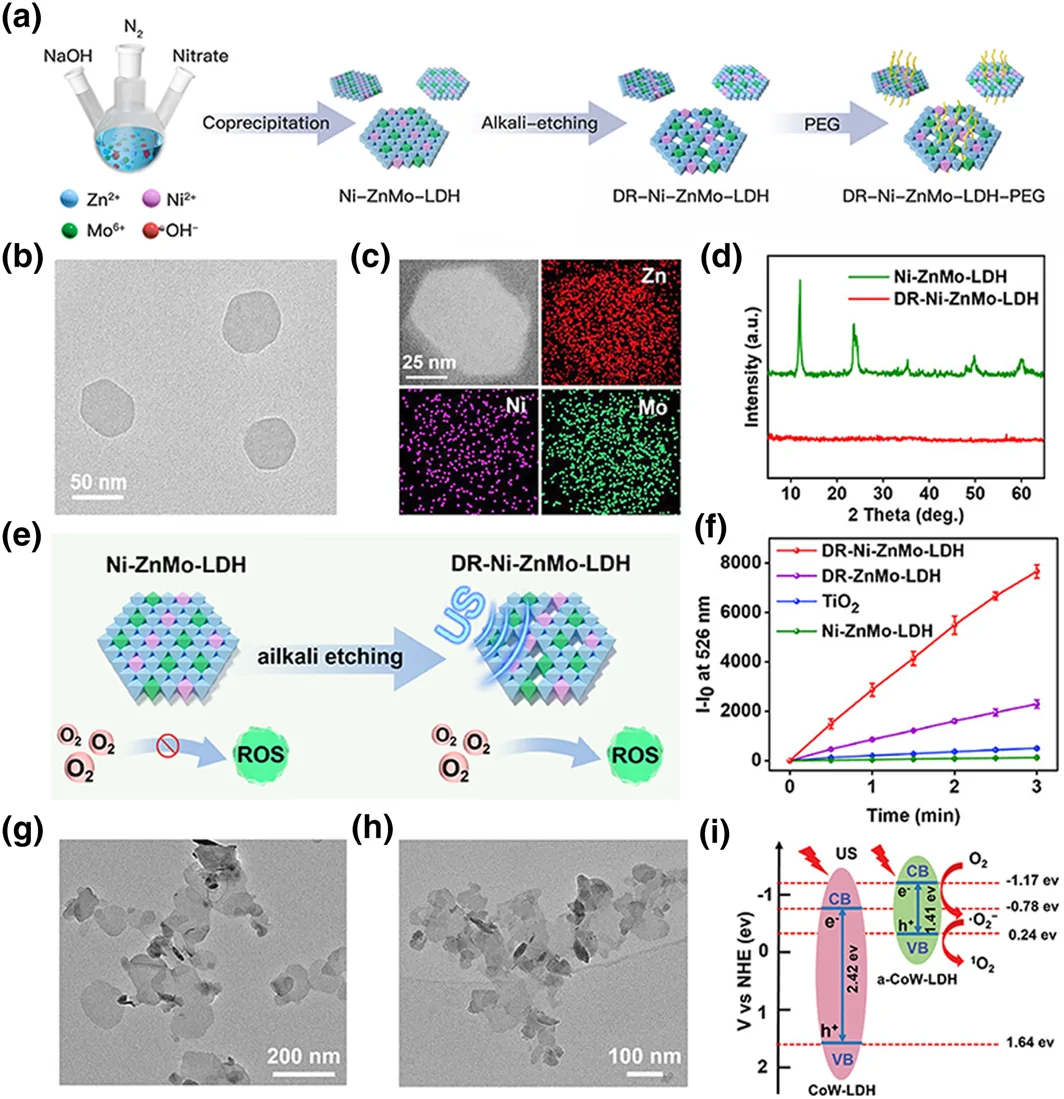
Schematic diagram of water‐thermal method for regulating the structure and properties of Layered double hydroxides (LDHs). (a) Schematic illustration of the preparation of DR‐Ni‐ZnMo‐LDH‐PEG nanosheets. (b), (c) TEM and EDX elemental mapping of Ni‐ZnMo‐LDH nanosheets. (d) XRD patterns of the Ni‐ZnMo‐LDH and DR‐Ni‐ZnMo‐LDH nanosheets. (e) Schematic diagram of ROS generation by US‐activated DR‐Ni‐ZnMo‐LDH sonosensitizer. (f) Relative fluorescence intensity of SOSG at 526 nm after the addition of the TiO_2_, Ni‐ZnMo‐LDH, DR‐ZnMo‐LDH, and DR‐Ni‐ZnMo‐LDH. Data are presented as mean values ± s.d. (*n* = 3). Reproduced with permission.^[58]^ Copyright 2024, Wiley‐VCH. TEM image of (g) CoW‐LDH and (h) a‐CoW‐LDH nanosheets. (i) Band diagrams of the CoW‐LDH and a‐CoW‐LDH nanosheets. Reproduced with permission.^[59]^ Copyright 2026, Wiley‐VCH.

Deng et al. successfully synthesized MgAl‐Cl‐LDH nanoparticles via a hydrothermal method.^[56]^ The mixed MgCl_2_/AlCl_3_ solution was quickly added to NaOH, stirred for 10 min, and then transferred to a Teflon‐lined autoclave and hydrothermally treated at 100°C for 16 h. Cyril Aymonier employed the hydrothermal method to rapidly synthesize LDHs with different metal compositions.^[57]^ As shown in Figure 3a–f, Wang's team synthesized LDH nanosheets of approximately 150 nm loaded with the anticancer drug simvastatin for the treatment of breast cancer.^[58]^ Yang et al. etched the Ni‐ZnMo‐LDH synthesized via the hydrothermal method to achieve ultrasound‐activated sono‐immunotherapy.^[59]^ Tan et al. prepared CoW‐LDH samples via a straightforward hydrothermal method, yielding two‐dimensional nanosheets with dimensions of 80–150 nm (Figure 3g–i).^[60]^ Subsequent acid etching revealed that the resulting amorphous CoW‐LDH nanosheets exhibited significantly enhanced ROS generation activity under ultrasonic irradiation, demonstrating approximately 17‐fold higher activity compared to commercial TiO_2_ photosensitizers. Zhang enhanced the efficacy of photothermal immunotherapy by loading PLD onto hydrothermally synthesized LDH and hybridizing it with Lst, enabling dual modulation of ECM degradation and ROS scavenging in solid tumors.^[61]^

#### 2.1.3 Homogeneous alkalinization method

Homogeneous alkalinization typically generally denotes a process that achieves gradual precipitation of metal ions by steadily releasing OH^−^ to regulate pH value, resulting in the formation of LDHs with high crystallinity and homogeneous composition. In contrast to conventional coprecipitation, this method prevents localized pH spikes caused by direct alkali addition, thereby avoiding instantaneous supersaturation of metal ions and the formation of amorphous or impure precipitates. When urea is employed as the OH^−^ source, its decomposition autonomously supplies carbonate anions during the reaction, simplifying the synthesis of carbonate‐intercalated LDHs. Moreover, the slow decomposition kinetics of urea or hexamethylenetetramine maintains a low initial OH^−^ concentration that promotes uniform nucleation, while sustained OH^−^ release in later stages facilitates the growth of large, low‐defect crystalline grains.

**FIGURE 4:**
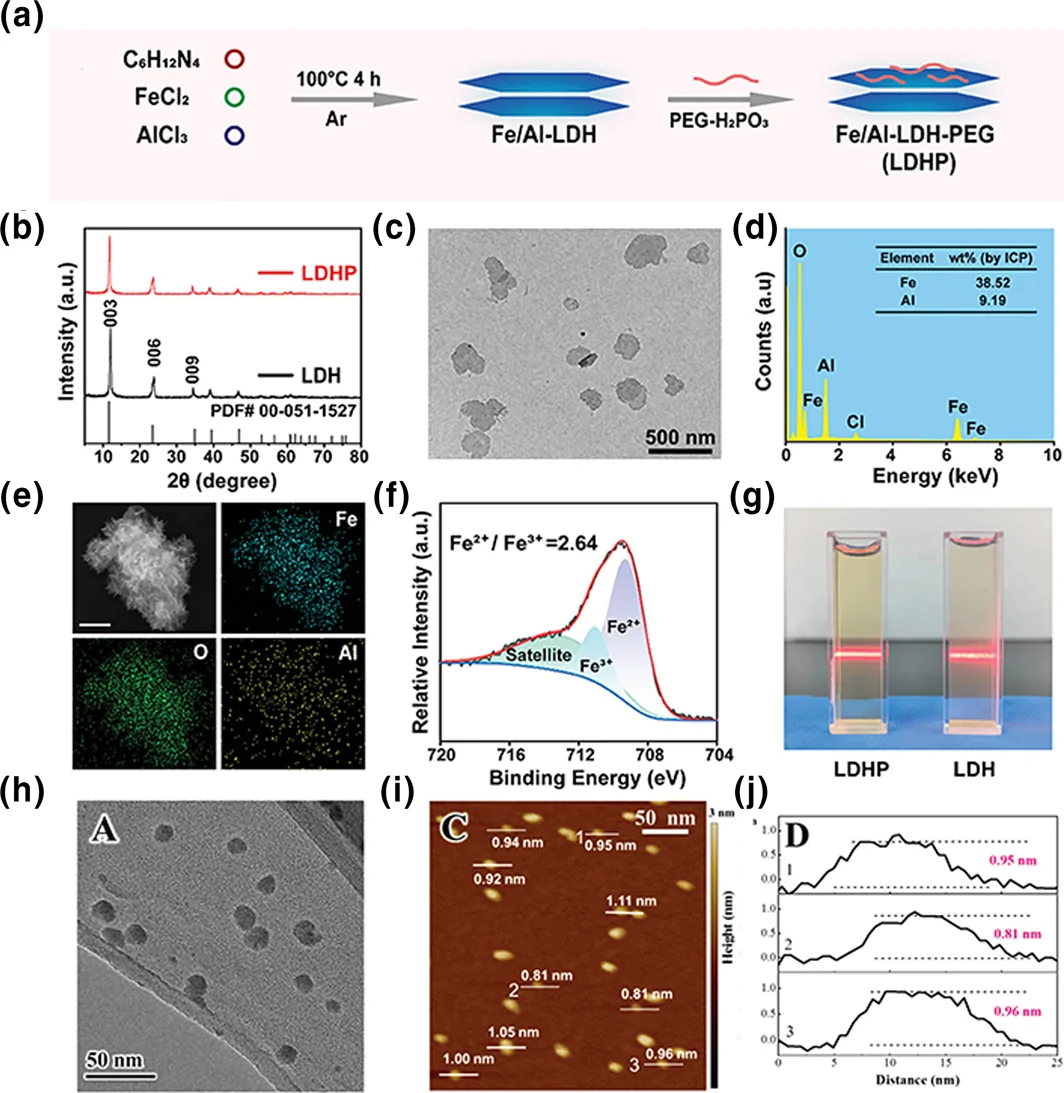
Schematic diagram of the structural regulation of Layered double hydroxides (LDHs) by the microemulsion method. (a) Schematic diagram of Fe/Al‐LDH synthesis and modification. (b) XRD patterns of LDH and LDHP. (c) TEM image, (d) EDS analysis, and (e) corresponding elemental mapping of LDHP. Scale bar: 2 μm. (f) Local XPS spectra for the elementary analyses of iron in LDHP nanosheets. (g) Digital photos of LDH and LDHP in PBS. Reproduced with permission.^[63]^ Copyright 2024, Wiley‐VCH. (h) TEM image of monolayer NiTi‐LDH nanosheets, (i) AFM image and (j) the corresponding height profiles. Reproduced with permission.^[65]^ Copyright 2015, Royal Society of Chemistry.

Ulla Gro Nielsen et al. employed homogeneous alkalinization to synthesize a series of ZnAl‐LDH materials under varying reaction times (7, 12, 16, and 24 h).^[62]^ The results revealed a significant influence of reaction duration on the phase composition: only Al(OH)_3_ precipitate formed at 7 h, while ZnAl‐LDH structures began to emerge only after extending the reaction time to 8 h. This finding demonstrates the existence of a critical reaction time window for ZnAl‐LDH formation, providing key insights into its nucleation and growth mechanisms. As shown in Figure 4a–g, Shi optimized this method by introducing an argon atmosphere during synthesis. Specifically, a mixed solution of AlCl_3_·6H_2_O, FeCl_2_, and hexamethylenetetramine (HMT) was heated at 100°C for 4 h under a nitrogen atmosphere, followed by modification with phosphate‐terminated PEG to successfully prepare 300 nm‐sized AlFe‐LDH‐PEG.^[63]^ Due to the hydrolysis characteristics of Cr^3+^/Ni^2+^ ions, synthesizing NiCr‐LDH requires stringent conditions. To address this, Matías Jobbágy integrated a microwave reactor into the urea‐mediated homogeneous alkalinization process. The microwave irradiation enabled rapid heating with minimal temperature gradients, allowing the precipitation driving force to increase rapidly and uniformly. This approach overcomes the limitations of conventional coprecipitation, which demands prolonged reaction times and elevated temperatures for NiCr‐LDH synthesis.^[64]^

#### 2.1.4 Microemulsion method

The fundamental principle of synthesizing LDHs via the microemulsion method is to employ a thermodynamically stable microemulsion system, which consists of an aqueous phase, an oil phase, surfactants, and co‐surfactants, as the reaction medium. In this approach, aqueous solutions that contain di‐ and trivalent metal ions as well as alkaline precipitating agents are individually encapsulated within nanoscale water cores dispersed in the oil phase. When these microemulsions are mixed, a coprecipitation reaction is initiated, which guides the crystallization of LDHs within the restricted nanodomains of the water cores. This process is fundamentally distinct from conventional coprecipitation because the interfacial confinement effect of microemulsions precisely regulates crystal growth kinetics, resulting in LDH nanoparticles that have narrow size distribution (typically 20–100 nm), uniform morphology (e.g., platelet or spherical), and excellent dispersibility. Zhang et al. successfully synthesized monolayer NiTi‐LDH nanosheets via an in situ growth approach within a reverse microemulsion system comprising surfactants, cosurfactants, and an aqueous phase (Figure 4h–j).^[65]^ The study revealed that in conventional aqueous systems, the absence of effective spatial confinement promotes spontaneous stacking of LDH layers into multilayered structures, with coprecipitation‐derived products typically exhibiting large particle sizes and thicknesses. In contrast, the nanoscale water cores of the microemulsion system provide a unique confined environment for LDH growth. At the oil‐water interface, surfactant molecules not only effectively shield interlayer interactions but also suppress vertical crystal growth through steric hindrance effects, thereby enabling the controlled synthesis of monolayer NiTi‐LDH nanosheets.

#### 2.1.5 Microwave‐assisted synthesis

As an advanced technique, microwave‐assisted synthesis employs microwave energy to heat reaction systems. Its principle relies on the intense interaction of microwaves with polar molecules/ions, generating rapid and uniform volumetric heating via dielectric loss. This method significantly accelerates reactions, lowers energy consumption, and, due to its selective heating, facilitates more homogeneous nucleation and growth. Wang's team rapidly synthesized MgAl‐LDHs with unique maple‐leaf‐like spherical morphologies via a microwave‐assisted approach in a glycol‐water mixed solvent. A solution containing MgCl_2_·6H_2_O, AlCl_3_·6H_2_O, and NaOH in a 2:1 M ratio was subjected to microwave irradiation at 300 W for 30 minutes. The high dielectric loss of ethylene glycol notably improved microwave absorption efficiency, effectively suppressing particle agglomeration and enabling the successful formation of mesoporous maple‐leaf‐like structure self‐assembled from nanosheets.^[66]^ Similarly, Qiao synthesized porous Zn‐Co‐LDH nanosheets through microwave‐assisted synthesis, which significantly improved the loading capacity of LDHs.^[67]^ Rajamani Nagarajan and colleagues synthesized ternary Li‐M‐Al‐LDHs (M = Mg, Co, Ni, Cu, Zn, or Cd) using a microwave‐assisted method. When the metal nitrates and a specific amount of LiAlH_4_ were placed in a microwave reactor (with a power ranging from 100 to 800 W), the reaction time for the formation of LDHs was as short as 12–15 minutes in the acidic solution.^[68]^

#### 2.1.6 In situ transformation method

**FIGURE 5:**
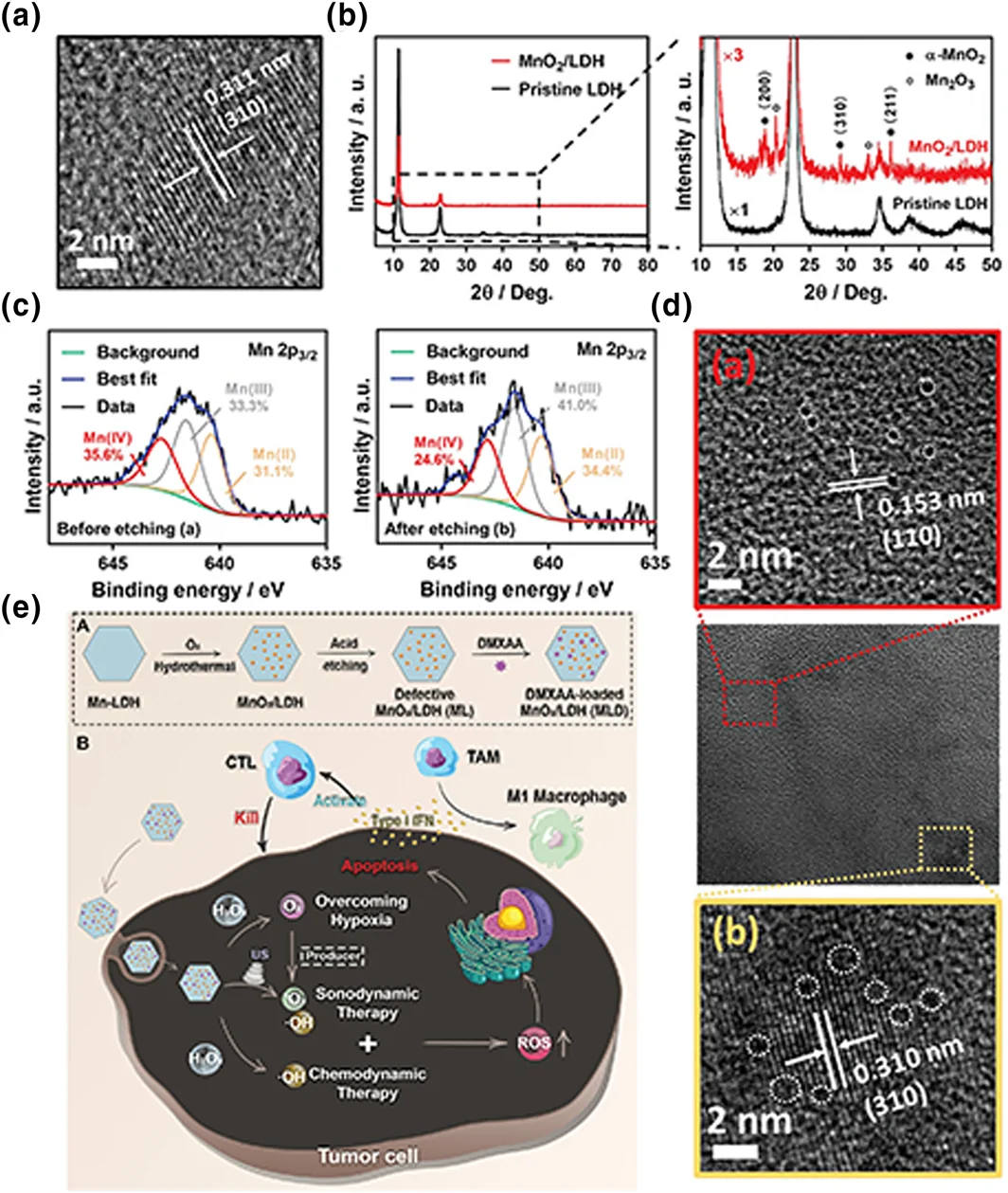
Schematic diagram of in situ transformation method for regulating the structure of Layered double hydroxide (LDH). (a) HRTEM image of the MnO_2_ nanodot on MnO_2_/LDH NPs. (b) XRD patterns of pristine LDH and MnO_2_/LDH powders. (c) High‐resolution XPS spectra of Mn 2p3/2 of MnO_2_/LDH powders before and after acid etching. (d) HRTEM images of ML NPs comprising small MnO_2_ nanodots (yellow) and associated defects, white circles represent the defects. (e) Schematic illustration of the synthetic procedure of MLD NPs and their self‐enhanced SDT and CDT effect. Reproduced with permission.^[72]^ Copyright 2023, Wiley‐VCH.

The in situ solid‐phase transformation method is LDH preparation method based on the topological reconstruction mechanism at the solid‐liquid interface. It achieves the direct growth of LDH on the substrate surface through the local dissolution and in situ reconstruction of metal or metal compound precursors. It is characterized by simple operation, short process flow, and high efficiency, eliminating the need for pre‐preparation of high‐purity precursors or complex pretreatment. Meanwhile, through precise regulation of reaction conditions, oriented transformation of precursors to LDHs can be realized. This approach reduces side reactions, ensures high raw material utilization efficiency, and minimizes the introduction of external impurities, thereby resulting in better product purity and phase uniformity. Furthermore, it is highly suitable for the preparation of LDH‐based composites. This is because it enables LDHs to establish strong interfacial interactions with the matrix, avoiding issues of particle agglomeration or uneven dispersion encountered in traditional physical mixing methods, and consequently improving the performance of the composites. Pan employed porous carbon derived from a Ni‐based metal‐organic framework (Ni‐MOF) as the precursor. By taking advantage of the abundant porous structure of porous carbon to anchor Ni^2+^ generated by the Ni‐based MOF itself and externally added Al^3+^, leading to in situ synthesis of a novel core‐shell structured 2D NiAl‐LDH nanosheet material with a cross‐linked nanosheet structure and high electrical conductivity.^[69]^ Liu's team successfully synthesized NiCoFe‐LDH with a hollow structure (HP) using a two‐step self‐template in situ conversion strategy. In this process, MOFs were first etched to form a hollow structure, followed by the in situ growth of LDHs on their surface, achieving the integrated construction of hollowing and LDHs formation.^[70]^ Wang's team utilized liquid‐phase transmission electron microscopy (liquid‐phase TEM) to observe the in situ transformation process of metal‐organic framework (MOF) nanoparticles into hollow LDHs nanocages in real‐time.^[71]^ As shown in Figure 5a–e, Xu's team synthesized manganese ion‐doped LDHs nanoparticles via an in situ method. Subsequently, they created structural defects through acid etching and loaded the surface with the vascular disrupting agent DMXAA, successfully constructing a multifunctional sonosensitizer.^[72]^

Although the in situ solid‐phase transformation method has significant advantages in constructing core‐shell and porous structures of LDHs, it still has some limitations. Firstly, this method usually relies on the preparation of precursors, and the synthesis process is complex and lengthy. Secondly, during the transformation process, it is affected by the solid‐liquid interface diffusion and local dissolution‐reconstruction mechanisms, which can easily lead to uneven component composition. Moreover, the construction of porous structures often has the problem of decreased mechanical properties, which may cause drugs to be released before reaching the target site in the drug delivery field. Finally, the in situ solid‐phase transformation method involves the coupling of multiple reaction mechanisms, and the dynamic evolution process is not yet fully clear. Therefore, in the future, we should focus on how to achieve precise structural control, improve the stability of LDHs, and clarify the mechanism of action.

#### 2.1.7 Other synthetic methods

While coprecipitation and hydrothermal methods are widely employed for the bottom‐up synthesis of LDHs, researchers have also developed a range of alternative synthetic strategies‐such as ball milling, electrodeposition, separated nucleation and crystallization, and atom economy approaches‐to achieve precise control over the morphology, size, and composition of the resulting materials. For instance, Li and colleagues synthesized highly active MgAl‐LDH catalysts via a ball milling method, in which a mixture of Mg(NO_3_)_2_·6H_2_O, Al(NO_3_)_3_·9H_2_O, NaOH, and Na_2_CO_3_ was milled with zirconia balls in a grinding jar at 1200 rpm for 1 hour.^[73]^ This mechanochemical route leverages a solid‐state reaction mechanism driven by mechanical force, enabling efficient conversion of mechanical energy into chemical energy while circumventing the need for precise pH control or elevated temperature conditions typically required in conventional coprecipitation. Notably, this approach is not only operationally simple and rapid (requiring only 1 h) but also avoids the use of organic solvents, resulting in a 23‐fold enhancement in production efficiency compared to traditional methods. It thus offers a green and efficient pathway for synthesizing LDH‐based catalysts.

In another study, Zheng adopted a two‐step strategy involving electrodeposition followed by anion exchange to fabricate Ca‐based LDHs.^[74]^ The electrodeposition step was performed using a porous carbon substrate as the cathode and an aqueous solution containing 0.3 M Ca(NO_3_)_2_ and 0.1 M Al(NO_3_)_3_ as the electrolyte. By carefully modulating the current density (ranging from 20 to 110 mA/cm^2^), the local pH and OH^−^ generation rate was controlled, allowing tailored growth of LDHs with specific morphologies, such as vertically aligned nanosheets, while minimizing the formation of impurity phases like Al(OH)_3_ or Ca(OH)_2_. Phase‐pure CaAl‐LDHs were successfully obtained at a current density of 35 mA/cm^2^. Similarly, Yu utilized an electrodeposition method to grow CoFe‐LDH directly on nickel foam and subsequently deposited Pt nanoparticles uniformly on the LDH surface using the same technique.^[75]^ Zhao's team applied a separated nucleation and crystallization strategy, in which the rotation speed of a colloid mill reactor and the concentration of salt solutions were systematically optimized.^[76]^ With the addition of a small amount of a crystal growth inhibitor (formamide), they developed a scalable and straightforward method for synthesizing monolayer LDH nanosheets. This approach yielded a series of monolayer MgAl‐, CoAl‐, NiCo‐, and NiFe‐LDH nanosheets with a uniform thickness of approximately 1 nm. Additionally, Lei's group employed an atom economy method using CaO, Al(OH)_3_, and Ca_2_SO_4_ as raw materials to prepare SO_4_^2‐^‐intercalated CaAl‐LDH for the rapid removal of heavy metals from contaminated soil.^[77]^

### 2.2 Top‐down

#### 2.2.1 Liquid‐phase exfoliation method

The liquid‐phase exfoliation method achieves lamellar dissociation by dispersing bulk LDHs in highly polar solvents (such as water, formamide, and isopropanol) and leveraging the interlayer swelling effect induced by solvent molecule penetration. Its mechanism of action involves solvent molecules penetrating the interlayer domain of LDHs, weakening the electrostatic attraction between the layers and interlayer anions, causing the interlayer spacing to swell, and triggering progressive exfoliation of the layers from the crystal edge to the center. Eventually, a colloidal dispersion of single‐ or few‐layer LDH nanosheets is acquired. Given that LDH layers carry positive charges and interlayer anions carry negative charges, the electrostatic attraction formed between them easily leads to lamellar stacking. Moreover, alterations in synthesis parameters such as pH value, temperature, and aging time can result in excessively high crystallinity of LDHs and oversized crystal growth, increasing the stacking tendency. To synthesize single‐layer LDHs, Zhang et al. first synthesized MgMn‐LDH via the oxidation co‐precipitation method and then dispersed bulk MgAl‐LDH materials in polar solvents.^[78]^ Using the liquid‐phase exfoliation method combined with ultrasonic treatment, they disrupted the interlayer van der Waals forces, while solvent molecules intercalated between layers to achieve nanosheet exfoliation, ultimately obtaining a suspension of single‐layer LDH nanosheets. These nanosheets subsequently self‐assembled with negatively charged silk fibroin through electrostatic interactions to form a uniformly dispersed composite precursor solution, providing a structural basis for constructing injectable hydrogels. *Sun* et al. prepared LDH single‐layer nanosheets in a one‐step process in the presence of formamide.^[79]^ From the molecular mechanism perspective, formamide molecules can form a hydrogen bond network with the hydroxyl groups on the surface of the LDH layer plates, effectively overcoming the electrostatic attraction and van der Waals forces between the layers, thereby triggering the spontaneous exfoliation of the nanosheets. Furthermore, Sun achieved precise control over the exfoliation process by establishing a quantitative correlation between the charge density of the LDH layer and the exfoliation efficiency. Similarly, Chen utilized the intercalation of organic molecules to successfully separate LDH into ultrathin nanosheets with excellent water solubility in formamide.^[80]^ This research successfully achieved the exfoliation and surface functionalization of LDH, and prepared an ultrathin LDH nanosheet with excellent water solubility and biocompatibility. It broke through the bottleneck of traditional LDH materials that are difficult to stably disperse in the body and have poor imaging effects. In the application of tumor treatment, these ultrathin single‐layer nanosheets not only maintain the integrity of the two‐dimensional material's lattice but also expose a larger specific surface area and abundant active sites. This structural advantage has laid the foundation for the loading and targeted delivery of anti‐tumor drugs.

#### 2.2.2 Ion exchange‐assisted exfoliation

The ion‐exchange‐assisted exfoliation method is a preparation approach that achieves efficient exfoliation of LDHs by weakening interlayer forces through ion exchange reactions. Typically using anion‐intercalated LDH precursors (intercalated with anions such as nitrate or carbonate) as raw materials, this method introduces organic anions with large volume and low charge density into specific solvent systems. These organic anions displace the original interlayer anions via ion exchange reactions, thereby significantly expanding the interlayer spacing and reducing the interlayer electrostatic attraction. Under ultrasound‐assisted or mild stirring conditions, the interlayer‐supported organic anions can further disrupt the van der Waals forces and hydrogen bond networks between layers, promoting the swelling of LDHs and their eventual dissociation into monolayer or multilayer nanosheets. This method is characterized by the ability to achieve a controllable exfoliation degree through precise regulation of ion exchange efficiency. The obtained LDH nanosheets exhibit high crystalline integrity and regular 2D morphology, while surface‐residual organic anions enhance their dispersion stability in organic media. However, this method is relatively sensitive to the type of interlayer anions in the precursors and exchange kinetics; some strongly bonded anions (e.g., carbonate) require pre‐displacement via acid treatment or high‐temperature calcination, and the introduction of excessive organic anions may affect the subsequent functional applications of the materials. By optimizing the synergy between ion exchange and exfoliation, this method provides an important technical pathway for the preparation of high‐quality LDH nanosheets. However, for strong‐binding anions such as carbonate ions, direct exchange is impossible. Usually, additional pre‐treatment steps are required, and during the exchange process, it is limited by interface dynamics, which may lead to incomplete exchange. The introduced organic anions, while improving the dispersion of LDHs, may also cover some catalytic active sites, thereby reducing the performance of the material. Therefore, the key challenge for the further development of this method lies in achieving efficient separation while reducing organic residues, improving structural homogeneity and system stability.

#### 2.2.3 Mechanical exfoliation

Mechanical exfoliation uses high‐energy ball milling or grinding to apply shear stress and interlayer impact to LDHs. When mechanical energy exceeds the combined electrostatic and van der Waals binding energy between LDHs layers (> 50 kJ/mol), interlayer bonds break, enabling controlled delamination into ultrathin nanosheets. Lei developed an efficient and environmentally friendly solid‐phase exfoliation method to prepare various LDHs nanosheets,^[81]^ specifically using sucrose and ammonium chloride as grinding media for exfoliation via ball milling technology at room temperature. During ball milling, high‐energy impacts and shear forces overcome interlayer electrostatic interactions and van der Waals forces, successfully exfoliating bulk LDHs into nanosheets with a thickness of 1–3 nm. This method not only avoids the use of toxic organic solvents in traditional liquid‐phase exfoliation but also introduces ‐OH and ‐NH_2_ functional groups (derived from sucrose and ammonium chloride) on the nanosheet surface through the decomposition of grinding media, improving their water dispersibility and stability. Furthermore, lattice defects generated during ball milling increase active sites, significantly enhancing the catalytic performance of the material.

**FIGURE 6:**
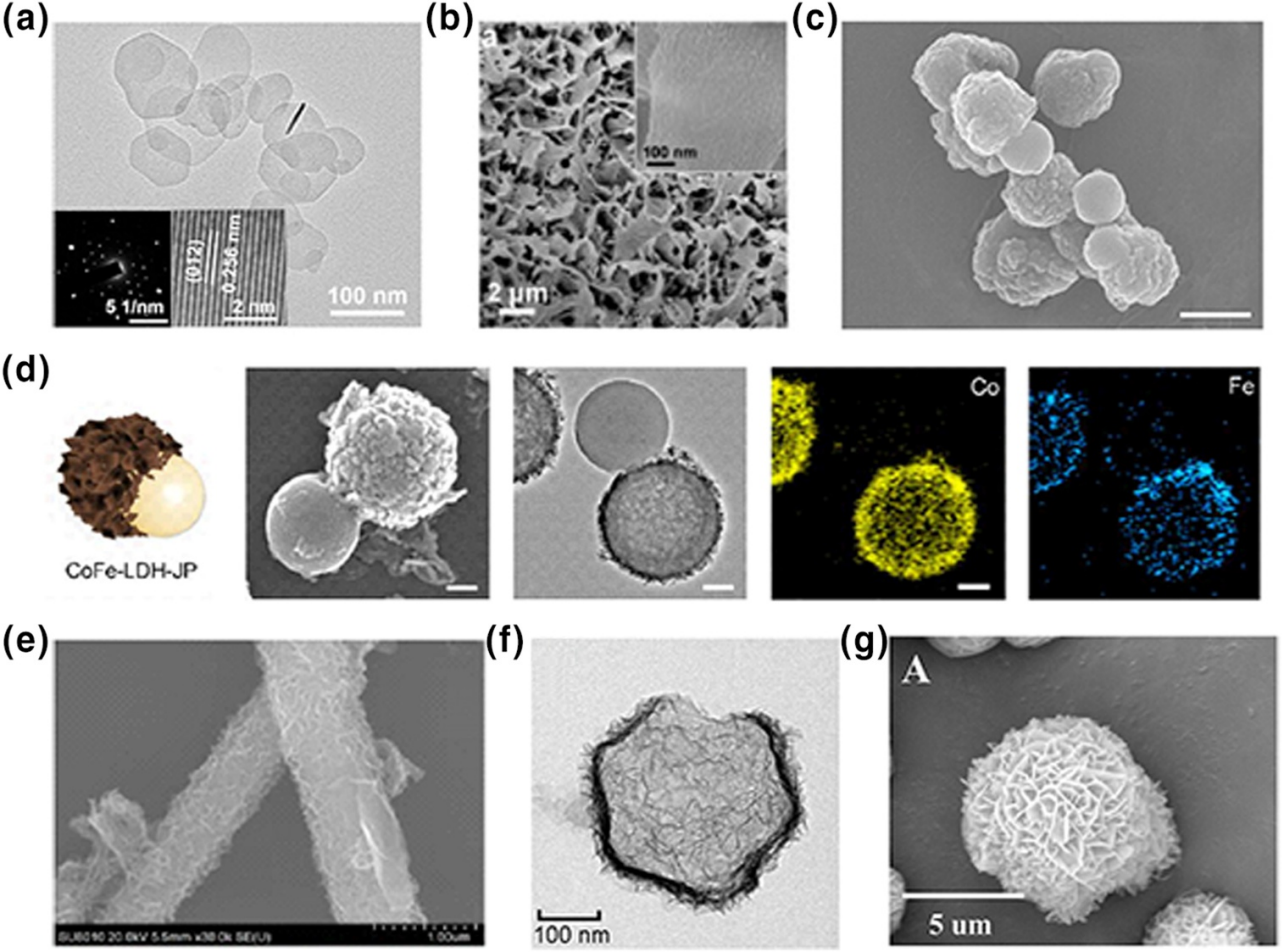
Different structures of Layered double hydroxides (LDHs) and their nanocomposites prepared using various synthetic methods. (a) TEM images of ZnAl‐LDH@I‐IPA synthesized via the co‐precipitation method. Reproduced with permission.^[82]^ Copyright 2026, Wiley‐VCH. (b) SEM images of EE‐NiFe‐LDH. Reproduced with permission.^[83]^ Copyright 2021, Elsevier. (c) The TEM images of MgAl‐LDH‐JPs were fabricated using the swelling‐induced symmetry breaking method. (d) Schematic illustration of CoFe‐LDH‐JP and SEM, TEM, EDS elemental mapping images of CoFe‐LDH‐JPs. Reproduced with permission.^[84]^ Copyright 2026, Wiley‐VCH. (e) SEM images of HHNTs CoVP@NiFeV‐LDH. Reproduced with permission.^[85]^ Copyright 2020, Elsevier. (f) TEM images of NiCo‐LDH/ZHPC synthesized in situ on porous carbon (ZHPC). Reproduced with permission.^[86]^ Copyright 2025, American Chemical Society. (g) SEM images of NiAl‐LDHs. Reproduced with permission.^[87]^ Copyright 2022, American Chemical Society.

In conclusion, researchers have achieved precise control over the morphology of LDHs materials through various strategies, ranging from two‐dimensional nanosheets to three‐dimensional hierarchical structures and even heterogeneous structures (Figure 6a–g). These methods not only expand the structural dimensions of LDHs materials at the morphological level, but more importantly, by means of confinement effects, interface asymmetry, defect engineering, and heterostructure coupling, they effectively regulate the electronic structure and interface reaction behavior of the materials, thereby significantly enhancing their performance in biotherapy, electrocatalysis, and energy conversion fields.[^80^, ^81^, ^82^, ^83^, ^84^, ^85^, ^86^, ^87^]

However, the existing synthesis strategies still encounter some common problems. For instance, the accuracy of structure controllability in different methods remains limited, and the formation mechanism of complex multi‐layer structures is still unclear. Moreover, some methods still face challenges in large‐scale production and structural stability. Therefore, developing simpler, more scalable, and more controllable synthesis methods and establishing quantitative relationships between morphology, defects, and performance remain important research directions in the future.

### 2.3 Structural engineering strategies

The structural engineering of LDHs primarily relies on three core strategies: creating lattice defects by introducing oxygen vacancies, doping with transition metal elements, and reconstructing the morphology and interlayer structure. These strategies enable the precise modulation of the material's electronic band structure, active sites, and specific surface area at the atomic and nanoscale levels, which consequently comprehensively enhances its catalytic activity, drug‐loading capacity, and beyond.

For instance, Zhang precisely reconstructed the active sites of LDH by removing the inert zinc and aluminum components to alter the atomic environment of the transition metals, thereby optimizing the electron occupancy of the d orbitals at the metal center.^[88]^ Through the synergistic effect of multiple metal components, a highly disordered local environment was constructed, breaking the performance bottleneck of traditional LDH catalysts. While Yu's team introduced oxygen vacancies into CoFe‐LDH, they utilized defect engineering to restructure the electronic structure and optimize the adsorption energy of the reactants, significantly enhancing its catalytic activity similar to that of an enzyme. Furthermore, on this basis, by integrating smart phone terminals, a low‐cost and highly efficient multi‐biomarker detection platform has been constructed, providing a new technical solution for immediate medical diagnosis.^[89]^ Not only that, the incorporation of transition metals can also enhance the catalytic performance of LDH materials. Sun et al. systematically quantified the significant role of iron sites in enhancing the catalytic activity of LDH.^[90]^ By establishing a volcano‐type relationship between iron content and catalytic performance, the underlying mechanism of concentration regulation in doping was revealed. In terms of metal doping and synergistic effects, this study clarified the differences in catalytic activity after different matrix metals (Ni, Co, Mn, Cu) combined with iron, and found that NiFe‐LDH exhibited the highest catalytic activity. More importantly, it was discovered that there is an optimal performance threshold for iron content at approximately 20%. Excessive iron doping, on the other hand, will inhibit the catalytic activity. This is mainly attributed to the fact that when the Ni matrix is combined with approximately 20% of Fe, it possesses the most ideal electronic structure, thereby maximizing the conversion rate of active sites on the catalyst surface. This provides an important exploration for achieving precise regulation of the active sites of LDH nanozymes in the field of biomedicine. In addition, through the use of LDH for topological conversion, Ji's team obtained ZnAl‐TCPP nanosheets, and the ROS generation activity achieved a significant improvement of over 3.0 times compared to free TCPP.^[91]^ This improvement in the catalytic performance is mainly attributed to the electronic regulation and defect formation that occur during the phase transition process. Firstly, the reorganization of the material structure led to a reduction in the band gap, making the charge transition under light/phonon excitation more efficient. Secondly, during the phase transition process, abundant unsaturated metal sites were inevitably generated, and these defects greatly inhibited the recombination of excited‐state electron‐hole pairs. Finally, the strong heterometallic center coupling effect in the metal nodes deeply modulated the electron cloud density of the surrounding TCPP ligands. Through this regulation, not only was the common aggregation‐induced quenching effect of free TCPP effectively overcome, but also the sensitization ability and intermolecular energy transfer efficiency of the porphyrin ligand were significantly enhanced. Eventually, a large amount of ROS was generated. Xu's team directly assembled LDHs onto spherical templates to form hollow nanospheres with a high specific surface area and mesoporous structure. After forming the hollow shell, they calcined it at 500°C to convert it into layered double oxide. Subsequently, in a solution containing anionic drugs, the unique memory effect of LDH was utilized to induce structural reconstruction restoring the layered structure. During this reconstruction process, drug molecules were spontaneously and firmly immobilized within the interlayer space. This approach achieved an extremely high drug encapsulation efficiency and laid the foundation for subsequent targeted release.^[92]^

## 3 LDHS FOR DISEASE THERAPY

### 3.1 Controlled release of drugs

The clinical application of chemotherapeutic drugs has long been limited by core drawbacks. Due to its lack of targeting ability, it will lead to serious toxic side effects and cause complications such as neutropenia and mucositis. Notably, 15% of patients treated with anthracycline drugs face the risk of life‐threatening infections. In addition, chemotherapy drugs have poor stability and are prone to degradation due to the influence of enzymes in the body, pH fluctuations, and other factors, which can lead to the development of drug resistance in tumor cells and result in poor treatment outcomes. Moreover, administering high doses will further increase the toxicity to organs.

The unique structure of LDHs, an inorganic metallic material, renders them ideal drug carriers with remarkable advantages. Their positively charged laminates enable efficient loading of anionic chemotherapeutic drugs (e.g., 5‐fluorouracil, doxorubicin) through electrostatic interactions. Leveraging the enhanced permeability and retention (EPR) effect of tumor tissues, LDHs achieve passive targeted accumulation and release drugs in response to the weakly acidic microenvironment of tumor cells thereby avoiding damage to normal tissues and significantly reducing side effects such as myelosuppression and organ toxicity. The interlayer structure of LDHs protects drugs from enzymatic hydrolysis and environmental influences, prolonging the drug half‐life and effectively improving drug stability and bioavailability. Furthermore, by regulating the composition of metal ions in LDHs, synergistic effects of magnetic resonance imaging, photothermal therapy, and chemotherapy can be achieved, constructing an “integrated diagnosis and treatment” platform to further enhance the precision and efficacy of tumor therapy.[^93^, ^94^, ^95^] Table 1 lists the drug delivery strategies for different LDHs and makes a comparison.

**TABLE 1:** Representative examples of distinct Layered double hydroxide (LDH) drug loading strategies along with a comparative analysis of their respective advantages.

Drug	Drug‐loading strategy	Advantage	Reference
BBR	Anion exchange	Solubility increased	[96]
DOX	Electrostatic adsorption	High drug payload	[98]
AFGd‐LDH	Interlayer intercalation	Excellent accumulation	[99]
EDTA	Anion exchange	Disrupt intercellular adhesion in tumors	[100]
LRT	Interlayer intercalation	Induce immunological memory	[101]
LRsBAR	Interlayer intercalation	Dual‐targeted therapy	[102]

**FIGURE 7:**
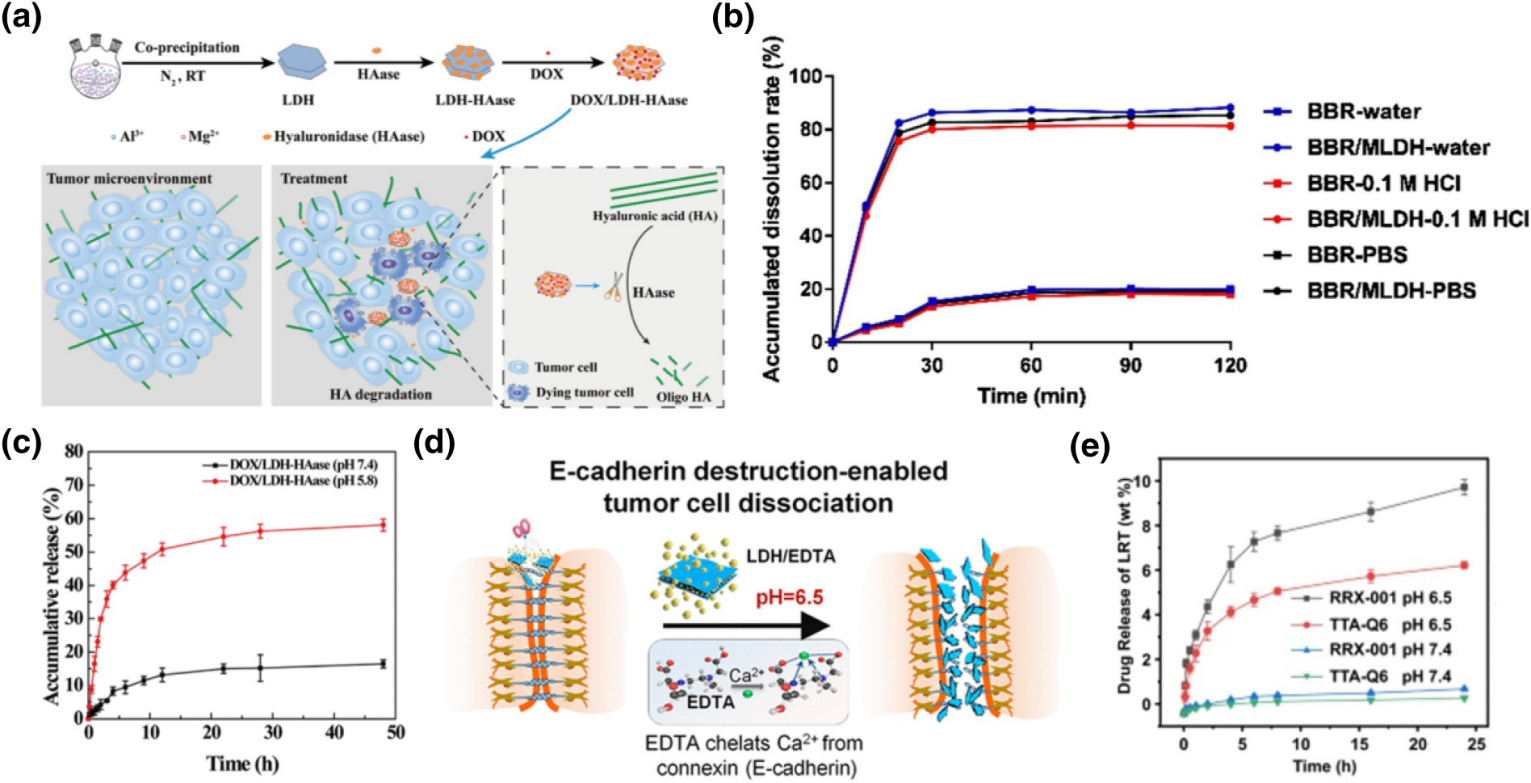
LDH‐based nanomaterials for controlled release of drugs. (a) Schematic diagram of the synthesis of DOX/LDH‐HAase nanosheets and its therapeutic mechanism Reproduced with permission.^[98]^ Copyright 2021, Science China Press. (b) The release profile of the drug over time in different environments. Reproduced with permission.^[96]^ Copyright 2020, Elsevier. (c) The release profile of DOX/Layered double hydroxide (LDH)/HAase under different pH conditions. Reproduced with permission.^[98]^ Copyright 2021, Science China Press. (d) Schematic diagram of the synthesis of LDH/EDTA nanosheets and its therapeutic mechanism. Reproduced with permission.^[100]^ Copyright 2020, American Chemical Society. (e) The drug release profiles of different groups over time at pH 6.5 and 7.4. Reproduced with permission.^[101]^ Copyright 2023, Nature Publishing Group.

Based on the above advantages, researchers have developed a series of drug delivery systems based on LDHs. The Yan team developed berberine/monolayer LDH (BBR/MLDH) nanocomposites, aiming to address the issue of low oral bioavailability of poorly soluble drugs.^[96]^ Berberine, as a natural alkaloid, has multiple functions such as anti‐tumor and anti‐inflammatory effects, but its application is limited due to poor solubility and rapid metabolism in the body. By loading BBR onto MLDH, the solubility and dissolution rate of berberine were significantly enhanced. Pharmacokinetic studies in rat models demonstrated marked improvements in key parameters, including increased area under the curve and peak plasma concentration (*C*_max_) (Figure 7b). Although this system did not introduce active targeting and responsive release strategies, it enhanced the feasibility of using LDH as a drug carrier to improve bioavailability.

By taking advantage of the exchangeability of interlayer anions in LDH, the dopamine‐coated layered double hydroxide (PDA@LDH) loaded with chemotherapeutic drugs not only exhibits excellent biocompatibility but also effectively prevents drug leakage. Furthermore, various biomolecules such as DNA and RNA can be intercalated into LDHs and released in a controlled and sustained manner, enabling precise delivery of these biologically significant molecules.^[97]^ As shown in Figure 7a Shi et al. modified hyaluronidase (HAase) on the surface of LDH and loaded DOX to construct the LDH‐HAase‐DOX treatment system.^[98]^ By using HAase to degrade the excessive hyaluronic acid (HA) expressed in the tumor microenvironment, the drug can penetrate and accumulate more effectively at the tumor site. At the same time, by leveraging the LDH pH‐responsive release feature, passive targeting and active targeting are synergistically achieved, significantly enhancing the anti‐tumor efficacy. Experimental results demonstrated that the as‐prepared DOX/LDH‐HAase composite achieved a high drug loading capacity of 34.2% and exhibited excellent colloidal stability in solution (maintaining stable dispersion for over 72 hours) (Figure 7c). However, in the complex environment within the body, LDH is prone to form protein caps with plasma proteins, which leads to its rapid clearance by the mononuclear phagocytic system and a shortened circulation time. Additionally, the long‐term stability and catalytic efficiency of HAase may further decline.

In addition, Zhou et al. developed a novel nanocomposite by intercalating atorvastatin (ATO), an anti‐inflammatory and anti‐lipid peroxidation drug, along with ferritin heavy chain (FTH) into MgAlGd‐LDH.^[99]^ ATO binds to Gd‐LDH nanosheets through hydrogen bonding and electrostatic interactions. The ‐OH and ‐NH groups on its aromatic ring, along with its active sites, facilitate reactions with ROS, thereby exerting neuroprotective effects. By leveraging the affinity of FTH for transferrin receptor 1 (TfR1) on brain microvascular endothelial cells, the system can cross the blood‐brain barrier via receptor‐mediated endocytosis. This enables AFGd‐LDH to effectively accumulate in ischemic regions of the brain, allowing for both visualized diagnosis and therapeutic intervention. This integrated design‐combining therapy, targeting, and imaging‐also offers broader possibilities for the development of LDH‐based drug delivery platforms. Studies demonstrate that tumor cells establish tight junctions through various cell adhesion proteins, forming a dense extracellular matrix (ECM) network. This structural characteristic not only enhances the mechanical strength of tumor tissue but also creates a physical barrier to drug penetration. Qin's team leveraged the calcium‐dependent nature of cell adhesion proteins to construct a ZnAl‐LDH‐based nano‐platform loaded with the metal chelator EDTA (Figure 7d).^[100]^ Under acidic conditions, this platform releases EDTA, which chelates and depletes calcium ions from tumor cell calreticulin, leading to the dissociation of tumor cells. These dissociated cells are subsequently expelled via defecation without invading healthy tissues, virtually eliminating the possibility of tumor metastasis. This design strategy no longer focuses solely on conventional drugs; instead, it employs physical disruption to break the adhesive junctions between tumor cells, causing them to detach from the primary site and lose their metastatic capability. By fundamentally blocking the process of tumor metastasis, this approach offers a novel strategy for preventing and treating metastasis in advanced‐stage cancers. The TME is composed of immune cells and mesenchymal cells that regulate immune activity. The cellular composition of TME varies significantly across different tumor types, and this heterogeneity leads to diverse immune surveillance and immune response patterns, ultimately influencing the clinical outcomes of immunotherapy. For instance, the lung tumor microenvironment is predominantly characterized by an abundance of immunosuppressive macrophages and DCs, which critically impair the efficacy of immunotherapy by suppressing T‐cell function. To address this challenge, the research team led by Shi utilized LDH as a carrier to co‐deliver a T‐type calcium channel inhibitor (TTA‐Q6) and a CD47 inhibitor (RRX‐001), achieving targeted accumulation of these therapeutic agents at lung tumor sites.^[101]^ Under the acidic tumor microenvironment, TTA‐Q6 is selectively released, where it disrupts cancer cell calcium uptake, induces endoplasmic reticulum stress, and triggers calreticulin translocation to the cell surface (Figure 7e). The exposed calreticulin effectively activates macrophages and promotes DC maturation, thereby enhancing antigen presentation efficiency and ultimately activating anti‐tumor T‐cell immune responses. Simultaneously, RRX‐001 downregulates CD47 expression, effectively blocking the immune evasion mechanism of calreticulin‐positive cancer cells. This approach, which utilizes the host's immune system to achieve sustained killing effects, can effectively inhibit tumor recurrence.

In addition to the aforementioned applications, Zeng et al. expanded the application scope of LDH to the field of neurological diseases, focusing on the challenges faced by small nucleic acid drugs, such as their inability to passively penetrate cell membranes, low systemic bioavailability, susceptibility to nuclease degradation, and rapid renal clearance.^[102]^ His team developed two dual‐target nanomedicines based on LDH nanoparticles, which can simultaneously reduce amyloid‐β (Aβ) and hyperphosphorylated Tau burden while alleviating neuroinflammation. These engineered dual‐target nanomedicines act concurrently on amyloid aggregates and neuroinflammatory pathways, demonstrating superior therapeutic efficacy compared to single‐target treatment strategies.

### 3.2 Cancer therapy

Over recent decades, cancer treatment has achieved groundbreaking advances. Therapeutic strategies now encompass not only conventional approaches such as surgical resection, radiotherapy, and chemotherapy but also novel modalities‐including molecular targeted therapy, immunotherapy, and nanotechnology‐based treatments‐driven by rapid developments in molecular pathology and nanomedicine, enabling precision strikes against malignancies. However, tumor heterogeneity, complex microenvironmental regulation, and dynamic drug resistance mechanisms remain major obstacles to long‐term therapeutic efficacy, fueling the ongoing exploration of innovative treatment strategies.

#### 3.2.1 Chemodynamic therapy

The deep integration of nanomaterials with medicine offers significant opportunities for innovative cancer treatment strategies. Among them, the nanotherapy system based on ROS has attracted much attention due to its unique anti‐tumor effect. Studies have shown that, due to the unique metabolic characteristics of tumor cells (such as the impaired antioxidant defense system and a lower oxidative stress threshold), they are significantly more sensitive to ROS than normal cells. This biological property provides an important theoretical basis for designing new nanotherapy platforms. Current research mainly focuses on developing nanomaterials with efficient ROS generation capabilities and integrating them systematically with various treatment methods. Among these methods, CDT as a new active oxygen‐dependent anti‐cancer strategy has emerged prominently. Its mechanism relies on Fenton or Fenton‐like reactions to catalyze the conversion of endogenous H_2_O_2_ into highly toxic ROS within tumor cells, thereby damaging critical biomacromolecules in cancer cells.^[103]^ Due to the enhanced catalytic efficiency of Fenton reactions under acidic conditions, CDT can specifically target the acidic TME, enabling tumor‐selective treatment while minimizing damage to normal tissues. Moreover, compared to other therapeutic approaches, CDT does not require external devices or stimulation sources to exert its therapeutic effects. This unique characteristic endows CDT with significant advantages in clinical translation and practical applications, demonstrating broad development prospects. Currently, the development of chemical kinetic formulations with abundant active centers, as well as the rational design of catalysts to efficiently convert hydrogen peroxide into highly toxic hydroxyl radicals, has become a research focus. It is worth noting that the weak acidic characteristic of the tumor microenvironment and the alkaline nature of hydrotalcite synergistically work together to provide ideal reaction conditions for enhancing the kinetics of Fenton‐like reactions. But the traditional binary or ternary LDH systems still have problems such as limited catalytic efficiency and insufficient utilization of the tumor microenvironment. Therefore, how to enhance their therapeutic performance through structural design and functional integration is a key issue.

In order to overcome the problem of insufficient endogenous H_2_O_2_ in TME, the researchers introduced a cascade reaction strategy. Gu et al. designed a two‐dimensional layered double hydroxide ‐based cascade catalytic nano‐platform, denoted as ICG/Fe‐LDH@PEG.^[104]^ Under NIR light irradiation, the ICG component not only generates singlet oxygen ^1^O_2_ to directly kill tumor cells but also produces ·O_2_^−^. These ·O_2_^−^ radicals are subsequently converted into H_2_O_2_ via intracellular superoxide dismutase, significantly elevating H_2_O_2_ levels in the TME. The self‐supplied H_2_O_2_ (from ICG) and endogenous H_2_O_2_ are then catalytically decomposed by Fe^2+^ to efficiently produce highly toxic ·OH. However, this strategy relies on external light excitation and has limited penetration ability for deep tissues of tumors, which restricts its application scope.

**FIGURE 8:**
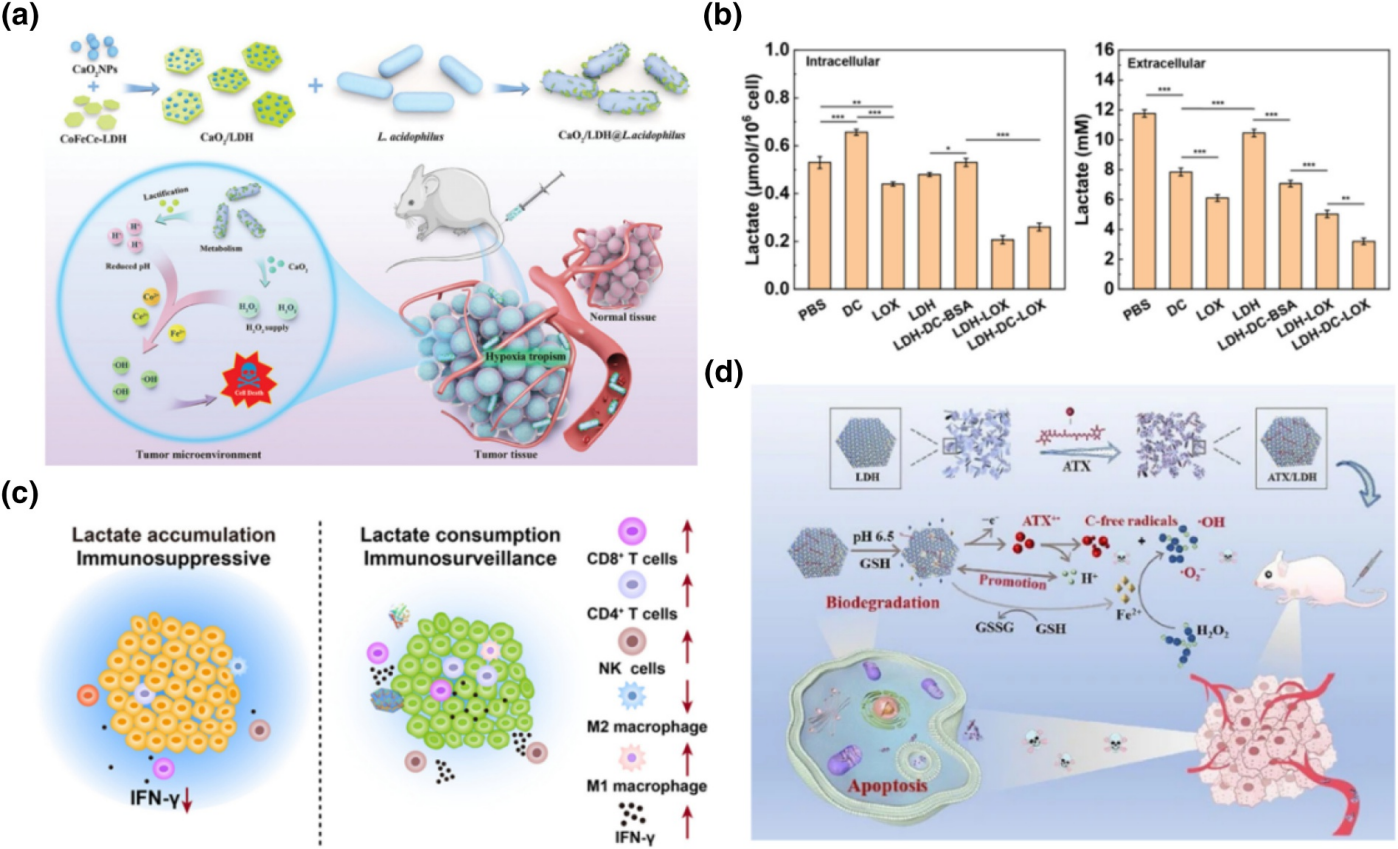
LDH‐based nanomaterials for chemodynamic therapy. (a) Schematic illustration of the therapeutic mechanism of CaO_2_/LDH@Lacidophilus nanosheets. Reproduced with permission.^[105]^ Copyright 2025, Wiley‐VCH. (b) Intracellular and extracellular lactate concentrations in 4T1 cells after different treatments for 12 h (*n* = 3) and (c) Schematic diagram of LDH‐DC‐LOX NPs inducing cascaded chemodynamic/immunotherapy for tumor treatment via modulation of redox metabolism. Reproduced with permission.^[106]^ Copyright 2025, Elsevier. (d) Schematic illustration of the therapeutic mechanism of ATX/Layered double hydroxide (LDH) nanosheets. Reproduced with permission.^[107]^ Copyright 2022, Springer Nature.

Based on this, the researchers attempted to overcome their dependence on external stimuli by regulating the endogenous metabolic remodeling of the tumor microenvironment. Ji et al. utilized *Lactobacillus acidophilus (LA)* and combined it with CoFeCe‐LDH nanosheets loaded with CaO_2_ nanoparticles (Figure 8a).^[105]^ The bacteria selectively accumulate at tumor sites due to their intrinsic tumor‐targeting capability, where their lactate secretion significantly acidifies the TME. This acidic environment not only enhances subsequent Fenton reactions but also triggers the decomposition of CaO_2_ NPs, leading to in situ generation of high‐concentration H_2_O_2_. Furthermore, the polymetallic synergistic centers in CoFeCe‐LDH nanosheets exhibit enhanced Fenton/Fenton‐like activity under acidic conditions, enabling highly efficient conversion of H_2_O_2_ into abundant cytotoxic ·OH radicals. Experimental results demonstrated that this platform achieved a 96.4% tumor suppression rate in murine 4T1 breast cancer models, significantly outperforming conventional CDT agents. Shen et al. developed a Cu^2+^‐doped layered double hydroxide nano‐platform co‐loaded with monocarboxylate transporter inhibitor DC and LOX to dually modulate tumor lactate metabolism and redox homeostasis, thereby potentiating Cu‐mediated CDT and activating antitumor immunity.^[106]^ The resulting LDH‐DC‐LOX nanoparticles exhibit a uniform diameter of 55 nm with excellent stability, enabling efficient cellular uptake by cancer cells and significant tumor‐specific accumulation (Figure 8b–c). Compared with single‐level metabolic regulation, this design is more capable of comprehensively interfering with the tumor metabolic network.

In order to further enhance the catalytic efficiency, the researchers started from the material itself and introduced the effects of multi‐metal synergy and structural distortion. As shown in Figure 8d, Lu's team loaded astaxanthin (ATX) molecules onto the surface of the NiFe‐LDH nanosheets via hydrogen bonding and van der Waals forces.^[107]^ This nanocomposite not only exhibits highly efficient targeting capability but also undergoes degradation within the TME. The released ATX chelates with the active sites on the LDH layers, generating carbon‐centered radicals (ATX•) through a proton‐coupled electron transfer process, thereby exposing a large number of unsaturated active sites. Notably, the Brønsted basic sites on the LDH layers significantly promote proton abstraction, further accelerating the generation of radicals and producing substantial ROS. The combined action of ROS and ATX• induces an intracellular radical stress response, achieving remarkable anti‐tumor therapeutic efficacy. However, the limitation of metal types still hinders the improvement of catalytic performance.

Recent studies reveal that quinary or higher‐order LDHs exhibit exceptional catalytic performance due to lattice distortion effects induced by their diverse elemental compositions. Qiu's team designed and developed a high‐entropy layered double hydroxide nanomaterial by integrating five metal elements (Co, Cu, Fe, Zn, Al) in near‐equimolar ratios into a two‐dimensional LDH structure, creating a multi‐enzyme‐mimicking nano‐platform.^[108]^ In the TME, HE‐LDHs release metal ions to initiate a cascade of enzymatic reactions (including SOD‐like, POD‐like, and GPX‐like activities), enabling sustained ROS generation while depleting GSH‐resulting in superior CDT efficacy. Moreover, the released Zn^2+^ ions activate the cGAS‐STING pathway, promoting the secretion of interferons and pro‐inflammatory cytokines to induce antitumor immune responses, thereby establishing a highly efficient combinatorial cancer treatment strategy.

In addition to regulating the composition, further optimizing the performance through structural derivatives and phase transformation engineering is also a good option. Qiu et al. synthesized CoFe‐LDH materials via a coprecipitation method, followed by calcination treatment at distinct temperatures‐250°C (LDH‐250) and 500°C (LDH‐500)‐to engineer their catalytic properties.^[109]^ This thermal treatment process not only regulates the oxide content of the material but also induces valence state transitions of the metal ions (e.g., Co^2+^/Co^3+^ and Fe^2+^/Fe^3+^), thereby endowing the material with exceptional photothermal conversion capability and highly efficient Fenton‐like catalytic activity. This strategy of precisely tuning the material composition and valence states via calcination temperature provides a novel approach for developing multifunctional nanocatalytic platforms, enabling synergistic photothermal‐enhanced CDT. Despite the potential of LDH‐mediated CDT to respond to theTME, its practical application still faces multiple challenges. Specifically, the transition metal‐mediated Fenton or Fenton‐like reactions in LDHs are highly dependent on low pH and endogenous H_2_O_2_ levels; however, the actual TME is only mildly acidic and local H_2_O_2_ is prone to depletion, which severely limits the catalytic efficiency of LDHs. During in vivo delivery, unmodified LDH possess positively charged surfaces, which readily adsorb blood proteins and undergo aggregation leading to rapid clearance. Moreover, non‐specific leakage of metal ions from the framework during systemic circulation may have potential toxic side effects on normal tissues such as the liver and kidneys. Therefore, on the basis of overcoming complex physiological barriers, establishing a more comprehensive and systematic biosafety evaluation system represents a critical direction for advancing the development of CDT.

#### 3.2.2 Photodynamic therapy

**FIGURE 9:**
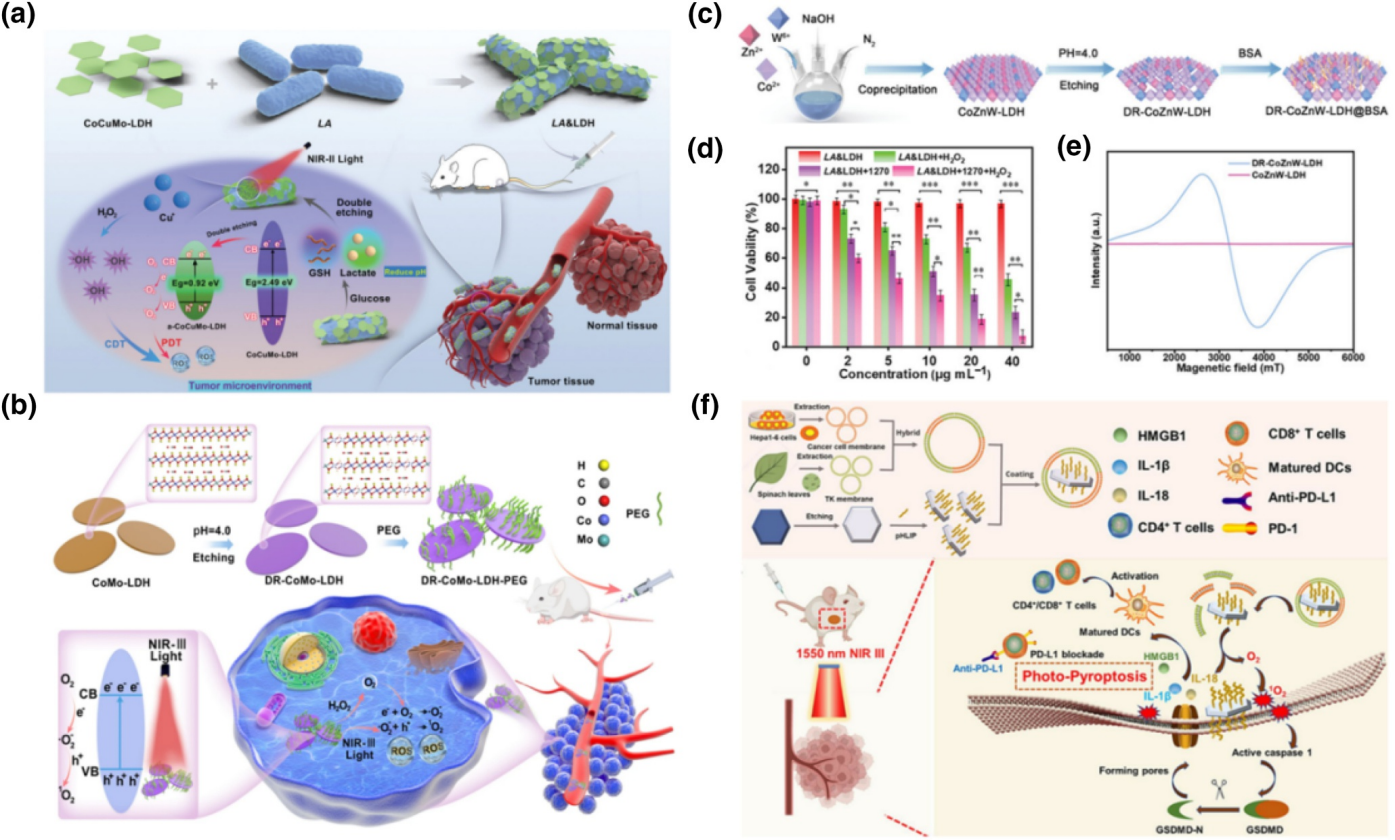
LDH‐based nanomaterials for photodynamic therapy. (a) Schematic illustration of the CoCuMo‐LDH based nanomedicine formation process and its proposed mechanism in cancer treatment. Reproduced with permission.^[113]^ Copyright 2023, Wiley‐VCH. (b) Schematic illustration of the synthesis and antitumor performance of DR‐CoMo‐LDH‐PEG. Reproduced with permission.^[115]^ Copyright 2022, Nature Publishing Group. (c) Schematic diagram illustrating the synthesis mechanism of DR‐CoZnW‐LDH@BSA (d) Cell survival rate under different treatments. (e) Occurrence of oxygen vacancies in Layered double hydroxide (LDH) before and after etching. Reproduced with permission.^[116]^ Copyright 2025, Wiley‐VCH. (f) Synthesis and anti‐tumor performance schematic diagram of membrane‐coated LDH under 1550 nm irradiation. Reproduced with permission.^[117]^ Copyright 2024, Elsevier.

Photodynamic therapy (PDT) is a cancer treatment modality that relies on the synergistic interaction of three key components: a photosensitizer, light (of specific wavelengths), and O_2_. The core mechanism involves photoinduced generation of ROS to selectively destroy tumor cells. Therefore, developing highly active photosensitizers is critical for achieving superior therapeutic outcomes in PDT. The application of photosensitizers in PDT is expanding from traditional organic molecules‐such as porphyrins, porphyrin derivatives, and phthalocyanines‐to the realm of inorganic nanomaterials. These novel photosensitizers demonstrate remarkable advantages due to their unique photophysical properties. Taking TiO_2_ as an example, this classic semiconductor material generates highly oxidative electron‐hole pairs upon ultraviolet (UV) light excitation, and has been successfully applied in skin cancer treatment. However, its clinical utility is limited by poor tissue penetration of UV light, prompting researchers to develop modified variants such as nitrogen‐doped TiO_2_ to extend its visible‐light responsiveness. Notably, LDH nanosheets have also been employed for PDT by either intercalating organic photosensitizers (PSs) within their interlayer spaces or loading PSs onto their surfaces, enabling them to function as active PDT agents. Kun Na's team developed a soluble nano‐system based on chlorin e6 (Ce6) and LDH composites to enhance anticancer PDT.^[110]^ By efficiently loading the hydrophobic photosensitizer Ce6 onto the LDH nanocarrier, the system significantly improves Ce6's water solubility and tumor‐targeting capability, while leveraging LDH's pH‐responsive properties to achieve controlled release of Ce6 in the TME. The composite system demonstrates highly efficient ROS generation capability under laser irradiation, leading to a significant enhancement in PDT efficacy. Furthermore, the LDH carrier not only improves the biocompatibility and stability of Ce6 but also enhances drug accumulation at tumor sites through the EPR effect. Similarly, Lu et al. immobilized the photosensitizer iron(II) phthalocyanine chloride (FePcCl) on the surface of NiCoFe‐LDH through non‐covalent interactions, enhancing the nanomaterial's light absorption capacity and electrical conductivity.^[111]^ In another approach, Wang et al. employed a straightforward method to combine ZnPc with positively charged LDH, effectively addressing the aggregation issue of conventional photosensitizers in biological media.^[112]^ The resulting LDH@ZnPc composite not only exhibits exceptional mitochondrial anchoring capability, significantly boosting ROS generation and mitochondrial oxidative stress, but also activates the GSDMD‐dependent pyroptosis pathway to induce ICD. In clinical practice, PDT has been widely adopted due to its minimally invasive nature, controllability, and high specificity. During PDT, PSs are delivered to the tumor site and activated by external light of a specific wavelength. The excited photosensitizers then transfer energy to surrounding O_2_, generating highly reactive ^1^O_2_, or exchange electrons with substrates to produce ROS such as ·O^2−^ and ·OH, leading to tumor cell ablation. As an externally activated localized therapy, PDT utilizes three non‐toxic components (light, photosensitizer, and oxygen) to generate ROS. This unique mechanism confers two key clinical advantages: (1) exceptional selectivity for tumor tissue, and (2) minimal invasiveness to healthy tissues. Over the past decades, these attributes have established PDT as both a standalone cancer treatment and an effective adjuvant therapy post‐surgical resection to mitigate residual tumor recurrence. As shown in Figure 9a, Weng and colleagues innovatively constructed a TME‐responsive NIR‐II photodynamic therapy platform by bio‐conjugating the probiotic *LA*, which possesses natural tumor‐targeting properties, with CoCuMo‐LDH nanosheets, achieving precise and efficient antitumor therapy.^[113]^ In the TME, the CoCuMo‐LDH nanosheets undergo in situ amorphization transformation induced by the low pH and high glutathione concentration resulting from LA metabolism. Under 1270 nm laser irradiation, the crystal‐to‐amorphous phase transition significantly enhances the photodynamic performance of the material.

However, conventional inorganic NIR‐II photosensitizers exhibit relatively low ROS generation efficiency, which significantly limits their further applications in tumor eradication.^[114]^ Compared to light in the NIR‐I and NIR‐II windows, NIR‐III window light (1350–1870 nm) offers a higher maximum permissible exposure and greater tissue penetration depth, making it a more promising light source for PDT. The teams of Tan and Liang innovatively developed an inorganic photosensitizer system suitable for PDT in the third near‐infrared window (NIR‐III, 1567 nm) (Figure 9b).^[115]^ By subjecting hydrothermally synthesized CoMo‐LDH and NiMo‐LDH nanosheets to acid etching, they successfully constructed defect‐rich layered double hydroxide nanostructures, significantly enhancing the photodynamic performance of the materials. Experimental results demonstrated that under 1567 nm laser irradiation, the ROS generation efficiency of the defect‐engineered CoMo‐LDH nanosheets increased several‐fold compared to the pristine material. The research team further improved the biocompatibility and tumor targeting ability of the nanosheets through polyethylene glycol (PEG) surface modification, enabling efficient induction of cancer cell apoptosis in *in vitro* experiments and exhibiting excellent tumor eradication efficacy in animal models. This breakthrough overcomes the limitations in tissue penetration depth associated with traditional photodynamic therapy. In a related approach, as shown in Figure 9c–e Zhang modified acid‐etched CoZnW‐LDH with bovine serum albumin (BSA).^[116]^ The modified CoZnW‐LDH demonstrated highly efficient ^1^O_2_ generation capacity and exhibited exceptional performance as a photosensitizer for PDT. This strategy of introducing defect structures via acid etching effectively modulates the recombination process of electron‐hole pairs and enhances the charge carrier separation efficiency, providing an important research direction for the design of high‐performance photosensitizing materials.

Yu et al. engineered an intelligent nano‐platform (CTEP) based on acid‐etched layered double hydroxide nanosheets, enabling synergistic immunotherapy for hepatocellular carcinoma and NIR‐III‐triggered photodynamic therapy via a H_2_O_2_/pH dual‐gating mechanism (Figure 9f).^[117]^ The core innovation exploits acidic conditions to concurrently trigger rupture of hybrid cancer CM/thylakoid membrane (TK) coatings and membrane anchoring by pH‐sensitive peptides (pHLIP). This precise spatial control facilitates membrane‐localized PDT under 1550 nm laser irradiation, inducing GSDMD‐dependent pyroptosis. CTEP reverses the immunosuppressive TME through pyroptosis‐mediated release of DAMPs and pro‐inflammatory cytokines, significantly potentiating αPD‐L1 immunotherapy efficacy while suppressing both primary and distal tumor growth. Furthermore, the intrinsic catalase‐mimetic activity of TK membranes alleviates tumor hypoxia, substantially enhancing PDT efficiency to achieve potent tumor eradication.

#### 3.2.3 Photothermal therapy

Photothermal therapy (PTT), an optical tumor treatment strategy based on the photothermal conversion effect, achieves precise ablation of tumor tissue through the selective transformation of light energy into heat. Owing to its minimally invasive nature, durability, and high safety profile, this technique has garnered significant attention in the field of malignant tumor treatment. However, conventional PTT protocols typically require high‐intensity laser irradiation to elevate local temperatures above 50°C. While this effectively ablates tumor tissue, it concurrently poses a risk of irreversible thermal damage to surrounding healthy structures. Although reducing the treatment temperature (<42°C) mitigates these adverse effects, it often results in substantially compromised tumor cell thermotolerance and diminished therapeutic efficacy. Furthermore, ROS generation‐essential for PTT‐exhibits intrinsic oxygen dependence. The characteristically hypoxic TME thus severely compromises PTT efficacy. Consequently, the development of mild yet potent PTT modalities that ensure robust therapeutic efficacy while maximizing preservation of normal tissues has emerged as a pivotal scientific challenge in advancing the clinical translation of optical cancer treatments. Building on this concept, Xu's team engineered surface defect‐enriched Cu‐LDH (d‐Cu‐LDH) via acid etching and intercalated the photosensitizer indocyanine green (ICG) into its layered structure.^[118]^ This d‐Cu‐LDH/ICG nanocomposite achieved high photothermal conversion efficiency under low‐intensity 808 nm laser irradiation (0.23 W/cm^2^), concurrently facilitating enhanced ^1^O_2_ generation. In murine 4T1 tumor models, a single 5‐min irradiation with 808 nm light (0.23 W/cm^2^) induced substantial suppression of tumor growth, unequivocally demonstrating the potent therapeutic efficacy of the d‐Cu‐LDH/ICG nanoplatform.

**FIGURE 10:**
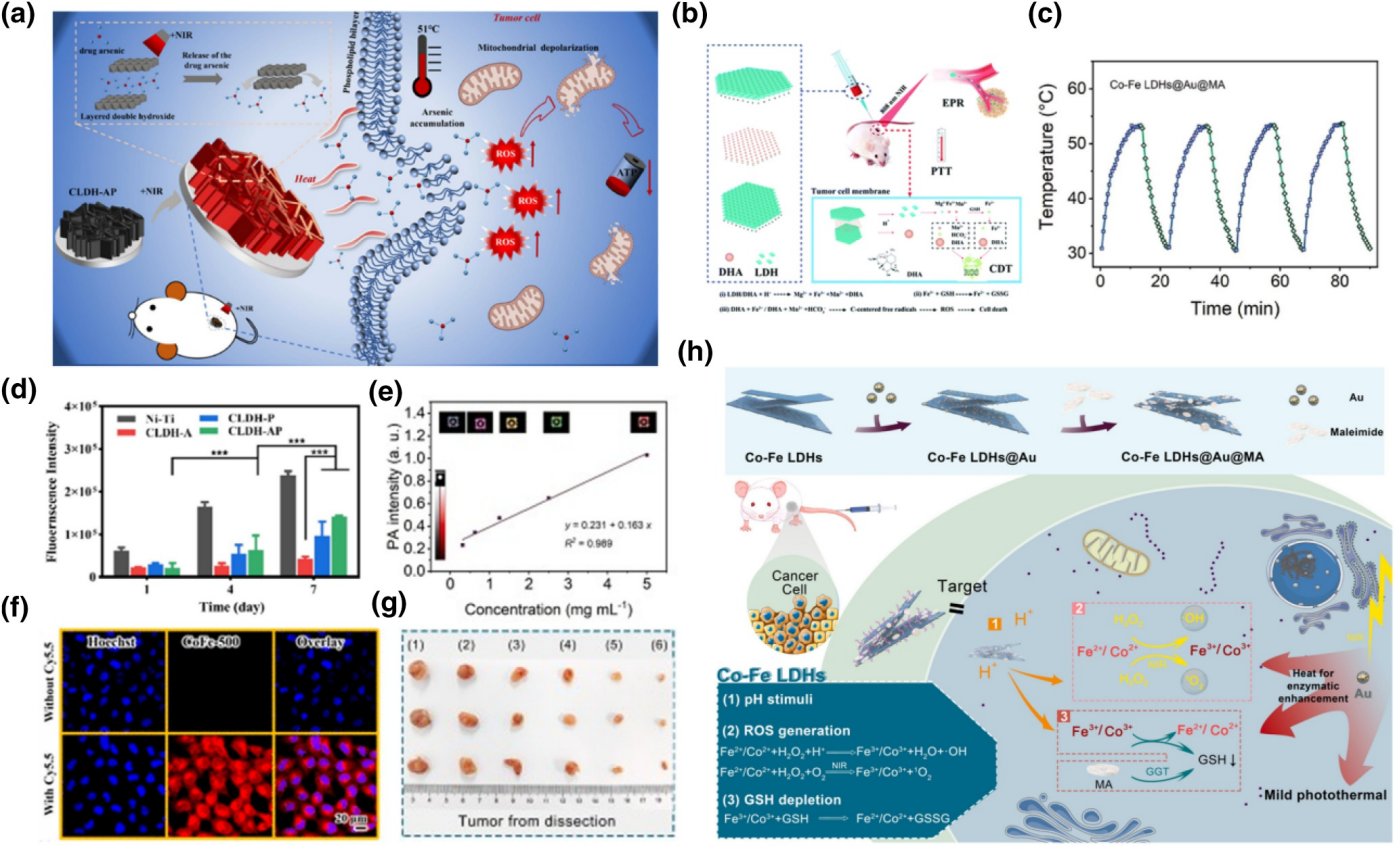
LDH‐based nanomaterials for photothermal therapy. (a) Schematic diagram illustrating the mechanism of CLDH‐AP for cancer therapy under NIR irradiation. Reproduced with permission.^[120]^ Copyright 2024, Elsevier. (b) Schematic diagram illustrating the synthesis process and therapeutic mechanism of DHA/MnMgFe‐LDH. Reproduced with permission.^[122]^ Copyright 2020, Royal Society of Chemistry. (c) Photothermal stability of CoFe‐LDH@Au@MA. Reproduced with permission.^[121]^ Copyright 2023, Elsevier. (d) Proliferation result of HIBEpic cells cultured on the Ni‐Ti, CLDH‐A, CLDH‐P, and CLDH‐AP samples at different time points (*n* = 3). Reproduced with permission.^[120]^ Copyright 2023, Elsevier. (e) Corresponding PA intensity of CoFe‐LDHs@Au@MA solutions at different concentrations. Reproduced with permission.^[121]^ Copyright 2023, Elsevier. (f) Confocal microscopy images of HeLa cells cultured with CoFe‐500 loading Cy5.5. Reproduced with permission.^[123]^ Copyright 2020, American Chemical Society. (g) Tumor growth in mice from each group after treatment and (h) Schematic diagram illustrating the synthesis process and therapeutic mechanism of CoFe‐LDHs@Au@MA. Reproduced with permission.^[121]^ Copyright 2023, Elsevier.

Milad Ashrafizadeh et al. employed ortho‐, meta‐, and para‐poly‐phenylenediamine‐exhibiting superior optical properties and aqueous solubility compared to conventional conductive polymers‐as photothermally active components loaded onto CuAl‐LDH nanosheets.^[119]^ This design enables precise spatiotemporal control over PTT under 808 nm laser irradiation. Furthermore, by co‐loading the chemotherapeutic agent DOX and CRISPR plasmid payloads onto the LDH carrier, the platform harnesses synergistic interplay between photothermal effects, drug release, and gene delivery. This multimodal integration significantly enhances antitumor efficacy while simultaneously optimizing the precision and therapeutic safety profile of PTT. Addressing the therapeutic challenge of portal vein tumor thrombosis, Liu et al. successfully developed a multifunctional localized treatment platform by engineering an in situ‐grown PPy‐coated, arsenic‐loaded layered double hydroxide composite film on a nickel‐titanium alloy substrate.^[120]^ This integrated system simultaneously delivers photothermal therapy, chemotherapy, and antibacterial functionality (Figure 10a). This platform demonstrates exceptional near‐infrared photothermal conversion performance: under low‐power‐density (0.5 W/cm^2^) NIR irradiation, the material surface temperature rapidly reaches 51°C within merely 8 minutes. It exhibits outstanding photothermal stability maintaining consistent performance through five on/off cycles with a remarkable photothermal conversion efficiency of 27.2%. This highly efficient photothermal effect not only directly ablates tumor cells but also accelerates thermo‐responsive arsenic ion release, collectively enabling dual enhancement of therapeutic efficacy (Figure 10d). Notably, the polypyrrole coating functions as an intelligent “barrier” in the absence of NIR light stimulation, effectively suppressing the spontaneous release of arsenic ions. This significantly reduces the toxicity of the material to normal tissues and enables on‐demand, controlled drug release. In terms of the mechanism of action, the platform exerts a remarkable synergistic effect between photothermally induced oxidative stress responses, mitochondrial damage. And impairment of ATP synthesis, and the chemotherapeutic action of arsenic ions, thereby achieving efficient killing of tumor cells. Meanwhile, the material exhibits excellent antibacterial activity against *Staphylococcus aureus* and *Escherichia coli*, which can effectively mitigate the risk of bacterial colonization following implantation.

Beyond conjugated conductive polymers like polypyrrole where extended π‐conjugated systems enable strong NIR photon absorption, subsequently converting light energy into heat via non‐radiative relaxation pathways involving molecular vibrations and rotations‐metallic nanoparticles or elements (e.g., Au, Ag, Cu, Pt) generate photothermal effects through distinct mechanisms. Under wavelength‐specific (particularly NIR) irradiation, these materials exhibit localized surface plasmon resonance concentrating electromagnetic energy on their surfaces. This energy dissipates as heat through electron‐phonon relaxation processes, elevating temperatures both within the particle and its local microenvironment. In the CoFe‐LDHs@Au@MA nanozyme engineered by Yang et al., gold nanoparticles (Au NPs) harness mild photothermal properties under 808 nm laser irradiation.^[121]^ This not only directly induces tumor apoptosis through hyperthermia but also potentiates the catalytic activity of LDHs, synergizing with enzyme‐catalyzed ROS generation to amplify antitumor efficacy (Figure 10c,e,g,h). Similarly, Zhao doped Mn into dihydroartemisinin (DHA)‐loaded layered double hydroxides (denoted as MnMgFe‐LDH/DHA).^[122]^ This nanocomposite significantly optimized its photothermal properties through Mn doping, endowing MnMgFe‐LDH with broader and stronger absorption capacity in the NIR region, excellent photothermal conversion efficiency, and stable photothermal performance even after five irradiation cycles, which effectively addresses the issue of insufficient performance in traditional photothermal materials. Concurrently, BSA surface modification confers excellent biocompatibility and enables selective tumor accumulation via the EPR effect. This facilitates spatially confined photothermal heating at the target site, significantly inhibiting tumor growth while avoiding discernible damage to normal tissues (Figure 10b). As shown in Figure 10f Zhou's advancement in photothermal therapy centers on augmenting anticancer efficacy through controlled defect engineering in CoFe‐LDH.^[123]^ Subjecting synthesized CoFe‐LDH nanosheets to heat treatment at 200–800°C under argon atmosphere protection induces atomic rearrangement within the LDH lattice. This thermal modulation disrupts the coordination environments of select Co atoms, generating Co^2+^ defects‐yielding a spectrum of defect‐engineered CoFe‐based photothermal agents with tailored properties. This defect engineering strategy‐precisely modulating annealing temperature to alter crystallinity, particle size, and metal defect concentration‐not only amplifies photothermal performance but also enhances photostability, ensuring sustained and stable heat generation under repeated NIR irradiation, which is critical for continuous tumor ablation. In vitro and in vivo studies further confirm that defect‐rich CoFe‐LDH effectively accumulates at tumor sites, rapidly elevating local temperatures to apoptosis‐inducing levels upon NIR exposure while exhibiting excellent biocompatibility with negligible damage to normal tissues. Collectively, this work establishes a paradigm‐shifting strategy for optimizing LDH‐based photothermal agents and pioneers a framework for highly efficient yet safe photothermal cancer therapy.

#### 3.2.4 Sonodynamic therapy

Ultrasound (US), as an exogenous physical stimulus, is extensively utilized in clinical diagnostic imaging and therapeutic interventions owing to its deep tissue penetration capability, high spatial resolution, and non‐invasive nature.^[124]^ Ultrasound dynamic therapy harnesses sonosensitizers to generate ROS upon US activation. This process fundamentally relies on the cavitation effect‐where continuous expansion and implosive collapse of microbubbles release concentrated energy. Such energy discharge induces sonoluminescence or localized thermal decomposition, thereby activating sonosensitizers for ROS generation. The development of efficient sonosensitizers is pivotal for advancing ultrasound dynamic therapy SDT, with current agents primarily categorized as organic or inorganic. Traditional organic sonosensitizers predominantly comprise porphyrin derivatives and other organic photosensitizing molecules. However, inherent limitations‐including pronounced hydrophobicity, low bioavailability, and rapid systemic clearance‐severely compromise their therapeutic utility and hinder the clinical translation of SDT.

**FIGURE 11:**
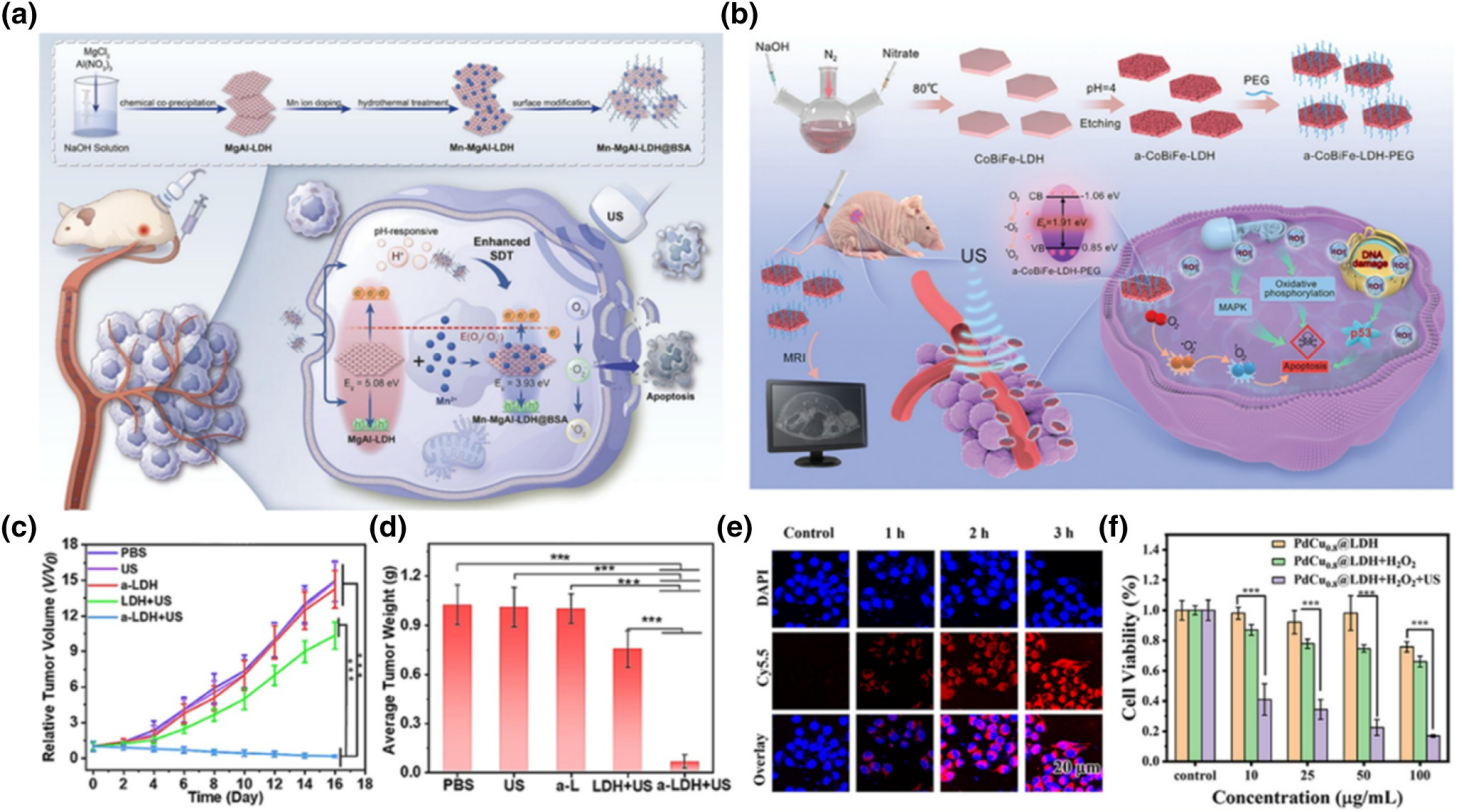
LDH‐based nanomaterials for Sonodynamic Therapy. (a) Schematic diagram illustrating the synthesis process of Mn‐MgAl‐LDH@BSA and its mechanism for achieving SDT efficacy. Reproduced with permission.^[126]^ Copyright 2025, Elsevier. (b) Schematic diagram illustrating the synthesis of a‐CoBiFe‐LDH‐PEG and its implementation for MRI/SDT. Reproduced with permission.^[127]^ Copyright 2024, Wiley‐VCH. (c) Tumor growth curves of 4T1 tumor‐bearing mice after various treatments (PBS, US (40 kHz, 3 W cm^−2^, 6 min), a‐CoBiMn‐LDH‐PEG, CoBiMn‐LDH‐PEG + US, a‐CoBiMn‐LDH‐PEG + US). Error bar represents ± S.D. (*n* = 6). (d) Average tumor weight of each group of mice after 16 days of treatment. Error bar represents ± S.D. (*n* = 6). Reproduced with permission.^[128]^ Copyright 2024, Springer Nature. (e) CLSM images of the cellular uptake of PdCux@LDH in HeLa cells. (f) Cytotoxicity study of PdCux@LDH incubated with HeLa cells under various treatments (*n* = 3 per group). Reproduced with permission.^[129]^ Copyright 2024, American Chemical Society.

Researchers have optimized the performance of LDH as a sonosensitizer through element doping and band structure modulation. Cheng achieved the synthesis of MgMnFe‐LDH via a local elemental rearrangement strategy in which Mn^2+^ partially substitutes Mg^2+^.^[125]^ This modification shortens the Fe‐O bond length, optimizes the band gap, and results in enhanced sonocatalytic performance under ultrasound irradiation. Concomitantly, this material depletes GSH to disrupt tumoral redox homeostasis, triggering intracellular ROS bursts that potentiate cancer cell apoptosis. Furthermore, substitutional Mn^2+^ ions efficiently promote DC maturation and reprogram the immunosuppressive microenvironment. This multimodal action synergistically amplifies therapeutic outcomes through concerted oxidative and immunomodulatory mechanisms. In a cognate approach, Li et al. engineered Mn^2+^‐doped magnesium‐aluminum layered double hydroxide functionalized with BSA (Mn‐MgAl‐LDH@BSA) (Figure 11a).^[126]^ Manganese doping enables precise modulation of MgAl‐LDH's band structure, narrowing the bandgap from approximately 5.08–3.93 eV. This electronic restructuring significantly enhances ultrasound energy absorption efficiency and promotes electron‐hole pair generation under acoustic excitation. Concurrently, manganese ions serve as electron‐trapping sites that efficiently capture activated electrons and retard the electron‐hole recombination kinetics. This promotes the reaction between trapped electrons and molecular oxygen to generate abundant ROS.

In the practical application of ultrasound‐driven SDT, current sonosensitizers often exhibit insufficient responsiveness to ultrasound and poor performance in ROS generation. To develop more efficient sonosensitizers, acid etching has been employed to treat LDHs, introducing structural defects that enhance its catalytic activity.as shown in Figure 11b, Liang et al. engineered a well‐defined polycrystalline‐to‐amorphous phase transition in CoBiFe‐LDH nanosheets through controlled acid etching.^[127]^ The amorphous CoBiFe‐LDH (a‐CoBiFe‐LDH), benefiting from phase transition‐induced defects and modified electronic structures after acid etching, exhibits superior ROS‐generating activity under ultrasonic irradiation‐approximately 6.9‐fold higher than commercial TiO_2_ sonosensitizers. Chen et al. engineered a dual‐functional sonosensitizer for MRI‐guided sonodynamic therapy by subjecting hydrothermally synthesized crystalline CoBiMn‐LDH to controlled acid etching.^[128]^ Defect‐induced bandgap narrowing and enhanced electron‐hole separation efficiency significantly amplify ROS generation under ultrasonic irradiation, yielding approximately 3.3‐fold and 8.2‐fold higher ROS production compared to pristine crystalline CoBiMn‐LDH and commercial TiO_2_, respectively. Furthermore, under ultrasound irradiation, LDH activates the p53 signaling pathway and the apoptotic pathway, demonstrating a favorable tumor suppression effect both in vitro and in vivo (Figure 11c‐d).

In addition, Zhou et al. designed PdCu_x_@LDH alloy nanoenzymes, which solved the key problem of limited catalytic efficiency of traditional sonosensitizers in the tumor microenvironment.^[129]^ By strategically tailoring the Pd/Cu alloy ratio, this system optimizes the electronic configuration and active site distribution, thereby amplifying OXD‐like and CAT‐like enzymatic activities (Figure 11e‐f). This synergy enables sustained high‐yield ^1^O_2_ generation while simultaneously decomposing overexpressed GSH in the tumor microenvironment to minimize non‐therapeutic ROS depletion. Furthermore, NADH consumption perturbs oncogenic metabolic reprogramming, inducing mitochondrial dysfunction and triggering apoptosis through coordinated bioenergetic disruption. Ultrasound irradiation not only potentiates the multi‐enzyme activities of the nanozyme but also significantly enhances the generation efficiency of ROS, including superoxide radicals. This strategy overcomes the inherent limitation of inadequate tissue penetration depth in conventional photothermal therapy.

Yang's team designed a piezoelectric NiFe‐LDH bilayer nanostructure, which exhibits piezoelectric properties under mechanical stimuli such as ultrasound, thereby efficiently catalyzing H_2_O_2_ to generate substantial ROS and directly inducing oxidative damage in tumor cells.^[130]^ This piezocatalytic mechanism enables LDH to serve as a safe and effective sonosensitizer for tumor therapy, while overcoming the reliance on TME conditions for ROS production. Even at low H_2_O_2_ concentrations, it maintains high efficiency in generating ROS, addressing the bottleneck of insufficient endogenous H_2_O_2_ in tumors. By continuously optimizing the ROS generation efficiency of the LDH sound‐sensitive group under US excitation through various strategies, a progressive improvement from basic sound catalysis to multi‐mode collaborative anti‐tumor was achieved. These efforts not only solved the problems of low efficiency of traditional sound‐sensitive groups and the limitations of the tumor TME, but also provided a design framework and theoretical basis for safely and effectively eliminating deep tumors.

#### 3.2.5 Immunotherapy

Immunotherapy represents a revolutionary advancement in cancer therapeutics, fundamentally leveraging the activation or augmentation of the patient's own immune system to combat malignancies. Immune checkpoint inhibitors‐notably targeting PD‐1/PD‐L1 and CTLA‐4 pathways‐have demonstrated remarkable efficacy across diverse cancers, including melanoma and non‐small cell lung cancer. Furthermore, chimeric antigen receptor T‐cell therapy‐engineered to genetically reprogram T cells for tumor‐specific recognition and eradication—has demonstrated profound curative potential in hematologic malignancies. Nevertheless, immunotherapy continues to confront significant challenges, including suboptimal efficacy against solid tumors and immune‐related adverse events.

**FIGURE 12:**
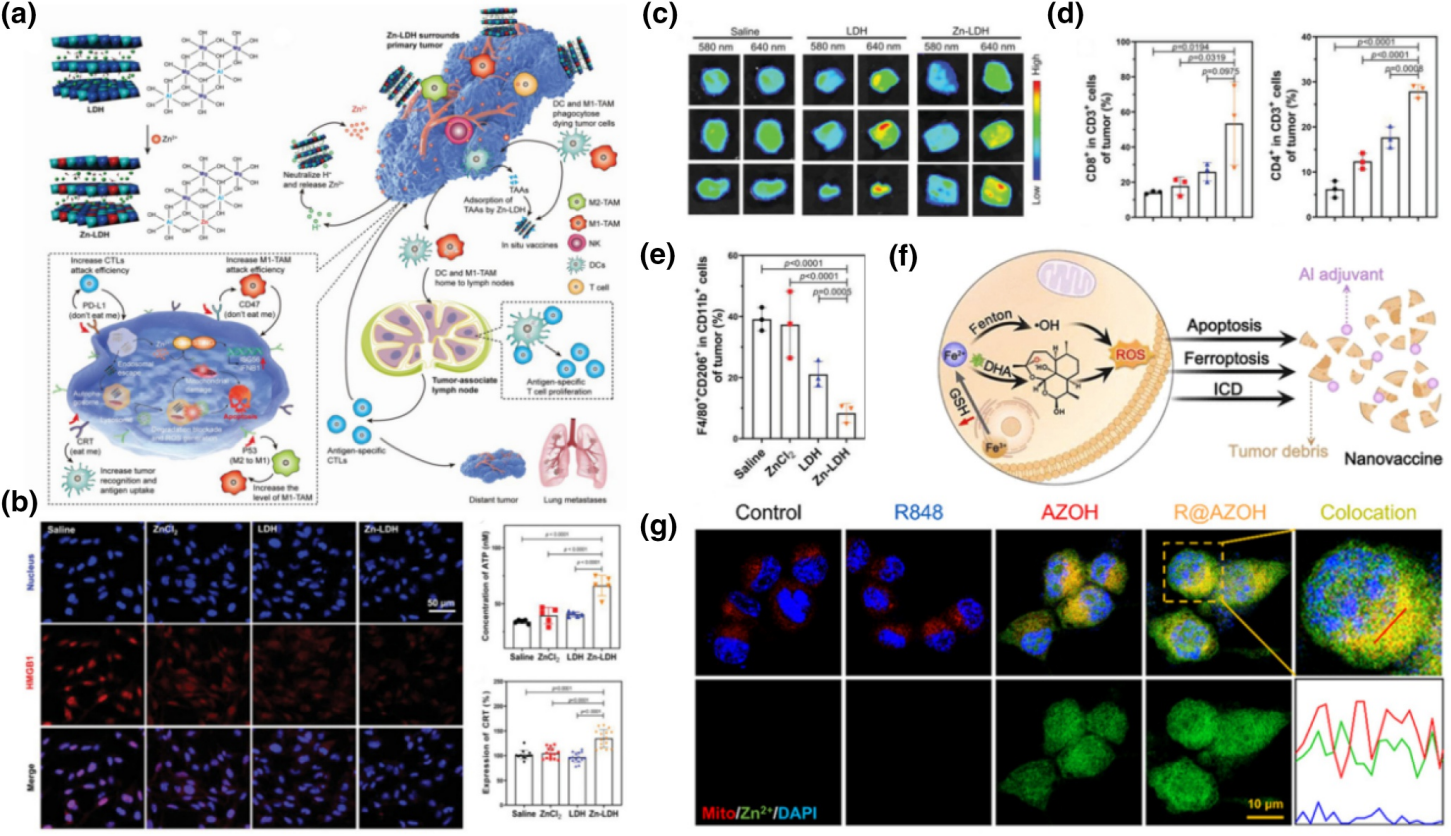
LDH‐based nanomaterials for immunotherapy. (a) Schematic diagram illustrating the synthesis process of Zn‐LDH and its mechanism for eliciting immune effects, and (b) CLSM images of intracellular HMGB1 distribution in B16F10 tumor cells treated with ZnCl2, Layered double hydroxide (LDH), and Zn‐LDH. And quantitative analysis of the ATP release in B16F10 tumor cells treated with ZnCl_2_, LDH, and Zn‐LDH. q‐PCR analysis of the relative levels of CRT (c) SNARF‐1 fluorescence (measured at 580 and 640 nm) images of tumors collected from mice treated with saline, LDH and Zn‐LDH for 24 h. (d) Levels of CD8+ T cells, CD4+ T cells cells in B16F10 tumors and (e) Levels of M2‐like TAMs cells in B16F10 tumors. (f) Schematic illustration of the mechanism of Zn‐LDH‐mediated TME modulation. Reproduced with permission.^[125]^ Copyright 2022, Wiley‐VCH. (g) Fluorescence images and quantitative curves of mouse tumors over time post‐administration, fluorescence distribution in tumors and organs, and fluorescence staining of CD31, HIF‐1α, CD4, and CD8 after FZOH treatment. Reproduced with permission.^[127]^ Copyright 2025, American Chemical Society.

To break through these bottlenecks, scientists have turned their attention to the metabolic ecology of the tumor microenvironment; neutralizing the acidic TME caused by abnormal tumor metabolism has become a key strategy for restoring the functions of effector cells and reversing immune suppression. Zhang et al. addressed this challenge by synthesizing layered double hydroxide nanoparticles that effectively neutralize intratumoral acidosis while concurrently inhibiting tumor‐protective autophagy.^[131]^ Following peritumoral injection, LDH NPs effectively neutralize excess H^+^ ions within the TME, thereby reversing the immunosuppressive microenvironment. This intervention significantly reduces M2 macrophage polarization while enhancing M1 phenotype prevalence. Concurrently, the proportions of tumor‐infiltrating CD8+ and CD4+ T cells are substantially elevated, collectively suppressing tumor growth in treated murine models. Notably, essential metal ions‐including Ca^2+^, Mn^2+^, and Zn^2+^‐demonstrate inherent immunomodulatory properties, positioning them as promising next‐generation immunomodulatory agents for cancer immunotherapy. Ling's team successfully synthesized Zn‐LDH using an ion exchange method; Zn^2+^ released by the dissolution of LDH in the TME activates the cGAS‐STING signaling pathway through binding to DNA released from damaged mitochondria, thereby inducing ICD and triggering robust antitumor immunity.^[132]^ Furthermore, Zn‐LDH also reduces the levels of tumor immune checkpoints (PD‐L1 and CD47), thereby enhancing the immune resistance of the tumor. Following administration in mice, Zn‐LDH effectively activates intratumoral antitumor immune cells such as M1‐type tumor‐associated macrophages (M1‐TAMs), CTLs, and NK cells, subsequently impairs endolysosomal function to block the autophagic pathway and induces mitochondrial damage in tumor cells. The immune effect elicited by Zn‐LDH successfully inhibits the growth or metastasis of melanoma and breast cancer in mice (Figure 12a–f).

However, such systems still mainly rely on the activation of a single signaling pathway, resulting in limited immune amplification effects and insufficient adaptability to the complex tumor microenvironment. Based on these limitations, research has gradually shifted towards the development of multi‐mechanism coordinated regulation. Luo's team engineered Pt‐CoNi‐LDHs via a templating method, subsequently encapsulating them within erythrocyte membranes to form biomimetic RmPLH nanoplatforms.^[133]^ Under ultrasound stimulation, RmPLH concurrently liberates substantial ROS and Pt^2+^ ions. The ROS cascade induces ferroptosis and pyroptosis in tumor cells through oxidative stress and mitochondrial membrane potential dissipation, while Pt^2+^ provokes DNA damage via Pt‐DNA adduct formation. The large accumulation of ROS triggers pyroptosis in cells, releases pro‐inflammatory factors, and induces the release of DAMPs such as CRT, HMGB1, and ATP. Subsequently, the cGAS‐STING pathway is activated, effectively triggering ICD. Strategic integration of RmPLH with PD‐1 blockade profoundly amplifies ICD efficacy, eliciting robust antigen‐specific T‐cell immunity. The establishment of this system demonstrates its excellent potential for combined immunotherapy.

Promoting effective antitumor immune responses is of great importance in immunotherapy; however, immunotherapy remains challenged in combating tumors due to the immunosuppressive microenvironment characterized by antigen scarcity, impenetrability, and enhanced immunosuppressive metabolism. In contrast, LDH possesses a positively charged surface and a high specific surface area, which enables easy loading of large amounts of immunomodulators and antigens through simple mixing, thereby triggering effective cancer immunotherapy. Building on this, Cheng's team synthesized aluminum‐zinc hydrotalcite (AZOH) as a pyroptosis inducer, which was further loaded with the Toll‐like receptor agonist R848 to form the R@AZOH nanoplatform.^[134]^ This nanoplatform effectively triggers pyroptosis in CT26 cells via Zn^2+^ overload‐induced mitochondrial dysfunction and its downstream caspase‐1/GSDMD pathway, resulting in the release of inflammatory cytokines, membrane rupture, and ICD. Furthermore, the excellent specific surface area and porosity of R@AZOH enable it to act as antigen traps to facilitate antigen presentation, thereby achieving dual stimulation of DC maturation through both this antigen‐presenting effect and the activation of Toll‐like receptor agonist R848. Experimental evidence demonstrates that R@AZOH can rapidly initiate innate immunity and prolong adaptive immune responses, resulting in the suppression of tumor growth, activation of immune cells, and induction of a “hot” tumor niche. In combination with the immune checkpoint inhibitor (αCTLA‐4), potent antitumor immunity is further enhanced, with the inhibitor suppressing both primary and distant tumors as well as promoting systemic immune activation (Figure 12g). Although this strategy has achieved a breakthrough at the level of immune activation, there is still a need to enhance the activation of specific immune checkpoint pathways. Yu's team developed a pH‐responsive ultrathin layered double hydroxide multifunctional nanoplatform (CS@P), which can simultaneously deliver small interfering RNA targeting the nuclear immune checkpoint NR2F6 and achieve asynchronous blockade of the cell surface immune checkpoint PD‐L1, thereby triggering a synergistic antitumor immune response. Moreover, the photothermal effect generated by the nanoplatform under laser irradiation can remodel the immunosuppressive tumor microenvironment, converting “cold” tumors into “hot” tumors, and further significantly enhance the combined immunotherapy efficacy based on NR2F6 gene silencing and PD‐L1 blockade.^[135]^

As a key strategy in cancer immunotherapy, cancer vaccines can specifically activate the human immune system, enabling it to recognize and eliminate tumor cells. This leads to efficient and systematic immune activation. For instance, Moderna's mRNA‐4157, in combination with PD‐1 inhibitors in Phase III clinical trials for melanoma, has demonstrated excellent progression‐free survival benefits. Regarding vaccine delivery technologies, the use of nanoparticles for delivery has become a mainstream strategy, among which metal nanoparticles have attracted significant attention due to their ability to serve as both immune adjuvants and delivery carriers. Wang's team innovatively designed and developed manganese‐doped layered double hydroxides (MLDHs), achieving efficient lymph node‐targeted delivery through precise regulation of the particle size of LDHs.^[136]^ MLDHs are internalized by lymph node‐resident DCs, activating the STING signaling pathway to promote DC maturation and antigen‐presenting function. To further enhance antitumorimmune responses, the team loaded the STING agonist vidutolimod (DMXAA) into MLDHs, constructing the MLDHs@DMXAA nanocomposite system. Achieve the synergistic enhancement of both endogenous and exogenous immune stimulation.

Under the influence of lasers and other methods, LDHs can cause local overheating and ROS, directly killing the primary tumor. The ICD triggered by this process can release tumor‐associated antigens (TAAs) and abundant DAMPs. The unique two‐dimensional structure and positive charge of LDHs help DCs to take up TAAs. Moreover, LDHs can reshape the immunosuppressive tumor microenvironment, accelerate the maturation of DCs, and trigger a strong systemic CD8+ T cell response. When used in combination with immune checkpoint inhibitors, it can induce a potent targeted effect and long‐term immune memory. Liang's team obtained LDH@I‐IPA‐PEG by intercalating iodine‐functionalized isophthalic acid (I‐IPA) into ZnAl‐LDH and then modifying it with PEG. This nanoscale platform effectively addresses the core bottlenecks of short triplet state lifetime of the photosensitizer and low ^1^O_2_ production rate.^[82]^ The LDH confinement effect not only narrows the band gap to achieve efficient NIR‐II excitation, but also significantly extends the triplet lifetime by three orders of magnitude through the mechanism of inhibiting reverse intersystem crossing (RISC). Furthermore, LDH@I‐IPA‐PEG demonstrated remarkable abilities in inducing tumor cell apoptosis and ICD in vivo, achieving efficient inhibition of both primary and metastatic tumors.

In addition to the traditional PTT/PDT‐induced immune responses, recent studies have highlighted the significant potential of the LDH platform in promoting novel metabolic cell death. For instance, Guan's team developed a RuRh@LDH nanoenzyme. Unlike traditional enzyme therapies, this LDH‐based nanoreactor can initiate self‐catalytic cascades, efficiently generating O_2_, providing fuel for glucose oxidase‐like reactions, and causing rapid depletion of glucose in the tumor microenvironment.^[137]^ The depletion of glucose not only disrupts energy metabolism but also triggers Disulfidptosis. Notably, this metabolic collapse mediated by LDH can effectively reshape the immunosuppressive microenvironment and significantly promote the infiltration of cytotoxic T cells, thereby triggering a potent systemic anti‐tumor immune response. This paradigm shift indicates that LDH is not only a structural carrier but also a highly active metabolic and immune regulatory factor. In addition to triggering the immune response through physical means, LDHs are also excellent carriers for collaborative gene immunotherapy. Wang's team designed an LDH nanoplatform (TGR‐BLDHs) that was co‐modified with cRGD and BSA.^[138]^ By leveraging the excellent interlayer electrostatic adsorption and nucleic acid protection capabilities of LDH, the novel tumor suppressor gene plasmid (pTMEM163) and the STING pathway agonist (cGAMP) were jointly delivered into lung cancer cells. After entering the cells through specific targeting and escaping from lysosomes, this platform, on one hand, uses the released plasmid to restore the expression of TMEM163, directly inhibiting the malignant proliferation of tumors at the genetic level. On the other hand, it uses the released cGAMP to strongly activate the cGAS‐STING signaling pathway within tumor cells and antigen‐presenting cells, inducing the release of a large amount of type I interferons and triggering a strong systemic anti‐tumor immune response. Thus, it successfully achieved a deep synergistic intervention of targeted gene repair and anti‐tumor immune activation.

Overall, the research on LDH‐based nanomaterial platforms in tumor immunotherapy has undergone a gradual development process from single immune activation, to multi‐mechanism synergy, to microenvironment remodeling and precise regulation. Table 2 presents a summary and comparison of different treatment methods for LDH. Although significant progress has been made, there are still challenges in adapting to the complex tumor microenvironment, achieving structural controllability, and achieving clinical translation. In the future, further optimization is needed in terms of mechanism integration and translatability.

**TABLE 2:** Summary and comparison of the LDH‐Based treatment platform.

Nanoplatform	Treatment method	Advantage	Boundedness	Reference
ICG/Fe‐LDH@PEG	CDT	Near‐infrared light response	Long‐term probiotic activity is hard to sustain	[104]
Probiotics/CaO_2_/CoFeCe‐LDH	CDT	Excellent tumor targetability	Long‐term probiotic activity is hard to sustain	[105]
ATX/NiFe‐LDH	CDT	The Brønsted base sites promote the generation of free radicals.	Catalytic efficiency needs improvement	[107]
HE‐LDH	CDT	Multimetallic synergy	The control of metal ion release needs to be optimized.	[108]
Ce6/LDH	PDT	Improve the water solubility and targeting ability of Ce6	The penetration depth is limited.	[111]
LA + CoCuMo‐LDH	PDT	Excellent targeting property	Insufficient catalytic efficiency	[113]
CoMo‐LDH/NiMo‐LDH	PDT	Defect engineering; Deep tissue penetration	The NIR‐III light source is not yet widely available	[114]
d‐Cu‐LDH/ICG	PTT/PDT/CDT	Multimodal synergistic therapy	Relying on external light stimulation	[118]
CoFe‐LDHs@Au@MA	PTT/CDT	Multi‐metal site synergistic catalysis	Inadequate toxicity assessment	[121]
MgMnFe‐LDH	SDT/Immunotherapy	Tune the bandgap	Depend on the US	[125]
PdCu_x_@LDH	SDT	Efficient enzyme‐mimicking activity	The specific active site is not clear.	[129]
NiFe‐LDH	Piezocatalytic/SDT	Piezoelectric catalysis, independent of H_2_O_2_ concentration	The stability of the piezoelectric effect in the body needs to be verified.	[130]
Zn‐LDH	cGAS‐STING immune activation	No immune agonist required	Single‐pathway activation leads to limited immune amplification	[132]
MgCaFe‐LDH	SDT/CDT/Immunotherapy	Ca2+ regulates the energy band	High metabolic toxicity	[139]
NiCoTi‐LDH	PDT/CDT	Without the need for additional photosensitizers	Limited light penetration depth	[140]

#### 3.2.6 Synergistic therapy

Although CDT, PTT and SDT all have unique advantages in tumor treatment, their individual applications are often limited to varying degrees. For instance, the efficiency of CDT is highly dependent on the concentration of H_2_O_2_ and acidic conditions in the tumor microenvironment, while the heterogeneity of solid tumors often leads to insufficient local reactive substrates, and the limited activity of endogenous Fenton catalysts restricts the sustained and efficient generation of ROS. Although PTT can achieve local rapid hyperthermia to kill tumor cells, its tissue penetration depth is limited by the physical properties of near‐infrared light, making it difficult to effectively act on deep‐seated tumors, while excessively high thermal doses may cause damage to surrounding normal tissues. Although SDT possesses deeper tissue penetration capability, its therapeutic efficacy is limited by the ROS production efficiency of sonosensitizers and the hypoxic microenvironment at the tumor site, and single ultrasound stimulation makes it difficult to achieve precise drug‐controlled release and synergistic enhancement.^[141]^ Furthermore, the single treatment mode often fails to achieve precise drug release and multi‐pathway coordinated killing. Given these limitations, multi‐modal coordinated treatment has gradually become an effective strategy for efficiently killing tumors. The photothermal effect not only increases local temperature at the tumor site, promotes blood perfusion and oxygen supply to enhance the kinetic efficiency of the Fenton reaction, but also increases CM permeability through thermal effects to accelerate the internalization of metal ions and sonosensitizers. The cavitation effect and mechanical force of ultrasound can further facilitate deep penetration of nanodrugs, while synergistically, ROS generated by sonosensitizers and free radicals produced via chemodynamics form a cumulative effect of oxidative stress, achieving synergistic killing through multimodal ROS.^[142]^ The combined application of these three modalities can reduce the required dosage of single therapies, minimize systemic toxicity, and achieve more precise and efficient tumor therapy through spatiotemporal complementary effects more critically, numerous studies have demonstrated that such synergistic therapy induces significant ICD, releasing large amounts of tumor antigens and DAMPs, which effectively initiate antitumor immune responses and preliminarily convert “cold tumors” to “hot tumors”. Nevertheless, the intensity of innate immune activation and adaptive immune priming triggered by local therapy is often insufficient to fully overcome the profound immunosuppression of the tumor microenvironment, nor can it easily elicit a robust and durable systemic antitumor immune response to eliminate distant metastases or prevent recurrence. To address this bottleneck, combination with immunotherapy (particularly immune checkpoint blockade) has achieved more efficient tumor killing.

**FIGURE 13:**
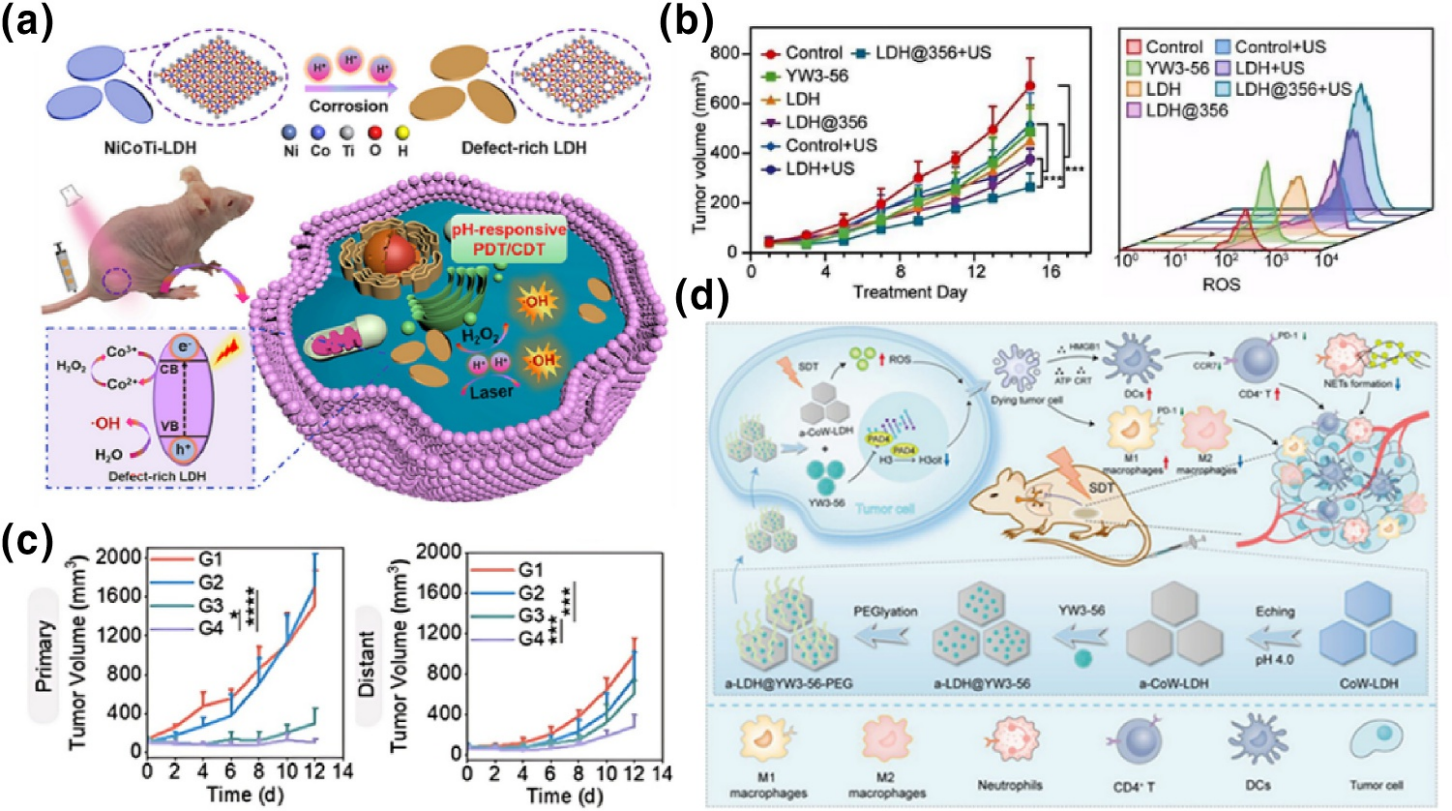
LDH‐based nanomaterials for synergistic therapy. (a) Schematic illustration of pH‐responsive PDT/CDT synergistic treatment in the presence of NiCoTi‐LDH. Reproduced with permission.^[140]^ Copyright 2024, KeAi Communications Co. Ltd. (b) Tumor volume curves of different groups of mice recorded every 2 days and Representative results of ROS detected by flow cytometry. (c) Growth curves of primary and distant tumors in different treatment groups. (G1: Control, G2: αPD‐L1, G3: ZCA‐LDH@DSF, G4: ZCA‐LDH@DSF+αPD‐L1) (d) Schematic illustration of the preparation of a‐LDH@356‐PEG nanosheets and their application in synergistic SDT/immunotherapy. Reproduced with permission.^[144]^ Copyright 2024, Wiley‐VCH.

Tan carried out the sulfidation of ultrathin CuFe‐LDH nanosheets through simple hydrothermal treatment, thereby preparing ultrathin two‐dimensional CuFe_2_S_3_ nanosheets.^[142]^ After PEG modification, this material exhibited broadband absorption characteristics in the second near‐infrared window (NIR‐II), with a photothermal conversion efficiency of up to 55.86% at 1064 nm. The local high temperature generated by the photothermal effect can synergistically promote the Fenton reaction activity at the lesion site, thereby significantly enhancing the efficiency of hydroxyl radical production by CDT. However, this system still mainly relies on light stimulation, and its applicability to deep tumors is still limited to a certain extent. To further enhance the penetration ability of the system, Liu et al. developed MgCaFe‐LDH (MCF), which effectively improves the sonodynamic performance by introducing Ca^2+^ to regulate the electronic structure.^[139]^ At the same time, Fe^3+^ participates in the Fenton reaction to achieve CDT, and forms a synergy with SDT. Moreover, this system can also regulate the activity of immune cells through the regulation of Mg^2+^ and Ca^2+^, induce the polarization of M1‐type macrophages and activate the T‐cell immune response. Compared with a single PTT system, this strategy demonstrates greater advantages in deep tumor treatment and immune activation, but there is still room for further optimization in its response to the tumor microenvironment. Based on this, the research further developed towards a multi‐mechanism and more precise direction. Hu et al. successfully synthesized NiCoTi‐LDH nanosheets, achieving pH‐responsive PDT/CDT synergy through oxygen vacancy regulation. The photogenerated electrons were used to accelerate the cycling conversion of Co^3+^/Co^2+^, thereby enhancing the Fenton reaction and converting endogenous H_2_O_2_ into highly toxic ·OH.^[140]^ This enables the efficient production of reactive oxygen species without the need for additional photosensitizers, and the system structure is relatively simple and has good biocompatibility. However, this system is still limited to the level of ROS regulation and has limited direct activation ability for immune responses (Figure 13a). Gong et al. constructed a novel nano‐delivery system based on zinc‐copper‐aluminum layered double hydroxide (ZCA‐LDH), designated as ZCA‐LDH@DSF, for loading disulfiram (DSF).^[143]^ This system enables responsive release of Zn^2+^, Cu^2+^, and DSF in the acidic tumor microenvironment. Through the synergistic action of multiple metal ions and in situ drug activation, it effectively enhances ICD, thereby inhibiting tumor growth and metastasis and overcoming the limitations of conventional single‐ion therapy.

Beyond triggering immune responses via metal ions, relevant immune responses can also be induced by loading enzyme inhibitors. Liang constructed a multifunctional nanoagent (a‐LDH@356) consisting of amorphous LDH (a‐CoW‐LDH) nanosheets loaded with the PAD4 inhibitor YW3‐56, enabling synergistic therapy between SDT and immunotherapy. Under ultrasound irradiation, a‐CoW‐LDH can generate large amounts of ROS. Meanwhile, the loaded YW3‐56 effectively inhibits the elevation of citrullinated histone H3 (H3cit) and the release of neutrophil extracellular traps (NETs), thereby addressing the issue that traditional SDT‐mediated NET formation promotes tumor metastasis (Figure 13b–d).^[144]^ Li's team developed a copper‐ion‐doped layered double hydroxide (Cu‐LDH) and innovatively proposed leveraging the “broken window effect” at the organelle level to amplify the efficacy of cancer immunotherapy.^[145]^ Upon entering tumor cells, Cu‐LDH precisely targets and induces lysosomal rupture. This disruption not only directly blocks the autophagy protective barrier essential for cancer cell survival but also leads to abnormal intracellular accumulation of released copper ions, simultaneously triggering pyroptosis and cuproptosis. This dual lethal mechanism driven by lysosomal disintegration amplifies pro‐inflammatory responses within the tumor microenvironment. Without relying on any exogenous antibodies or chemotherapy drugs, it successfully disrupts the tumor's immunosuppressive environment and achieves efficient systemic immune responses. However, the recruitment and activation of immunosuppressive cells, especially macrophages, are inevitably initiated, which makes the immunotherapy outcomes for patients receiving radiotherapy less favorable. Dai's team synthesized a simple aluminum‐like adjuvant (bLDH), which improves radioimmunotherapy by inducing cross‐antigen presentation in macrophages.^[146]^ This design, which does not pursue complex functional stacking but instead focuses on addressing specific “immune breakpoints”, provides a new perspective for the construction of future combined treatment strategies.

The research on the multi‐modal treatment system based on LDH has undergone a development process from physical‐chemical synergy to the integration of immune‐metabolic regulation. By leveraging the advantages of the PTT/CDT system in local killing, the introduction of SDT significantly improved the tissue penetration but still relied on the efficiency of ROS; and further combining immune regulation and metabolic intervention could activate systemic anti‐tumor immunity on the basis of local treatment. Thus, it can be seen that the multi‐modal synergy not only achieves complementary advantages but also provides an important direction for overcoming the limitations of the tumor microenvironment and improving the long‐term treatment effect.

## 4 LDHS FOR BIOIMAGING

As another crucial component in clinical cancer therapy, medical imaging plays a vital role in all stages of cancer management, including cancer screening, therapeutic efficacy monitoring, and tumor recurrence detection.^[147]^ Ideal imaging modalities typically possess the capabilities of being minimally invasive or non‐invasive, convenient for imaging, enabling real‐time monitoring, and providing sufficiently detailed information about target tissues‐including the structure of organs and blood vessels, the physiology of dynamic biological processes, and the biodistribution of drugs‐ranging from the molecular to cellular and/or organ to organismal levels. Over the years, significant advances have been made in a range of cancer imaging modalities (e.g., MRI, fluorescence imaging (FLI), computed tomography (CT), ultrasound (US), and photoacoustic imaging (PAI)), which provide structural information. Additionally, more modern approaches such as PET and SPECT have been developed, offering additional information such as the physiological characteristics of organs and tissues. To date, LDH‐based imaging contrast agents (ICAs) have been extensively studied; due to their tunable host composition and versatile intercalation chemistry, the imaging contrast of LDHs can be achieved by loading imaging functional agents or modifying metal elements in the layers. Currently, LDH‐based ICAs can be categorized into single‐modal ICAs and multi‐modal ICAs.

### 4.1 Fluorescence imaging

Fluorescence imaging is an imaging technique based on the luminescent properties of fluorescent substances. It achieves this by specifically binding fluorescent probes or markers to target biomolecules, cells, or tissues; under irradiation with excitation light of a specific wavelength, fluorescent substances absorb energy and then emit fluorescent signals with longer wavelengths, which are subsequently captured by optical detection equipment and converted into visualizable images. This enables dynamic observation and analysis of molecular‐level events, cellular activities, or tissue structures in vivo. In the biomedical field, fluorescence imaging, leveraging the advantages of high sensitivity, high specificity, real‐time capability, and non‐invasiveness, is widely applied in disease diagnosis, tracking of drug delivery, monitoring of cell migration, and studies on molecular mechanisms among other areas. However, traditional fluorescent dyes still face numerous challenges in practical applications, such as poor photostability, short blood circulation half‐life (typically only 3–4 min), and rapid metabolic clearance by the liver and bile ducts in vivo, which significantly limit their application in long‐term dynamic monitoring and deep tissue imaging. To overcome these limitations, researchers have explored various strategies to improve the performance of fluorescent dyes, among which an effective approach is to load dye molecules (e.g., cyanine dye Cy5.5, FITC) into nanocarriers, leveraging the unique properties of nanomaterials to enhance the stability and biodistribution characteristics of the dyes. As an excellent nanocarrier, LDHs exhibit a layered structure, high specific surface area, and good biocompatibility, enabling them to effectively stabilize and protect fluorescent molecules from harsh environmental factors such as enzymatic degradation, pH fluctuations, and photobleaching, thereby reducing the loss of fluorescent signals. Additionally, surface modification of LDHs can significantly improve the water solubility of fluorescent dyes, avoiding fluorescence quenching caused by aggregation. More importantly, the nanosheet structure of LDHs can promote cellular uptake, accelerating the cellular internalization of fluorescent probes through endocytosis, thereby enhancing the intensity of imaging signals. This property enables it to show great potential in fields such as tumor‐targeted imaging, drug delivery tracing, and cell labeling. In the future, through further optimization of LDH's surface functionalization and composite structure design, it is expected to develop fluorescent imaging probes with higher sensitivity, longer circulation time, and lower background interference, providing a more powerful tool for precision medicine and real‐time integration of diagnosis and treatment.

**FIGURE 14:**
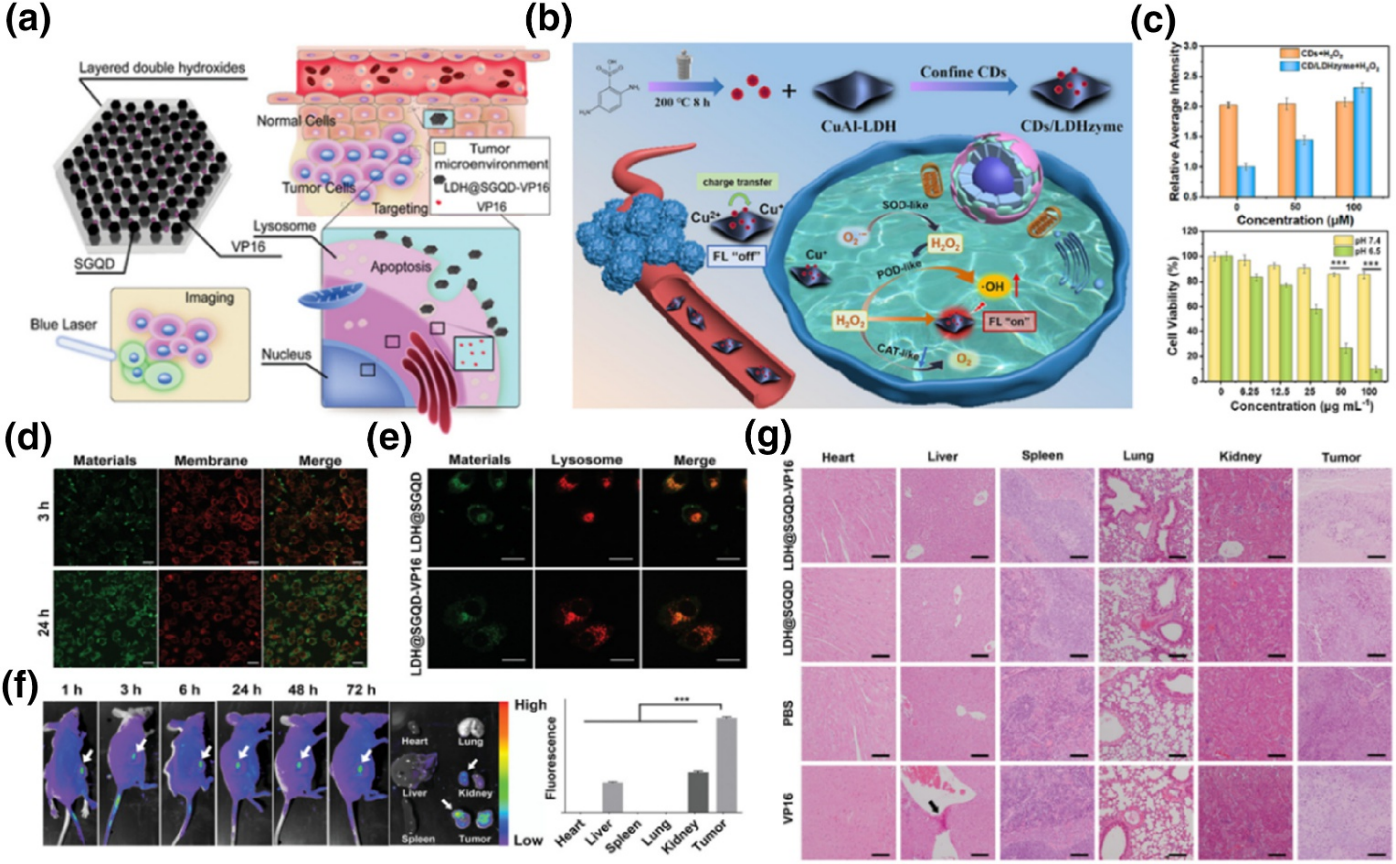
LDH‐based nanomaterials for fluorescence imaging. (a) Trifunctional Graphene Quantum Dot@LDH Integrated Nanoprobes for Visualization Therapy of Gastric Cancer. Reproduced with permission.^[151]^ Copyright 2021, Wiley‐VCH. (b) Schematic diagram illustrating the synthesis process and mechanism of action of carbon dots (CDs)/LDHzyme and (c) The relative viability of HeLa cells co‐incubated with CD and CD/LDHzyme, and then treated with H_2_O_2_ (100 μM) at different pH values. Reproduced with permission.^[142]^ Copyright 2023, Elsevier. (d) Cell membrane dyed by Dil at 543 nm. Scale: 20 μm. (e) Lysosomal co‐localization of LDH@SGQD and LDH@SGQD‐VP16 at 24 h. Scale: 20 μm. In vivo fluorescence images at 1, 3, 6, 24, 48, and 72 h; organs (heart, liver, spleen, lung, kidney, and tumor) at 72 h, (f) post intravenous injection with LDH@SGQD (0.1 mL, 9.2 g L^−1^) visualized at 475 nm and fluorescence quantification using ImageJ. (g) H&E staining images of tumor tissues and other organs (heart, liver, spleen, lung, and kidney) after mice were sacrificed at the 18th‐day post the intraperitoneal injection with various formulas including PBS, VP16, LDH@SGQD, and LDH@SGQD‐VP16. Reproduced with permission.^[151]^ Copyright 2021, Wiley‐VCH.

In 2000, Choy et al. conjugated FITC to MgAl‐LDHs for monitoring the drug‐release behavior of LDHs.^[148]^ Since then, various materials with imaging capabilities have been modified onto LDHs for fluorescence imaging. Zhou and colleagues fabricated a multifunctional CDs/ICG‐uLDHs nanocarrier through the self‐assembly of red‐emissive CDs, ICG, and ultrathin layered double hydroxides. This platform was designed for tri‐modal bioimaging (fluorescence/photoacoustic/two‐photon) and efficient photothermal therapy.^[149]^ In addition, some autofluorescent materials, such as gold nanoclusters (AuNCs), phthalocyanines, and carbon dots (CDs), suffer from drawbacks including low quantum yield in body fluids and short fluorescence lifetime after use. Loading them into LDHs by leveraging the surface confinement effect of LDHs can significantly enhance the quantum yield and prolong the fluorescence lifetime. The Lin group designed a layered double hydroxide‐based nanozyme (CDs/LDH) by confining CDs on the surface of ultrathin CuAl‐LDH, which enabled high‐selectivity and fluorescence “switch‐on” imaging of H_2_O_2_ in the tumor microenvironment (Figure 14b,c).^[150]^ This system utilizes a self‐regulating electron transfer process between Cu^2+^ and CDs to generate fluorescence upon H_2_O_2_ exposure, eliminating the need for complex surface modifications typically required for conventional carbon dot probes, while demonstrating outstanding sensitivity and selectivity. Moreover, the nanozyme exhibits enhanced fluorescence response under weakly acidic conditions, allowing discrimination between cancer cells and normal cells and enabling tumor‐specific fluorescence imaging. Meanwhile, the Wang group constructed a functional nanoplatform, LDH@SGQD‐VP16, based on sulfur‐doped graphene quantum dots (SGQDs) and LDH, achieving visualized therapy and real‐time imaging tracking for gastric cancer.^[151]^ Through electrostatic interaction between SGQDs and LDH, a fluorescence red‐shift occurs (emission peak shifting from 480 to 560 nm), significantly improving tissue penetration and imaging contrast (Figure 14a,d–g). This probe also demonstrates excellent pH‐responsive fluorescence and high photostability both in vitro and in vivo, allowing specific accumulation in the acidic tumor microenvironment and sustaining long‐term fluorescence signals for real‐time imaging and drug distribution tracking from the cellular to the whole‐animal level. Furthermore, the fluorescence intensity correlates positively with the extent of drug accumulation, enabling simultaneous tumor localization, treatment response monitoring, and therapeutic efficacy evaluation. This integrated optical imaging strategy provides a comprehensive solution for precision visualized therapy of gastric cancer.

During the research process, it was found that certain metals also exhibit fluorescent properties. Wei successfully prepared a fluorescent LDH‐PEG composite material by performing surface modification of Tb^3+^‐doped layered double hydroxides with poly (ethylene glycol) methacrylate (PEGMA).^[152]^ Doping with Tb^3+^ endows LDHs with green fluorescent properties; after introducing amino groups via 3‐aminopropyltriethoxysilane (APTES) and immobilizing chain transfer agents (CTAs), controlled grafting of PEGMA is achieved under light irradiation. This method avoids the toxicity of metal catalysts and fluorescence quenching issues, and eliminates the need for complex synthesis and coupling steps of CTAs. The obtained composite material retains the strong fluorescent performance of Tb^3+^, while the modification with PEGMA significantly improves its dispersibility. Observations via laser confocal microscopy show that it can be effectively internalized by cells and localized in the cytoplasm with clear fluorescent signals, providing a novel nanoplatform with high fluorescent stability, favorable biocompatibility, and dispersibility for biomedical applications such as bioimaging and imaging‐guided drug delivery.

### 4.2 Magnetic resonance imaging

Magnetic resonance imaging (MRI) has an irreplaceable position in clinical diagnosis due to its high spatial resolution and excellent soft tissue contrast.^[153]^ However, traditional contrast agents (such as gadolinium‐based complexes) generally have problems such as non‐specific distribution, potential biological toxicity risks, and limited relaxation rates, which restrict their further application. Therefore, the development of alternative systems with both high imaging performance and good biological safety has become a research focus. Against this background, LDH has been gradually introduced into the design of MRI contrast agents due to its layered structure, high specific surface area, and controllable interlayer chemical environment, significantly enhancing imaging performance through ion doping or interlayer loading.

**FIGURE 15:**
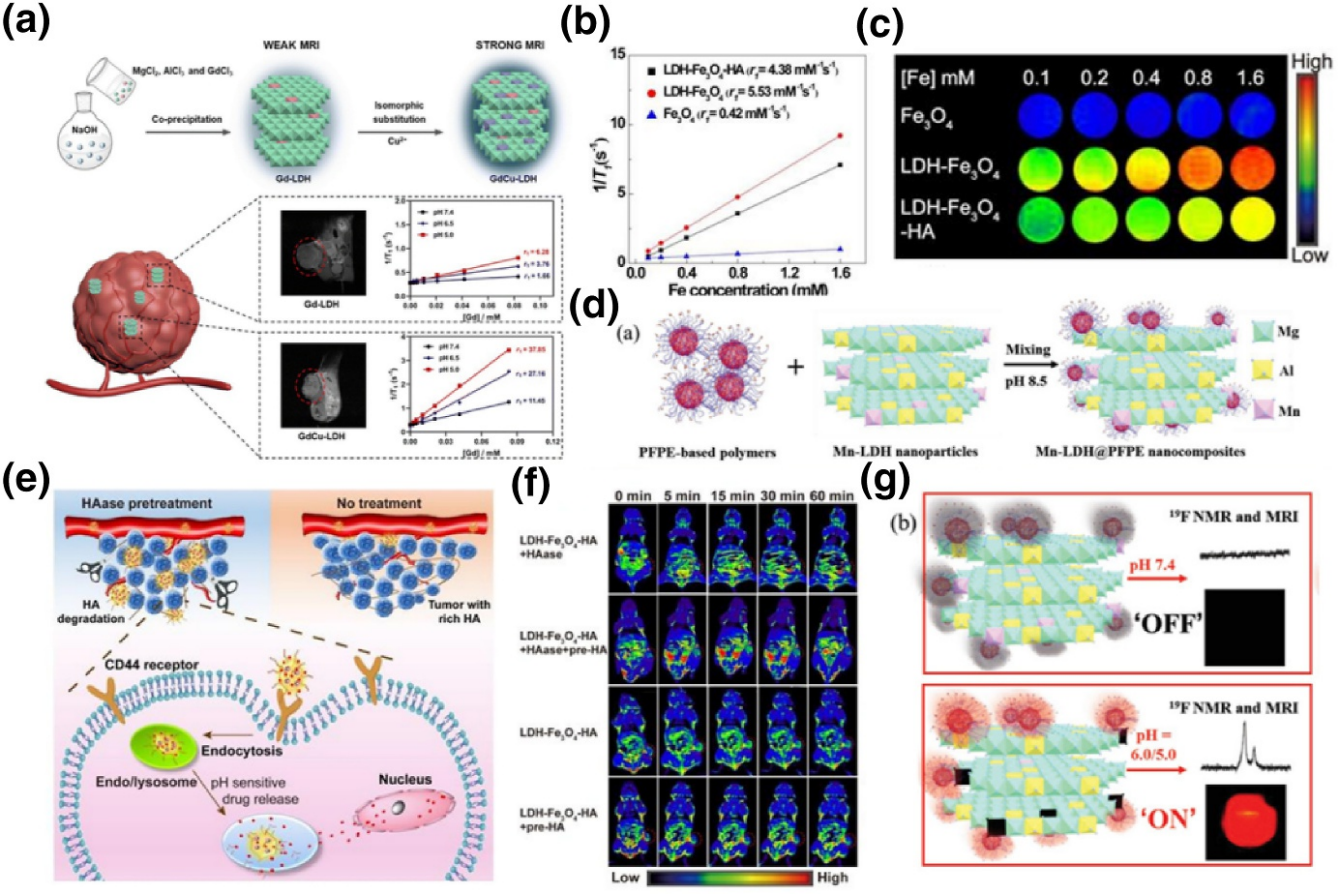
LDH‐based nanomaterials for Magnetic Resonance Imaging. (a) Synthetic procedure and in vivo MRI performance of Gd‐LDH and GdCu‐LDH nanoparticles. Reproduced with permission.^[154]^ Copyright 2023, Royal Society of Chemistry. (b) Linear fitting of 1/*T*_1_ versus Fe concentration in Fe_3_O_4_, LDH‐Fe_3_O_4_, and LDH‐Fe_3_O_4_‐HA, and (c) *T*_1_‐weighted MR images. The color bar from blue to red indicates a gradual increase in MR signal intensity. Reproduced with permission.^[145]^ Copyright 2020, Ivyspring International Publisher. (d) Schematic illustration of Mn‐LDH@PFPE. Reproduced with permission.^[158]^ Copyright 2019, Wiley‐VCH. (e) Synthetic procedure and theranostic mechanism of LDH‐Fe_3_O_4_‐HA/DOX nanoplatforms. (f) In vivo *T*_1_‐weighted MR images of LDH‐Fe_3_O_4_‐HA + HAase, LDH‐Fe_3_O_4_‐HA + HAase + pre‐HA, LDH‐Fe_3_O_4_‐HA + pre‐HA and LDH‐Fe_3_O_4_‐HA ([Fe] = 500 μg/mL, 0.2 mL saline for each mouse). Reproduced with permission.^[156]^ Copyright 2017, Wiley‐VCH. (g) In vitro and pH‐activated properties of Mn‐LDH@PFPE nanoparticles. Reproduced with permission.^[158]^ Copyright 2019, Wiley‐VCH.

Xu et al. utilized Gd‐DTPA for MRI imaging by intercalating it into the interlayer spaces of LDHs (Figure 15a).^[154]^ Under the same conditions, compared with free Gd‐DTPA, the longitudinal proton relaxivity of Gd‐DTPA‐LDHs increased by 4‐fold, while the transverse proton relaxivity increased by 12‐fold. In addition, by taking advantage of the “proximity effect” between the two paramagnetic metal ions, Gd^3+^ and Cu^2+^, the contrast of *T*_1_‐weighted magnetic resonance imaging was significantly enhanced. The longitudinal relaxivity of these nanoparticles was 5–20 times higher than that of single‐metal‐doped LDH nanoparticles and substantially exceeded the simple sum of their individual relaxivities. This result indicates that the proximal arrangement of Gd and Cu at the atomic level cooperatively interacts with surrounding water molecules, markedly reducing proton relaxation time. The LDH‐Fe_3_O_4_‐HA nano‐platform constructed by Guo et al. not only achieved *T*_*1*_‐weighted MRI enhancement, but also endowed it with active targeting ability through HA modification.^[155]^ Moreover, it was loaded with DOX to achieve “imaging‐treatment” integration (Figure 15b,c,e,f). However, the relaxation rate of the iron‐based system is relatively low, and usually, structural optimization or composite design is needed to make up for the insufficient imaging sensitivity of this system. Xu's team constructed an Mn‐LDH‐based gene therapy delivery system utilizing Mn‐LDH as an MRI contrast agent with an *r*_1_ value reaching 4.47 mM^−1^ s^−1^, which is higher than that of commercial Gd complex‐based contrast agents (*r*_1_ = 3.4 mM^−1^ s^−1^), exhibiting favorable tumor imaging efficacy.^[156]^ Zhou prepared amorphous Mn‐CoMo‐LDH nanosheets as efficient SDT nanoagents guided by MRI.^[157]^ The a‐Mn‐CoMo‐LDH nanosheets possess excellent ROS generation capacity; the doped Mn^4+^ can not only alleviate intratumoral hypoxia by decomposing H_2_O_2_ to generate O_2_ but also undergo a redox reaction with GSH to form Mn^2+^, endowing them with excellent *T*_*1*_‐weighted MRI contrast ability and realizing theranostic integration. The introduction of Mn^2+^ not only serves as a contrast agent to achieve *T*_*1*_‐weighted imaging of tumor sites but also synergistically enhances therapeutic efficacy by consuming glutathione, alleviating tumor hypoxia, and other mechanisms. Meanwhile, its release process exhibits an environment‐responsive property, which can improve the specificity and sensitivity of imaging, avoid potential nephrotoxicity issues associated with traditional gadolinium‐based contrast agents, and provide a novel approach for the integration of tumor diagnosis and therapy.

Due to its suitable nuclear magnetic resonance activity (with a spin quantum number of 1/2) and extremely low endogenous ^19^F background signal in the human body, ^19^F is often used to develop ^19^F MRI contrast agents. Zhang designed a novel pH‐activated ^19^F MRI contrast agent‐a MLDH‐loaded perfluoropolyether (Mn‐LDH@PFPE) nanocomposite‐enabling specific and sensitive detection of breast cancer (Figure 15d,g).^[158]^ Under physiological pH (7.4), the ^19^F nuclei in these nanoparticles are affected by paramagnetic relaxation due to their proximity to Mn^2+^, resulting in quenched NMR/MRI signals (OFF state). However, under the acidic conditions of the tumor microenvironment (pH < 6.5), Mn^2+^ partially dissolves from LDHs, weakening the paramagnetic relaxation effect and restoring ^19^F signals (ON state). In vivo experiments showed that after intravenous injection, strong ^19^F MRI signals were detected only in breast cancer tissues, accounting for 92.7% of the total body signal, while signals in normal organs were weak, significantly improving the specificity of tumor detection.

LDHs have become a promising MRI platform; however, some inherent limitations still hinder their practical application. The potential leakage of paramagnetic metal ions (such as Mn^2+^, Fe^3+^) or loaded gadolinium‐based contrast agents raises concerns about long‐term biological safety. At the same time, the dense proton exchange within the interlayer channels and the inhomogeneity of the local magnetic field usually lead to an increase in $T_{2}/T_{2}^{*}$ relaxation, resulting in signal attenuation (negative contrast), thereby impairing the required *T*_1_‐weighted enhancement. Therefore, how to fully utilize the unique structural tunability of LDHs, through coordinated regulation of the metal composition of the layer plates, the interlayer inserted species, and surface functionalization modification to develop MRI contrast agents that have high relaxation efficiency, good biological safety, and in vivo metabolic clearance performance remains the core issue that needs to be solved urgently in this field.

### 4.3 Computed tomography and positron emission computed tomography

Computed tomography, as an imaging technology based on X‐rays, holds a significant position in clinical diagnosis due to its high spatial resolution, rapid imaging capability, and high sensitivity to tissue density differences. It has notable advantages in the detailed imaging of bone structures, lung tissues, and abdominal organs, especially for the detection of fractures, bleeding, and solid tumors. Compared to Magnetic Resonance Imaging (MRI), CT has a greater advantage in imaging bone tissues and air‐containing structures, and it has a shorter scanning time and lower requirements for patient cooperation. However, CT relies on ionizing radiation, and traditional contrast agents lack targeting and specificity, limiting its application in early diagnosis and long‐term follow‐up. Although low‐dose CT and spectral CT technologies have to some extent alleviated the radiation risk and improved imaging capabilities, developing efficient and targeted contrast agents remains a key issue in this field. In this context, the nano‐platform based on LDH has gradually been introduced into the construction of CT contrast agent delivery systems due to its tunable structure and good loading capacity.

**FIGURE 16:**
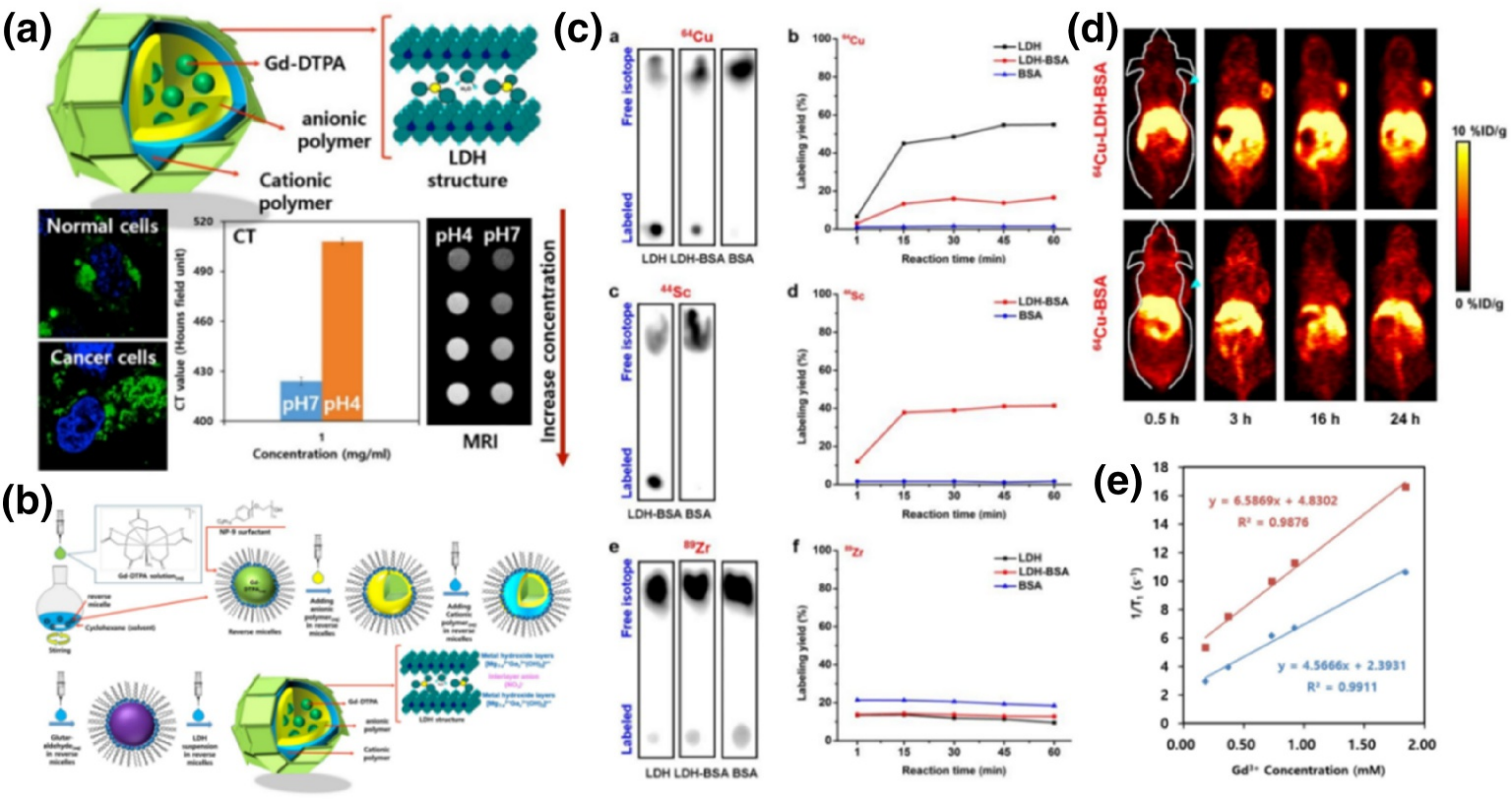
LDH‐based nanomaterials for computed tomography (CT) and Positron Emission CT. (a) Schematic diagram illustrating the mechanism of action of Gd‐DTPA@LDH. (b) The illustration of one‐pot synthesis procedure for the contrasting agent delivery system preparation; anionic polymer is gelatin with high concentration of COOH groups in basic solution and cationic polymer is chitosan in acidic solution. Reproduced with permission.^[159]^ Copyright 2021, Elsevier. (c) (a, c, e) Autoradiographic images of TLC plates of Layered double hydroxide (LDH), LDH‐BSA and BSA after chelator‐free labeling with 64Cu, 44Sc and 89Zr for 60 min. (b, d, f) The labeling yield of LDH, LDH‐BSA and BSA after chelator‐free labeling with 64Cu, 44Sc and 89Zr at different reaction times calculated from autoradiography images of TLC plates. (d) Serial coronal positron emission tomography (PET) images at different time points post‐injection of 64Cu‐LDH‐BSA and 64Cu‐BSA were acquired in 4*T*_1_ tumor‐bearing mice. Reproduced with permission.^[162]^ Copyright 2015, Nature Publishing. (e) MRI *r*_1_ profiles of hyrbrid contrasting agent depending on pH conditions. (The red line represents pH = 4, and the blue line represents pH = 7.) Reproduced with permission.^[159]^ Copyright 2021, Elsevier.

Park's group designed a pH‐responsive and highly efficient CT contrast agent delivery system, which enhances the specificity and sensitivity of CT imaging through unique structural design and intelligent release mechanisms.^[159]^ This system adopts an inorganic‐polymer core‐shell structure with LDHs as the outermost shell, leveraging their property of promoting clathrin‐mediated endocytosis in cancer cells to achieve targeted delivery; the middle layer is a chitosan/gelatin biopolymer composite, and the core encapsulates the Gd‐DTPA complex as the CT contrast agent. It is simply synthesized via a one‐pot method involving continuous fusion of reverse micelles, ensuring structural stability and efficient encapsulation of the contrast agent. Its key innovation lies in the pH‐regulated “on‐off” release mechanism: the release of the contrast agent is significantly inhibited in the physiologically neutral environment, while it is rapidly released in the acidic environment of cancer cells or lysosomes. This property enables the effective accumulation of the CT contrast agent at the lesion site, addressing the background interference issue caused by the non‐specific distribution of traditional contrast agents (Figure 16a,b,e).

However, CT imaging fundamentally relies on density differences, and it still has inherent limitations in functional imaging and the acquisition of metabolic information. To overcome this limitation, PET as a highly sensitive molecular imaging technique has been introduced for collaborative application. PET uses radioactive nuclides to label metabolic substrates and can reflect the metabolic activity state within the organism, offering unique advantages in the early diagnosis of tumors.^[160]^ However, traditional PET probes usually rely on chelating agents for the stable labeling of radioactive nuclides, and different nuclides require specific coordination systems, which increases the design complexity and limits its universality. Therefore, the non‐chelating agent labeling strategy based on nanomaterials has gradually gained attention. LDH, due to its unique layered structure and metal coordination ability, provides an ideal platform for non‐chelating agent PET imaging.^[161]^

As shown in Figure 16c,d, Cai synthesized Mg_2_Al‐LDH and then simply mixed it with Cu, Sc, and Zr, comparing the in vivo PET imaging effects of chelator‐free labeled LDH nanoparticles with radionuclides.^[162]^ It was found that LDH nanoparticles modified with Cu^2+^ and Sc^3+^ exhibited favorable labeling efficiency and stability. However, this method also has certain limitations. For instance, Zr^4+^ cannot be effectively labeled due to its mismatch with the LDHs lattice structure, indicating that the applicability of this strategy is still limited by the structural compatibility of the metal ions. Overall, CT and PET have significant complementary imaging mechanisms: CT provides high‐resolution anatomical information but lacks molecular specificity; PET has extremely high sensitivity and can reflect metabolic processes, but has low spatial resolution and complex probe design. The multifunctional nanoplatform based on LDH, by integrating CT contrast agents and PET radioactive nuclides, is expected to achieve the synergy of structural imaging and functional imaging, thereby compensating for the shortcomings of a single imaging mode. This multimodal imaging strategy not only improves diagnostic accuracy but also provides important support for image‐guided precise treatment.

### 4.4 Multi‐modal imaging

**FIGURE 17:**
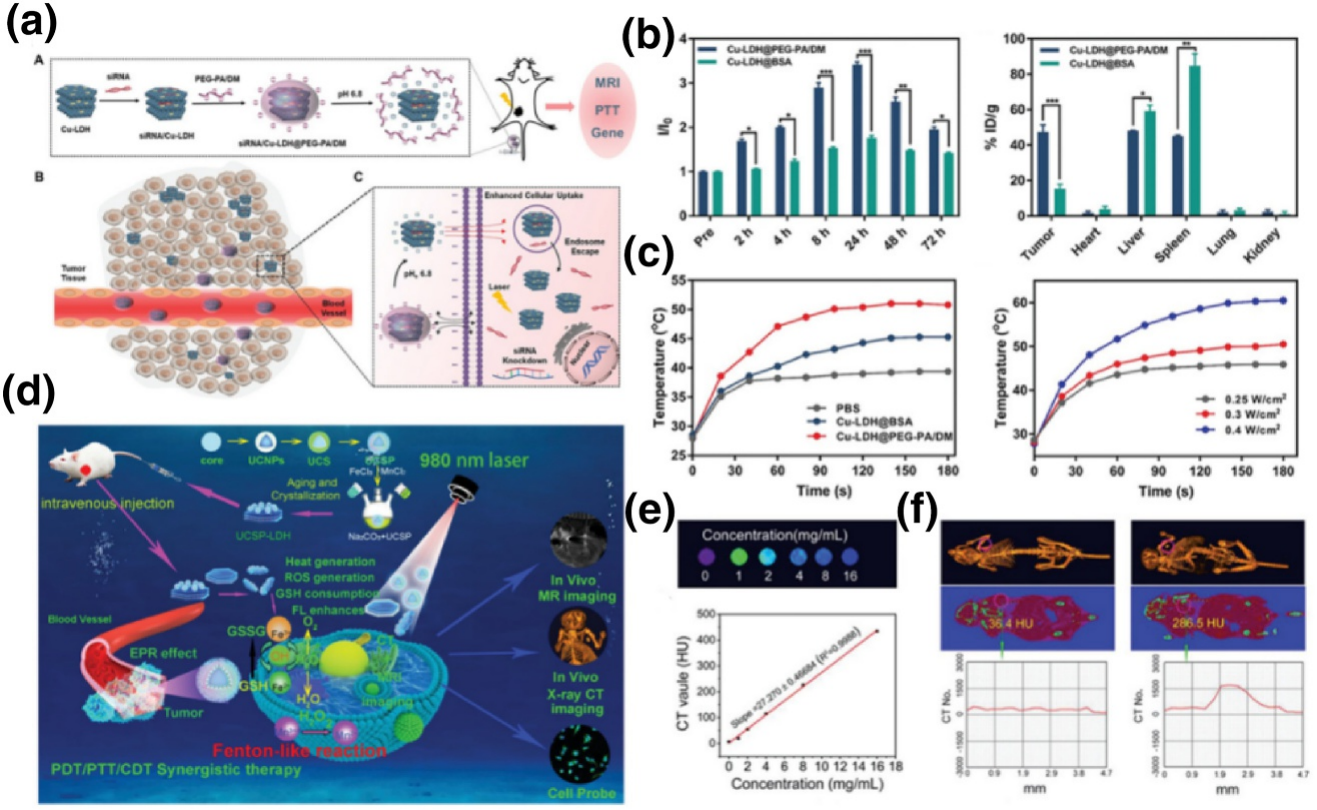
LDH‐based nanomaterials for Multi‐Modal Imaging. (a) Schematic diagram illustrating the synthesis process and functional mechanism of siRNA/Cu‐LDH@PEG‐PA/DM. (b) MRI signal intensity with the time post iv injection. I0 and I: MRI signal intensity of the mice before injection and at a specific time point post injection, respectively; and the Cu level in tumor, heart, liver, spleen, lung, and kidney collected from mice iv injected for 24 h with Cu‐LDH@PEG‐PA/DM and Cu‐LDH@BSA. (c) Tumor temperature profiles of B16F0 tumor‐bearing mice upon 808 nm laser irradiation at 0.3 W/cm^2^ for 180 s at 24 h post iv injection of PBS, Cu‐LDH@BSA and Cu‐LDH@PEG‐PA/DM; And tumor temperature profiles of B16F0 tumor‐bearing mice upon 808 nm laser irradiation at 0.25, 0.3 and 0.4 W/cm^2^ for 180 s at 24 h post iv injection of Cu‐LDH@PEG‐PA/DM. The *t*‐test was used to analyze the statistical significance, *p < 0.05, **p < 0.01, ***p < 0.001. Reproduced with permission.^[165]^ Wiley‐VCH. (d) Schematic diagram illustrating the mechanism of UCSP‐LDH for combined PDT/PTT/CDT therapy. (e) In vitro computed tomography (CT) images of UCSP‐LDH solutions at different concentrations. (f) In vivo CT images of tumor‐bearing mice pretreatment and post‐treatment by injection of UCSP‐LDH and the corresponding cross‐sectional compositional line profiles of CT value. Reproduced with permission.^[164]^ Copyright 2020, Wiley‐VCH.

The application of a single imaging modality in tumor diagnosis and treatment is often restricted by its inherent physical mechanism. For example, fluorescence imaging is restricted by shallow tissue penetration depth (usually only 1–2 cm) and autofluorescence interference, making it difficult to apply to deep tissue detection. Although MRI imaging exhibits excellent soft tissue contrast, it has drawbacks such as long scanning time (typically 30–60 min), expensive equipment, and incompatibility with patients with implanted metals. PET imaging, despite its ultra‐high sensitivity and quantitative analysis capability, suffers from low spatial resolution (usually 4–5 mm), the need for radioactive tracers, and high equipment operation costs. These intrinsic drawbacks mean that single imaging modalities often require complementary integration in clinical applications, while multi‐modal imaging technology overcomes these limitations by integrating the advantages of various modalities, significantly improving the precision of disease diagnosis and treatment. The multifunctional nanoplatform based on LDH provides an ideal carrier for multimodal imaging due to its adjustable structure and component integrability. Current research has gradually shifted from simple functional integration to multi‐mechanism synergy, especially focusing on the construction of LDH‐based nanoplatforms that integrate multimodal imaging and therapy. The research team led by Duan prepared single‐layer LDH nanosheets co‐doped with rare earth elements Gd^3+^ and Yb^3+^ using a top‐down method.^[163]^ This nanomaterial not only exhibits excellent dual‐modal imaging performance but also effectively addresses the stability issue of the chemotherapeutic drug SN38 in higher pH environments by loading SN38 onto LDHs. After further introducing the fluorescent dye ICG, the constructed LDH‐based nanocomposite system achieved synergistic trimodal imaging (CT/MR/near‐infrared fluorescence), significantly improving the sensitivity and imaging accuracy for the detection of deep‐seated tumor cells. However, research indicates that traditional fluorescence imaging is still limited by light scattering and penetration depth, and its signal resolution capability in complex tissues still has room for improvement. To address this issue, Lin developed a TME‐responsive intelligent nano‐catalyst, UCSP‐LDH, which achieved oxygen‐enhanced synergistic therapy and multimodal imaging by anchoring upconversion nanoparticles (UCNPs) onto FeMn‐LDH nanosheets.^[164]^ In the acidic tumor microenvironment, UCSP‐LDH degrades and releases Fe^3+^ and Mn^2+^ ions; Fe^3+^ enhances *T*_2_‐weighted MRI signals through Fenton‐like reactions, while Mn^2+^ and Gd^3+^ significantly improve *T*_1_‐weighted MRI signals. And, the upconversion luminescence (UCL) generated by UCNPs upon excitation with near‐infrared light is restored after the degradation of FeMn‐LDH, achieving high‐resolution fluorescence imaging. Meanwhile, Yb^3+^ doped in UCNPs endows the material with excellent CT imaging contrast. By integrating MRI, UCL, and CT imaging, this platform realizes precise localization and real‐time monitoring of tumors, providing a feasible strategy for multimodal imaging‐guided synergistic therapy (Figure 17d–f). The establishment of this system has obvious advantages over traditional fluorescence systems in terms of imaging depth and signal‐to‐noise ratio. But its structural complexity also imposes higher requirements on material design and stability. Based on the improvement of imaging capabilities, Xu addressed the balance issue between long circulation and efficient cellular uptake of nanocarriers. As shown in Figure 17a–c, the team designed a layered double hydroxide nanoheterostructure with a pH‐sensitive and reversible charge property, which is coated with a polymer layer.^[165]^ Under normal physiological conditions, the negatively charged PEG‐PA/DM on the surface can reduce protein adsorption, prolong blood circulation, and decrease the clearance by the mononuclear phagocytic system. In the tumor microenvironment, the polymer hydrolysis causes the surface charge to reverse to positive, thereby enhancing the EPR effect, electrostatic interaction, and cell uptake efficiency. Meanwhile, the Cu‐doped LDH possesses both *T*_1_‐weighted MRI contrast function and photothermal conversion capability, and the loaded siRNA enables RNA interference therapy.

By introducing elements with higher atomic numbers, such as gold, on the basis of LDHs, the absorption ability for X‐rays can be enhanced. The research team led by Wang developed a Gd‐doped MgAl‐LDH/Au nanocomposite (LDH‐Gd/Au), constructing a theranostic platform integrating CT/MRI bimodal imaging and anticancer drug delivery, thus overcoming the previous limitation that LDH‐based materials had not been applied to multimodal imaging and co‐delivery of drugs.^[166]^ In terms of material structure design, Gd^3+^ was doped into the metal hydroxide lattice of LDHs via a coprecipitation method to serve as MRI contrast agents, while Au nanoparticles were modified on the surface as CT contrast agents. This hierarchical structure efficiently integrates the two contrast functions, and after acid etching treatment, the specific surface area of the material significantly increased to 112.9 m^2^/g, enhancing the contact between water molecules and Gd^3+^ and improving the MRI relaxivity (*r*_1_ = 6.6 mM^−1^s^−1^), which is superior to commercial contrast agents. Therefore, a single or simple stacking strategy is unable to fully meet the needs of tumor diagnosis and treatment. However, by integrating the imaging and therapeutic functions in an organic manner, the complementary advantages of different mechanisms can be achieved, thereby achieving a synergistic improvement in imaging accuracy, treatment efficiency, and biological responsiveness.

From this, it can be seen that LDHs optimize the imaging effects of methods such as MRI, CT, and PET through their unique two‐dimensional layered structure, abundant hydroxyl groups on the surface, and interlayer confinement effects. In terms of enhancing the longitudinal (*r*_1_) or transverse (*r*_2_) relaxivity for MRI, the highly hydrophilic ‐OH on the LDHs surface can effectively guide and concentrate water molecules, bringing them into close proximity to the magnetic contrast agents (such as ultrasmall iron oxide nanoparticles or paramagnetic metal ions like Gd/Mn) loaded on LDHs or doped into the layers. This structure significantly increases the probability of interaction between water protons and the magnetic center as well as the exchange frequency, thereby markedly enhancing the magnetic resonance relaxation rate and imaging contrast at the tumor site. Regarding CT imaging enhancement, the core advantage lies in the unique role of LDHs as nanocarriers. On the one hand, the layered structure of LDHs can efficiently load contrast agents containing elements with high X‐ray attenuation coefficients, such as iodine and gadolinium, forming a stable nanodelivery system that prolongs blood circulation time and enhances targeted accumulation, thereby markedly improving the imaging contrast of lesion areas and overcoming the rapid metabolism of small‐molecule contrast agents. On the other hand, by directly introducing metal ions with intrinsic X‐ray attenuation capability, such as gadolinium and nickel, into the LDHs layers or interlayer spaces, LDHs themselves can be endowed with imaging functionality without the need for additional contrast agent loading. This dual mechanism renders LDHs a relatively flexible CT signal‐enhancing platform, with imaging signal intensity readily tunable through modulation of material concentration and structural design. In improving the stability of the coordination structure of radiotracers for PET imaging, LDHs leverage their strong interlayer electrostatic attraction, ion‐exchange properties, and coordination interactions to firmly encapsulate radionuclides or their chelates between the inorganic layers. The physical shielding and chemical confinement effects provided by this rigid layered structure effectively prevent premature leakage of the tracer during blood circulation and block the disruption of radioactive complexes by serum proteins or endogenous competing ligands in vivo, thereby greatly enhancing the structural stability of radiotracers and the precision of tumor‐specific tracing.

In order to comprehensively review the application of LDH in multi‐modal imaging, Table 3 conducts a systematic comparison of various imaging modes based on LDH. Although LDH possesses advantages such as tumor microenvironment responsiveness, multi‐functional integration capability, and surface‐targeted modification, it still faces many bottlenecks during its clinical translation process. For instance, the mechanisms of long‐term metabolism and toxicity in vivo are not yet clear, the design of the multi‐functional system is overly complex, the imaging depth and sensitivity are limited, the EPR effect in clinical applications is unreliable, and there is a lack of unified evaluation standards. To promote the leap of LDH multi‐modal imaging technology to a clinical precision diagnosis and treatment platform, in the future, we should abandon the reliance on unreliable EPR effect and introduce strategies such as biomimetic CM coating or intelligent charge flipping to endow the nanoscale platform with the ability to actively cross the membrane and penetrate deep tissues. On the other hand, we need to further optimize the material design to solve the problems of long‐term accumulation of LDH in the body and signal distortion in complex physiological environments from the source.

**TABLE 3:** Summary and comparison of LDH‐Based biological imaging systems.

Nanoparticle platform	Treatment method	Advantage	Boundedness	Reference
CDs/ICG‐uLDHs	Multimodal Imaging/PTT	Long fluorescence lifetime	The stability needs to be improved.	[148]
LDH@SGQD‐VP16	Fluorescence imaging/Drug tracking	Theranostics	The signal strength may be affected.	[151]
Gd‐DTPA‐LDH	MRI (*T*_*1*_ */T*_*2*_ weighted)	High relaxivity.	Still based on traditional gadolinium‐based contrast agents.	[154]
LDH‐Fe_3_O_4_‐HA/DOX	MRI//Targeted chemotherapy	Theranostics	Relatively low relaxivity	[155]
Mn‐LDH	MRI/Gene therapy	High longitudinal relaxivity	Lack of targeting ability.	[156]
LDH/Gd‐DTPA	CT	Acid‐responsive release; less background interference	Relatively complex structure	[159]
MgAl‐LDH + Cu/Sc/Zr	CT/MIR/NIR + Chemotherapy	Without chelating agent labeling	Poor structural compatibility	[162]
UCSP‐LDH	MRI/UCL/CT	Excellent targeting property	The structural stability is suboptimal.	[164]
PEG‐PA/DM/Cu‐LDH + siRNA	*T*_1_‐MRI + PTT + RNA interference	Intelligent charge tuning	Depend on external stimuli	[165]

## 5 LDHS FOR BONE REPAIR

The skeletal system not only serves as a scaffold for body support, movement, and hematopoiesis but also constitutes a tissue environment susceptible to various pathological processes. Current treatments for bone tumors and bone defects still face multiple challenges. On the one hand, the bone defect repair process is markedly stage‐dependent, involving dynamic processes such as anti‐infection, angiogenesis, and osteogenesis, yet traditional materials often fail to achieve coordinated multi‐stage regulation. On the other hand, in the context of bone tumors and inflammatory environments, acidic conditions, excessive ROS, and chronic inflammation can inhibit stem cell function and hinder bone regeneration. Furthermore, metastatic bone cancer is frequently accompanied by intractable pain resulting from abnormal neural remodeling, and conventional therapies are unable to simultaneously address both analgesic and anti‐tumor needs.

To address the above challenges, researchers first focused on improving the ‘bone regeneration microenvironment optimization. Zhao synthesized two‐dimensional MgAl‐LDH nanosheets using a hydrothermal method and integrated them with β‐tricalcium phosphate (β‐TCP) scaffolds via 3D printing and surface modification techniques to fabricate a novel composite scaffold.^[167]^ This scaffold exhibits a uniform microstructure, appropriate mechanical strength, and favorable biocompatibility. Through the release of Mg^2+^, it simultaneously promotes the proliferation and differentiation of chondrocytes and bone marrow mesenchymal stem cells.

**FIGURE 18:**
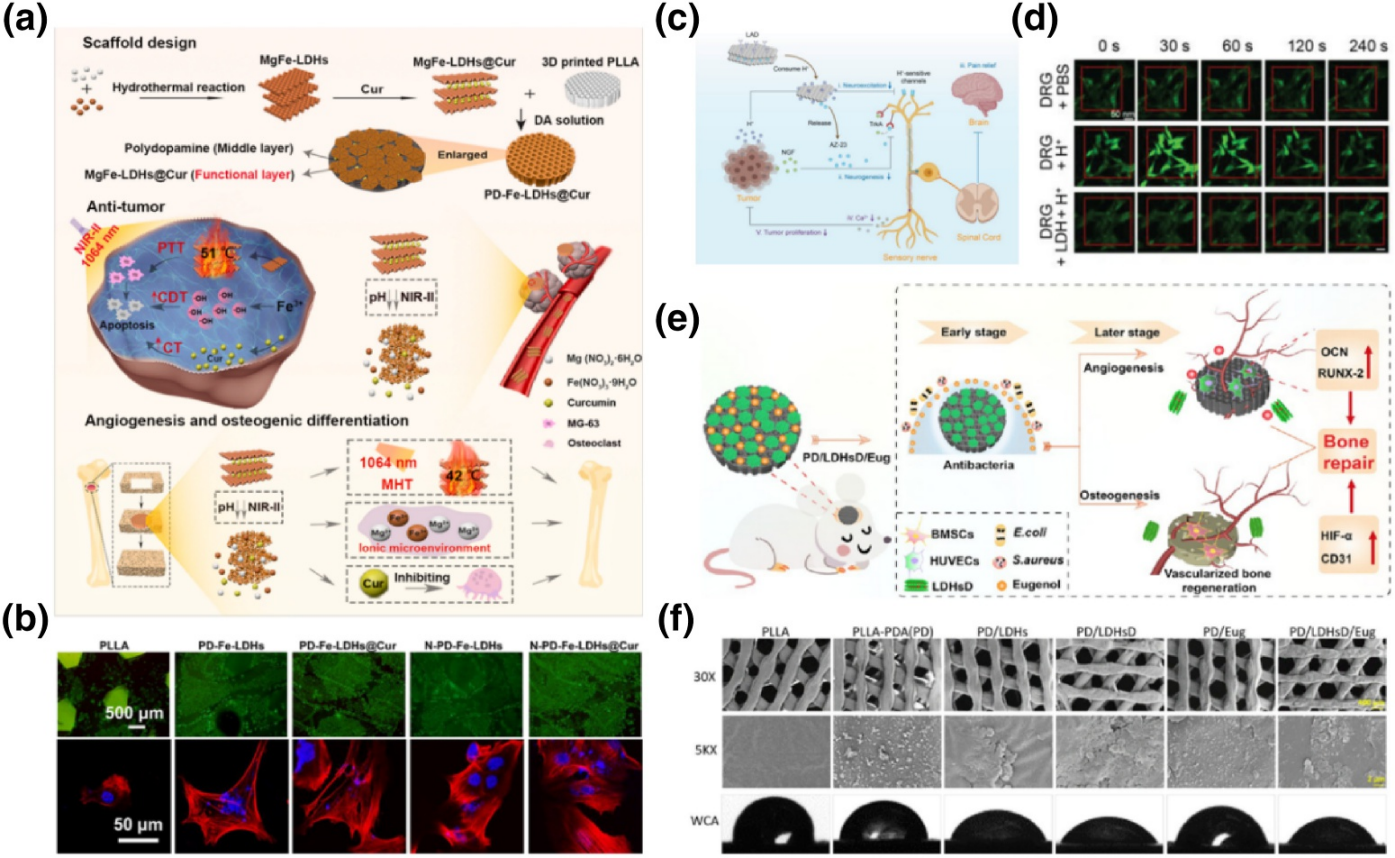
LDH‐based nanomaterials for Bone repair. (a) Schematic diagram illustrating the synthesis process of MgFe‐LDH@Cur and its mechanism in promoting osteogenic differentiation through PTT/CDT/computed tomography (CT) synergistic effects and (b) Fluorescence images depicting the differentiation of osteoblasts under different treatment groups. Reproduced with permission.^[170]^ Copyright 2025, Elsevier. (c) Schematic diagram illustrating the mechanism of action of LAD, and (d) Calcium imaging of DRG cells treated with PBS, H^+^, and LDH+H^+^ at 0, 30, 60, 120, and 240 s. Reproduced with permission.^[171]^ Copyright 2022, Wiley‐VCH. (e) Schematic diagram of constructing versatile 3D‐printed PLLA composite scaffold with spatiotemporal controlled release of DMOG and eugenol and (f) SEM images of the surfaces of six groups of scaffolds under different treatment conditions. Reproduced with permission.^[168]^ Copyright 2022, American Chemical Society.

Implantation of three‐dimensional (3D)‐printed scaffolds is an effective approach for treating bone defects, yet significant challenges remain in their clinical application. Given the dynamic and complex nature of the bone repair process, ideal scaffolds should possess time‐sequential responsive functions: providing effective antimicrobial protection in the early post‐implantation stage to prevent infection; promoting vascular network formation in the middle repair stage to ensure nutrient supply; and sustainably supporting osteogenic differentiation and mineralization in the late stage to ultimately achieve functional bone tissue regeneration. This multi‐stage synergistic design concept provides a crucial direction for the development of next‐generation bone repair scaffolds. Lai constructed a 3D printed composite scaffold modified with LDH/PDA.^[168]^ In this scaffold, the LDHs loaded with osteogenic and angiogenic drugs diaminomethoxyacetic acid (DMOG) and the natural antibacterial agent eugenol were co‐modified with polydopamine (PDA) on the surface of the 3D printed polylactic acid (PLLA) scaffold (Figure 18e,f), achieving a sequential release strategy of “early antibacterial—mid‐term promotion of angiogenesis‐late osteogenesis”. It is noteworthy that the presence of LDH nanosheets enhanced the mechanical properties of the scaffold and improved cell compatibility. Jin‐Ho Choy developed an absorbable bone fixation plate with dual diagnostic and therapeutic functions, which achieved the synergistic effect of X‐ray imaging for diagnosis.^[169]^ Sustained drug release promoted bone regeneration by incorporating the antiresorptive drug risedronate (RS) into LDH nanohybrids. Besides combining them with the biodegradable polymer PLGA to form thin sheets attached to the bone plate, while maintaining favorable biocompatibility and degradability.

Based on the establishment of the multi‐functional scaffold system, the researchers expanded their research to the field of tumor treatment and tissue regeneration synergy. Luo constructed a multifunctional scaffold (PD‐Fe‐LDHs@Cur) with near‐infrared II (NIR‐II) response by modifying the surface of a 3D‐printed polylactic acid (PLLA) scaffold with MgFe‐LDHs loaded with the anti‐cancer drug curcumin. This provided a possibility for the comprehensive treatment of osteosarcoma‐related bone defects.^[170]^ Under NIR‐II light irradiation and in the tumor microenvironment, this scaffold can simultaneously trigger PTT and CDT. Meanwhile, by adjusting the irradiation power, the scaffold can exert a mild hyperthermia effect, which, combined with the effects of Mg^2+^ and Fe^2+^ ions released from MgFe‐LDH degradation as well as the alkaline microenvironment, synergistically stimulates the migration of vascular endothelial cells and differentiation of osteoblasts to promote vascularized bone regeneration. In addition, surface‐modified MgFe‐LDH nanosheets enhance the mechanical properties of the PLLA scaffold, and the scaffold exhibits favorable photothermal stability and biocompatibility. Through the synergy of multiple effects, it achieves the dual goals of postoperative elimination of residual tumor cells and bone defect repair, providing a novel strategy for the treatment of osteosarcoma‐associated bone defects (Figure 18a,b).

As shown in Figure 18c,d, Bu developed a neuro‐cancer crosstalk blocking system based on MgAl‐LDH nanoshells, providing a multi‐target synergistic therapeutic strategy for metastatic bone cancer pain.^[171]^ This nanosystem is surface‐modified with alendronate (ALD) to achieve bone‐targeted delivery, and the internally loaded NGF/TrkA pathway antagonist AZ‐23 can be precisely released upon LDH degradation, fundamentally preventing hyperalgesia and allodynia by blocking cancer‐induced neurogenesis. This system exhibits multiple therapeutic advantages: the LDH nanocarrier can effectively neutralize excessive H^+^ (pH ≤ 4.5) in the tumor microenvironment to directly alleviate pain, while the released Mg^2+^ exerts bone‐protective effects by promoting osteogenic differentiation. Notably, this system establishes a positive feedback loop in therapy‐AZ‐23‐mediated reduced neuronal excitability not only relieves pain but also indirectly inhibits tumor cell proliferation through a Ca^2+^‐dependent mechanism. In vitro and in vivo experiments confirm that this strategy has achieved significant efficacy in both improving pain thresholds and inhibiting tumor growth, opening new avenues for the development of synergistic therapeutic strategies targeting cancer and its associated neurological symptoms. This nanotherapeutic platform, developed from the perspective of neural regulation, not only addresses the clinical challenge of cancer pain but also achieves enhanced antitumor efficacy through the regulation of neuro‐tumor crosstalk, demonstrating important translational medical value.

Inspired by the intracellular antioxidant defense system, Cheng designed a ruthenium‐doped layered double hydroxide as an artificial antioxidant enzyme, enabling efficient redox homeostasis regulation and maxillofacial bone regeneration.^[172]^ Through hydroxyl‐coordinated single‐atom ruthenium catalytic centers, it significantly reduces the activation energy of antioxidant reactions via rapid proton and electron transfer, exhibiting efficient, broad‐spectrum, and stable ROS‐scavenging properties while possessing enzyme‐mimetic activity with excellent cyclic stability. Functionally, Ru‐LDH can effectively protect stem cell viability and osteogenic differentiation in high‐ROS environments, reduce oxidative stress‐mediated DNA damage and cell apoptosis, and maintain cellular redox homeostasis by regulating signaling pathways such as FoxO and HIF‐1α. It downregulates proinflammatory cytokines (TNF‐α, IL‐1β) at inflamed defect sites and promotes the recruitment of endogenous CD44+ stem cells. While accelerating osteogenic differentiation by enhancing the expression of ALP and BMP2/4. LDH‐based materials in the field of bone repair have evolved from early systems focused solely on ion release for osteogenesis into multifunctional therapeutic platforms that integrate microenvironment regulation, drug delivery, immunomodulation, anti‐infection, pro‐angiogenic effects, anti‐tumor activity, and theranostic integration. Their unique layered structure, tunable metal composition, favorable drug loading capacity, and acid‐responsive degradation behavior enable them to play multiple roles in complex pathological settings, including bone defects, inflammatory bone injuries, osteochondral defects, and post‐surgical bone tumor repair.

Active components such as Mg^2+^ and Ca^2+^ improve the bone regeneration microenvironment by promoting osteogenic differentiation, angiogenesis, anti‐oxidative stress, and inflammation regulation. Notably, current LDH‐based bone repair strategies are no longer limited to the conventional goals of “filling defects” or “promoting osteogenesis” but are gradually shifting toward dynamic regulation of the entire bone regeneration process. The incorporation of photothermal therapy, chemodynamic therapy, and diagnostic imaging further meets the clinical need for concurrent elimination of residual tumors and bone defect repair after bone tumor surgery. Moreover, the emergence of biomimetic antioxidant enzyme‐like LDH materials indicates that bone repair materials are transforming from passive supporting implants into intelligent platforms that actively intervene in pathological microenvironments and regulate life processes. However, the clinical translation of LDH‐based materials still faces several key challenges. First, the release rates, local concentrations, and long‐term accumulation safety of different metal ions require systematic evaluation. Second, the degradation behavior of LDHs in vivo environments, the stability of drug release, and their in vivo metabolic pathways need to be further clarified. Moreover, although multifunctional composite systems improve therapeutic efficacy, they also increase the complexity of material composition, preparation processes, and quality control. Therefore, future research should pay greater attention to the structure‐activity relationship between material architecture and biological effects, establish standardized safety evaluation systems, and validate the true repair efficacy using large animal models, long‐term follow‐up, and clinical application scenarios.

## 6 CONCLUSION AND OUTLOOK

As a class of important two‐dimensional nanomaterials, LDHs have shown broad application prospects in the biomedical field in recent years. Ranging from drug delivery, bioimaging to tumor therapy and tissue engineering, their unique layered structure, tunable chemical composition, and favorable biocompatibility provide a solid foundation for their multifunctional applications. The primary concern regarding LDHs in biomedical applications is that their long‐term biosafety has not yet been fully validated. Although short‐term studies have demonstrated that LDHs exhibit favorable biocompatibility at low doses, in vivo degradation behavior, metabolic pathways, and long‐term accumulation effects still require systematic evaluation. Notably, the chemical composition of LDHs (e.g., types of metal ions) and physical properties (e.g., size and surface charge) may significantly affect their interactions with biological systems. For instance, aluminum ions released from the degradation of certain aluminum‐containing LDHs in acidic environments may be associated with neurotoxicity, while excessive surface positive charge could induce non‐specific protein adsorption and CM damage. Addressing this issue necessitates the development of more precise in vitro and in vivo toxicity evaluation models alongside reducing potential risks through surface modification or composition optimization. Furthermore, biomimetic coating technologies (e.g., phospholipid bilayer coating) may further improve the biointerface compatibility of LDHs.

Insufficient drug loading and controlled release performance represent another critical factor limiting the clinical application of LDHs. Although LDHs offer favorable sites for drug loading via interlayer spaces and surface, their capacity is generally lower than that of traditional carriers like liposomes or polymeric micelles. Moreover, loading efficiency remains poor for certain hydrophobic drugs or macromolecular biologics. More problematically, the drug‐release behavior of LDHs in physiological environments often lacks sufficient targetability and responsiveness, leading to premature leakage or insufficient release. To address this limitation, future research can focus on the construction of smart responsive LDH composite systems, for example, achieving tumor microenvironment‐triggered release by integrating pH‐sensitive polymers (such as polyacrylic acid) or redox‐responsive bonds (such as disulfide bonds). Meanwhile, the development of novel intercalation chemistry strategies (e.g., ion‐π interactions or hydrogen bond network regulation) is expected to improve the loading efficiency of hydrophobic drugs. Notably, the interaction mechanisms between LDHs and biological macromolecules still require in‐depth investigation, which is particularly crucial for applications in gene therapy and vaccine delivery. As an emerging nanocarrier, LDHs exhibit unique advantages in the field of combined immunotherapy. Their layered structure can simultaneously load chemotherapeutic drugs, immunomodulators, and nucleic acid‐based drugs, achieving synergistic antitumor effects by promoting antigen presentation and enhancing T cell responses. The core issue in current research lies in how to precisely regulate the interactions between LDHs and the immune system: the intrinsic immunogenicity of the carrier may trigger nonspecific inflammatory responses, while excessive immune activation could lead to severe adverse reactions. Future research should focus on developing smart responsive LDHs systems, achieving sequential release of drugs such as immune checkpoint inhibitors and cytokines through the design of enzyme‐sensitive or redox‐responsive interlayer structures. Surface engineering will be a key direction for optimizing biocompatibility; for example, biomimetic membrane modification can reduce nonspecific phagocytosis by the reticuloendothelial system, while the introduction of targeting ligands can enhance lymph node delivery efficiency. At the mechanistic research level, it is necessary to establish more comprehensive immune evaluation systems and utilize technologies such as single‐cell sequencing to elucidate the dynamic regulatory patterns of LDHs on tumor immune microecology. Through interdisciplinary innovation, LDHs are expected to become an ideal platform for next‐generation combined immunotherapy.

The uniformity of particle size during the production of LDHs also needs to be controlled. Particularly for applications requiring precise control over size and morphology (such as blood‐brain barrier penetration), existing fabrication methods often lack sufficient reproducibility. In order to bridge the gap between the precise design in the laboratory and the clinical application of LDHs nanomaterials, we focus on discussing the scalability challenges in their large‐scale production. From a practical application perspective, the current large‐scale production of LDHs is still far from meeting commercial and clinical standards in terms of yield and batch‐to‐batch reproducibility. There is a significant gap between the laboratory preparation process and the industrial‐scale production. The root cause of this predicament lies in the following: On one hand, the inherent chemical/physical instability of LDHs during the synthesis process makes them highly susceptible to environmental factors. Especially, when it comes to atomic‐level layer charge regulation or intercalation structure design, the difficulty of controllable synthesis increases sharply. On the other hand, most advanced design strategies rely on specific synthetic conditions, lacking the feasibility and economic viability for mass production. Therefore, it is particularly important to establish a bridge between theoretical design and large‐scale implementation. While maintaining structural accuracy, developing simple, robust and scalable preparation methods may be an important direction for promoting the clinical application of LDHs.

The development of LDHs in the biomedical field will present a trend of in‐depth interdisciplinary integration. On one hand, leveraging computational materials science can accelerate the design of high‐performance LDHs; on the other hand, advanced models such as organoids and organ‐on‐a‐chip will more accurately predict the in vivo behavior of LDHs. Notably, the integration of LDHs with other cutting‐edge technologies (e.g., CRISPR gene editing or mRNA vaccines) may give rise to breakthrough therapies. Despite the challenges ahead, through continuous material innovation, mechanistic research, and clinical validation, LDHs are expected to become an important component of next‐generation intelligent nanomedicine platforms, providing new solutions for precision medicine and personalized therapy. This process necessitates not only breakthroughs in basic research but also in‐depth investigations at the levels of molecular pathology and signaling regulatory networks, so as to jointly overcome the “valley of death” from metal‐based materials to clinical applications.

## CONFLICT OF INTEREST STATEMENT

The authors declare no conflicts of interest.

## Data Availability

Data sharing not applicable to this article as no datasets were generated or analyzed during the current study.
